# The sugar industry’s efforts to manipulate research on fluoride effectiveness and toxicity: a ninety-year history

**DOI:** 10.1186/s12940-025-01154-x

**Published:** 2025-09-29

**Authors:** Christopher Neurath

**Affiliations:** American Environmental Health Studies Project (AEHSP), North Sutton, NH United States

**Keywords:** Sugar industry, Fluoride, Fluoridation, History, Science manipulation, Tobacco industry, Caries, Adverse health effects, Smoking

## Abstract

**Background:**

Extensive academic research has documented the tobacco industry's manipulation of science. Recently, scholars have begun examining the sugar industry’s use of similar tactics to downplay sugar’s role in obesity, diabetes, cardiovascular disease, and tooth decay. Archival records show sugar-industry-funded scientists criticized evidence linking sugar to these harms and deflected attention to other risk factors. Sugar’s connection to tooth decay has been the most difficult harm for the industry to deny. Evidence is emerging that the industry turned to promoting fluoride as the solution to tooth decay thereby averting calls for reducing sugar consumption. Newly accessible sugar and dental industry documents enable investigation into whether fluoride research was manipulated to deflect from sugar’s role in tooth decay, and later to defend fluoride when evidence of fluoride’s own harmful effects arose.

**Method:**

Internal documents from sugar and dental organizations were examined and compared to the published scientific record. The Industries Documents collection at the University of California San Francisco was the main source of records. Analysis was in the context of the current understanding of how vested interests manipulate science to defend their products.

**Results:**

Records dating back to the 1930s demonstrate the sugar industry, sometimes in cooperation with dental interests, exaggerated fluoride’s effectiveness and downplayed safety concerns. The sugar industry’s science manipulation campaign preceded the better-known tobacco industry campaign defending cigarettes. Key leaders of the sugar industry’s campaign transferred to the tobacco industry, which then adopted many of the sugar industry’s tactics and financed research from some of the same sugar-conflicted scientists. Currently, a prominent safety issue with fluoride is developmental neurotoxicity. Evidence indicates that researchers with undisclosed conflicts of interest with sugar and allied industries produced biased reviews downplaying this risk.

**Conclusion:**

Recently available records reveal a long history of the sugar industry distorting fluoride science. Many of the sugar industry's tactics were later adopted by the tobacco industry and mirrored by industries involved in asbestos, lead, pesticides, climate change denial, and others. Researchers and policymakers should be aware of the distorted scientific record regarding fluoride effectiveness and toxicity.

**Supplementary Information:**

The online version contains supplementary material available at 10.1186/s12940-025-01154-x.

## Background

Recent scholarly research has uncovered the sugar industry’s efforts to deny or minimize sugar's contribution to obesity, diabetes, cardiovascular disease, and tooth decay by manipulating the scientific record [1–5]. Along with downplaying sugar’s role in these diseases, the industry also sought to deflect attention from sugar by funding research emphasizing alternate risk factors. For example, Kearns and colleagues found that the sugar industry made undisclosed payments to nutrition researchers for a prominent 1967 review in the New England Journal of Medicine that discounted evidence of sugar’s role in cardiovascular disease and blamed dietary cholesterol and fat as the only dietary risk factors worthy of intervention [3].

Of the various adverse health effects linked to sugar, its role in causing tooth decay has been the most difficult for the industry to deny. Kearns and colleagues found evidence that the industry sought to shift attention from reducing sugar consumption to alternative caries preventive methods like fluoridation [2]. Critics of fluoridation, dating back to the 1950s, claimed the sugar industry was behind some of the promotion of fluoridation [6]. Internal industry documents allowing investigation of these accusations have only recently become available [7]. No prior research has examined whether the sugar industry not only promoted fluoridation’s dental benefits but also used science manipulation tactics to defend it against evidence of fluoride’s adverse health effects.

At present, the foremost safety concern for fluoride is developmental neurotoxicity. Rapidly accumulating scientific evidence suggests fluoride has the potential to lower child IQ [8–15]. Systematic reviews by the US National Toxicology Program (NTP) and others have concluded the body of evidence is large and consistent [16–18]. The NTP’s review found statistically significant reductions in child IQ in 46 of 55 studies, with losses averaging 7 IQ points. When the NTP restricted its analysis to 19 studies it rated high-quality, 18 found reduced child IQ. While many of the studies were in areas with water fluoride concentrations greater than those used in artificial fluoridation, several of the high-quality studies were in populations drinking artificially fluoridated water or having similar fluoride exposures from other sources. Based on the NTP review and individual studies, a federal court recently ruled that artificial water fluoridation poses an unreasonable risk of IQ loss in children, and the US Environmental Protection Agency must issue regulations to prevent this risk [19]. Despite this evidence, two recent reviews by a German group concluded fluoridation does not lower IQ [20, 21]. The German review authors declared they had no conflicts of interest. The veracity of this claim will be investigated.

The campaign by the sugar industry to manipulate fluoride science to serve its interests has an unusual feature when compared to better-known campaigns such as those of the tobacco, asbestos, and lead industries, or more recently the fossil fuel industry with climate change [22]. The sugar industry has had a large base of dentists and public health officials enthusiastically championing the promotion of fluoride [23–26]. Most of them are unaware of any industry manipulation of the science. Together with this army of unpaid advocates, the sugar industry has also had a core group of scientists with undisclosed financial ties: “mercenary scientists” in the words of David Michaels, the author of *Doubt is Their Product: How Industry’s Assault on Science Threatens Your Health* [27, 28]. The endorsements by the majority of public health and dental establishments are something other industries would envy for their campaigns. This achievement could have come straight out of public relations guru Edward Bernays’s advice in his 1928 book *Propaganda*. He advised first winning the support of physicians because most people “will follow the advice of their doctors,” or in this case, their dentists [29].^1^

The convergence of corporate, dental, and public health interests has produced a unique case of “bending science” which deserves greater recognition. Both David Michaels and the authors of *Bending Science: How Special Interests Corrupt Public Health Research* recommend transparency as an antidote to manipulated science [27, 31, 32]. Toward that goal, this article seeks to shine a light on the long-running efforts by sugar and allied interests to manipulate fluoride science.

The consequences for public health of delaying acknowledgment of fluoridation’s neurotoxic harm may rival those of the archetypal tobacco and lead industry campaigns to delay regulation of their products. Estimates of the IQ lost from lead in the US for the most-exposed generation born from 1950 to 1980 average about 5 IQ points per person [33, 34]. Converted into economic terms for the US population, this represents about $200 billion per year in lost earnings.^2^ For smoking, an estimate of over $150 billion per year in medical costs and lost earnings was estimated by the Centers for Disease Control (CDC) [36]. Fluoridation may be in the same range of economic costs, with an estimated annual lost earnings from reduced IQ of over $100 billion in the US [37](video at 48:00–49:06, slides 45–46). This far outweighs the claimed economic benefits of fluoridation reducing dental cavities which range from $2 to $8 billion per year [38].

## Methods

The main source of primary documents was the online Industry Documents repository at the University of California at San Francisco (UCSF) [39]. Sub-collections within the repository that contained the majority of relevant documents were the Food, Chemical, and Tobacco Industry collections. Several other online archives containing sugar industry documents were also accessed [40–43]. The Hathi Trust Digital Library was a source of historical books and reports from the sugar industry and the field of dentistry [44]. The Internet Archive was a source of full-text searchable and viewable books as well as archived webpages whose originals are no longer available online, using its Wayback Machine service [45].

Several secondary sources were used to help guide this research and they pointed to additional primary source documents. The secondary sources include recent papers by Kearns and Hujoel on sugar industry manipulation of science and dentistry; the book *The Fluoride Deception* by Chris Bryson which has extensive documentation of US government and fluoride polluting industries’ manipulation of fluoride science; and the Fluoride History website of Peter Meiers [1–5, 46, 47]. Web searches were used to identify information on current organizations and individuals who may be manipulating science surrounding fluoride. Information on contemporary activities was also found in websites of Non-Governmental Organizations (NGOs) in the fields of corporate accountability, environment, and health [48–54].

Analysis of the historical record was in the context of a growing body of research on industry efforts to distort science [22, 27, 55–61]. However, there is less research on science manipulation efforts by interest groups that are not primarily focused on financial gain but rather on promoting specific policies or beliefs [31]. Similarities and differences with other science influence campaigns are examined. Connections between the sugar industry campaigns and those of other industries, when found, were examined.

This history of the sugar industry’s manipulation of science follows several threads that sometimes develop in parallel, and at other times diverge or intertwine. The overall history, and the findings within each thread, will generally be presented in chronological order.

To more easily see connections between people, organizations, and events in a historical context, a visual timeline was created that displays major events, people, and organizations (Additional File 1, Figure S1). In the timeline, major organizations have color-coded horizontal bars, while vertical arrows highlight events connecting different organizations. Another aide for keeping track of the people and organizations is a list of the major actors with short descriptions, provided in Additional File 1, Figure S2.

## Results

### The sugar industry’s reaction to health concerns: manipulate science

**Fig. 1:**
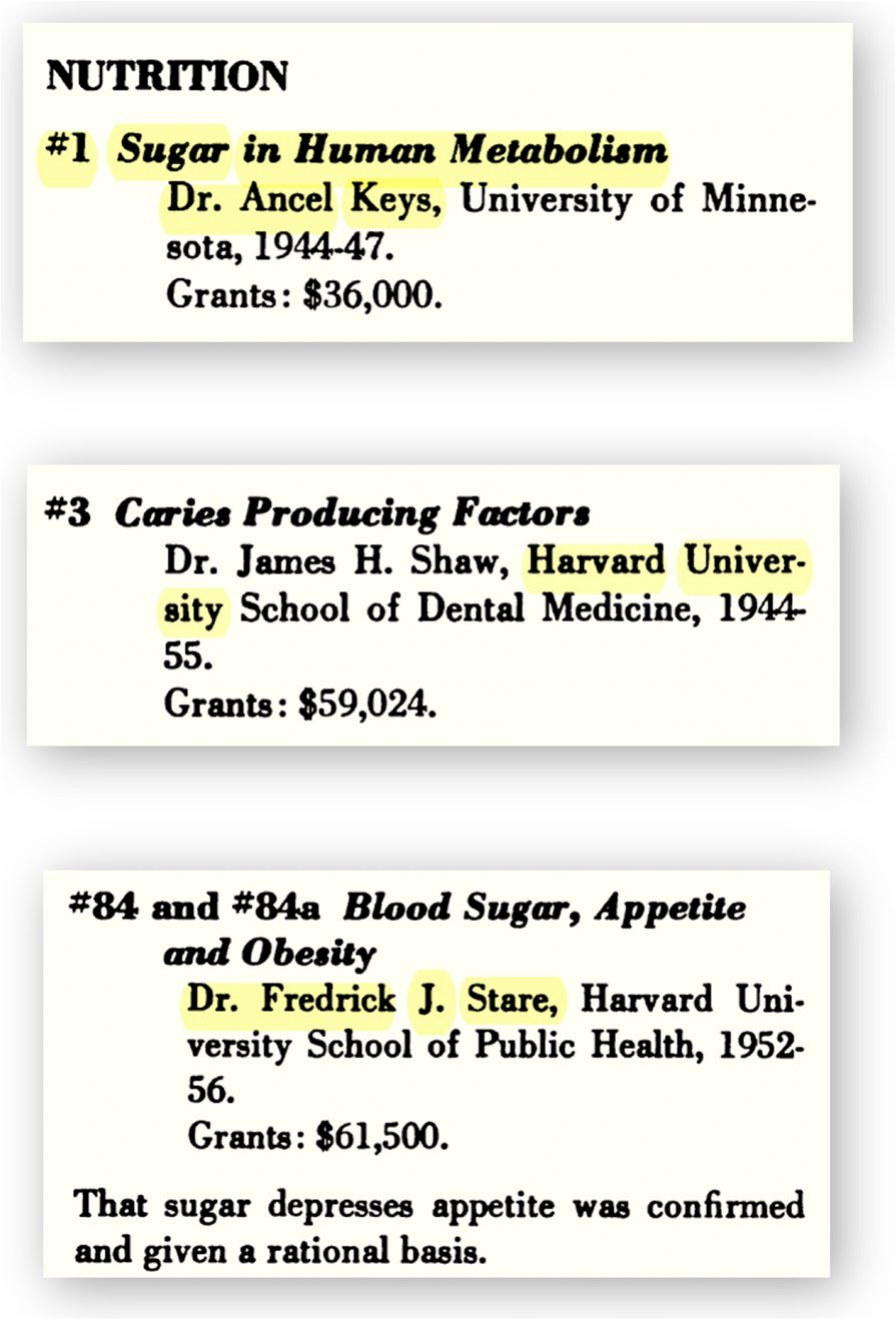
Undisclosed sugar industry funding of Keys, Stare, and Stare’s Harvard colleague Shaw, who was recruited by Stare for the SRF-funded project from 1944–1955 “#3 Caries Producing Factors” [72, 73]. Other SRF documents show Stare continued to receive SRF grants at least through the late 1960s

**Fig. 2:**
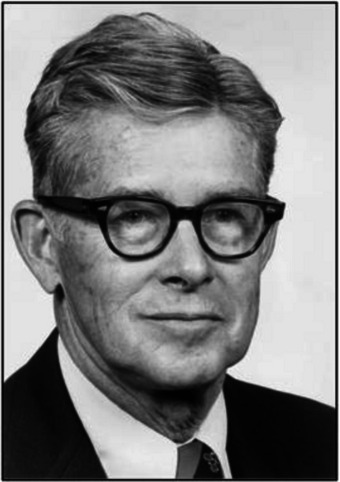
Dr. Fred Stare, founder of Harvard Nutrition Department and ACSH, both funded by sugar and other industries. Image source [82]; used with permission of Elsevier, https://www.sciencedirect.com/journal/the-journal-of-nutrition. The rights to this image are excluded from the Creative Commons CC-BY license

*Pure, White, and Deadly*, a book by British nutritionist John Yudkin, highlighted emerging scientific research from the 1950s onward that linked sugar to obesity, diabetes, and cardiovascular disease [62–64]. The sugar industry considered Yudkin a major threat and responded with intense counterattacks [65]. Yudkin may not have been aware of the secret history of how the sugar industry had quietly funded his main scientific opponent, nutritionist Ancel Keys, starting in 1944 (Fig. 1).^3^ Another scientist who criticized Yudkin and who quietly received sugar industry funding starting in 1944 was Fredrick Stare (Figs. 1 and 2) [67, 68]. Both Keys and Stare vigorously promoted the claim that eating fat was the main cause of obesity and heart disease, and exonerated sugar despite evidence it played a potentially important role [69–71].

**Fig. 3:**
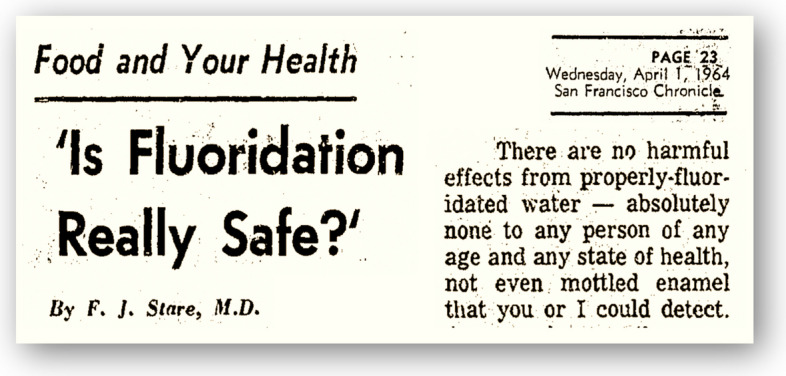
Extracted from one of Fred Stare’s hundreds of weekly syndicated newspaper column articles [80]

While Stare and the sugar industry were promoting the safety and wholesomeness of sugar, they were also promoting fluoridation as the solution to the one health effect the sugar industry could not easily deny: tooth decay [74–76].^4^ Stare had a weekly syndicated newspaper feature called “Food and Your Health” in which he frequently promoted fluoridation. Typical was a 1964 column headlined “Is Fluoridation Really Safe?” where he answered unequivocally: “There are no harmful effects from properly-fluoridated water — absolutely none to any person of any age and any state of health, not even mottled enamel that you or I could detect.” (Fig. 3) [80]. Stare was influencing the science of fluoride as early as 1953, helping to prepare a report by a National Research Council (NRC) committee recommending an “optimal” intake of fluoride to reduce tooth decay [81].^5^

**Fig. 4:**
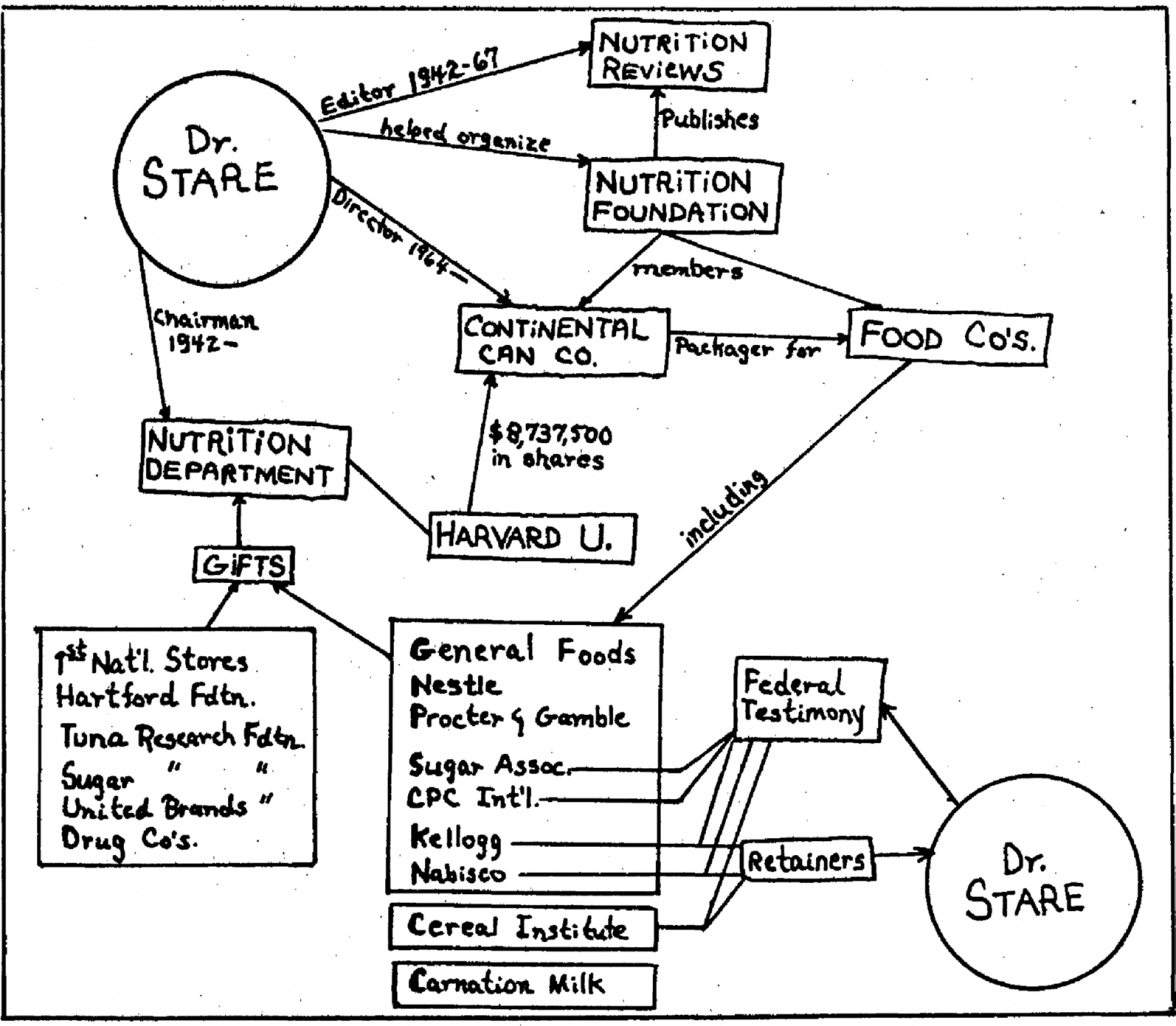
From Harvard Medical School student newspaper, reproduced in [86, 97]

Stare had founded the Harvard Nutrition Department in 1942 and from its beginning funded it largely with donations he solicited from sugar and food industries [83, 84]. Also in 1942, Stare became the editor of the journal *Nutrition Reviews* which was sponsored by The Nutrition Foundation (NF). The Nutrition Foundation had been established just a year earlier, in 1941, by food corporations, including those in the sugar industry, and would maintain a close connection to Stare and his Harvard Nutrition Department for many years.^6^ In 1973 Harvard Medical School students unearthed and publicized some of Stare’s connections to the sugar industry, which Stare often failed to disclose (Fig. 4). The medical students documented a significant conflict of interest involving Stare, who had been receiving annual retainers from Kellogg and Nabisco, the manufacturers of sugary cereals, for more than 15 years. These retainers amounted to a quarter of his salary [86].

As early as 1957, letters from the Boston Nutrition Society to the president of Harvard University had complained that Stare’s nutritional advice seemed to be bought by the food industries that funded him [87]. By 1990 many more industry donors were revealed, with 17 sugar industry organizations or companies and 145 more in the processed food, chemical, and pharmaceutical industries giving tens of millions of dollars to Stare and his Harvard department [83].

In 1978 Stare founded, with his protégé Elizabeth Whelan, the American Council on Science and Health (ACSH), an industry front-group claiming independence yet almost wholly funded by food, chemical, oil, and pharmaceutical companies [88–93]. Ralph Nader called ACSH “a consumer front organization for its business backers” [94]. Over its 50 years of existence, ACSH has consistently defended sugary foods, promoted fluoridation, and attacked the science, scientists, and anyone else who raises questions about the safety of sugary foods or fluoridation [95, 96].

### 1930s: Earliest efforts by sugar industry

#### Gerald Cox at Mellon Institute of Industrial Research seeks “philosopher’s stone” against tooth decay … and discovers fluoride

**Fig. 5:**
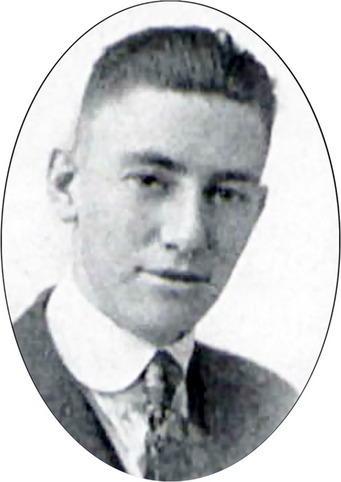
Chemist Gerald J Cox of the Mellon Institute of Industrial Research. Cox was contracted to study dental caries in rats for the Sugar Institute, Inc. in 1930 and was the first person to publicly suggest fluoridation of municipal water systems in 1939. Image in public domain

In 1930, fourteen years before Keys and Stare got their first sugar industry grants, the industry started funding what was called The Sugar Fellowship at the Mellon Institute of Industrial Research [98]. The Mellon Institute was a private organization that industry could contract to do research. The Sugar Fellowship was intended to produce evidence that would exonerate sugar from causing tooth decay (dental caries) or failing that, find ways to reduce caries without restricting sugar consumption. Chemist Gerald Cox (Fig. 5) led the project and sought to find a substance against dental caries in what he likened to the alchemist’s quest for the magical “philosopher’s stone” that could turn base metal into gold [99, 100]. He initially had no thoughts of fluoride. Cox described his experiments in 1935 in an internal report:

*The investigation was a part of a study of cane sugar in the diet and was sustained by the Sugar Institute, Inc., an association of cane sugar refiners. [The study found] that susceptibility to tooth decay can be controlled in rats by dietary means. Specifically, evidence has been obtained that there exists a substance which if present in adequate amount in the diet of the mother rat during pregnancy and/or lactation the young will not develop tooth decay when later fed a caries producing diet. As the substance appears to be of the nature of a vitamin it has been named, tentatively …"dentamin"as a contraction of"dental vitamin”*.

Cox’s experiments were in rats given dozens of different types of diets, either to the mothers or their offspring. His first experiments found that rats do not naturally develop any caries, no matter how much sugar is in their diet. Thus, Cox had to “induce caries” in them by giving them a hard cracked-corn diet that caused fractures in their tooth enamel that could then develop into caries [101–103]. Once the fractures were created, sugar in the diet would greatly accelerate caries development, whereas a zero-sugar diet would prevent high caries rates [104].

To shift attention from this confirmation that sugar accelerates induced caries, he focused on the diet of the mother instead of her pups. A maternal high-sugar diet would not greatly affect the offspring’s caries susceptibility [105]. During the first five years of his experiments, he found many maternal diet items that were effective against induced caries in the offspring. They included “Increased haliver oil [halibut liver oil], increased Ca [calcium] and P [phosphorus], high fat diet, or meat diet” as well as butter, milk, and whey concentrate (Borden’s “XXX liquor”) [106, 107]. Some of these diet items, when fed to pregnant rats, produced substantial reductions in induced caries in the offspring. For example, supplementation with haliver oil, a rich source of Vitamin D, together with calcium and phosphorus, was able to reduce caries by 45% [108]. A maternal diet high in corn oil reduced caries by 32%, and a maternal meat diet “in imitation of the diet of the Eskimo”, reduced offspring caries by 58% [109]. It is unclear why Cox was not satisfied with these findings of substantial caries reductions, but kept testing hundreds of other diets, sacrificing thousands of animals, in search of an elusive “dentamin”.

There are conflicting accounts of how Cox came to consider trying fluoride [46] (pp. 39–44). Ironically, he first thought fluoride would *increase* caries, so he tried to minimize fluoride levels or bind it with aluminum, expecting such diets to reduce caries [110]. By 1937 he was finding the opposite, that adding fluoride to the maternal diet was reducing cracked-corn caries in the offspring. By 1939 he was convinced he had found his “dentamin”. But when he published his results on fluoride (omitting mention of the other caries-preventive diets) he reported fluoride reduced decay by only about 20% on average, less than several of the other diets he had studied [105]. Even this modest benefit is brought into question by numerous problems with Cox’s study designs.

In many of his trials, more than one factor differed between the control group and the treated group, so it was difficult to determine which factor may have played a role in altering caries rates. This problem was noted by external experts asked to review Cox’s work in 1935 when the Sugar Institute, Inc. was terminating its funding and Cox was seeking funding from a charitable organization, the Buhl Foundation. Officials at Buhl asked independent experts in nutrition research to review Cox’s studies. One of them, the head of the Pennsylvania State College Agriculture and Biochemistry Department, responded “while I should like to be able to give it my endorsement, there are so many doubts raised in my mind [by the] uncontrolled variables” [111].

Cox’s published paper reporting his fluoride results is filled with caveats, weakly supported assumptions, and contradictory results. One of the three groups of offspring rats with maternal fluoride diet supplementation had a caries rate that was “the highest that we have observed on any ration” [105]. It was even higher than the control groups that received no fluoride. Cox dismisses this contradictory data by suggesting some unspecified nutritional deficiency may have existed in the maternal diet of that particular fluoride-supplemented group. If so, it would be an example of uncontrolled factors, just as had worried the external expert reviewer several years earlier.

Whether or not Cox’s experiments justified it, by September 1939 he would be the first person to publicly propose artificial water fluoridation. His paper did not disclose his connection with the sugar industry [105]. This was six years before the first human fluoridation trials in 1945. Cox went on to vigorously promote fluoridation for the rest of his life [112].

Shortly before he had announced his proposal for water fluoridation, he had drawn up an application to patent fluoridation of water and foods, although it apparently was never filed [113]. After completing his work on diet and caries at the Mellon Institute in 1941 he spent two years writing major portions of a report on caries and its prevention for the National Research Council (NRC) [114, 115]. He emphasized fluoride. After the NRC Cox moved to a job at the Corn Products Refining Co., whose products included dextrose (corn sugar) and sweet malt syrups [116–118]. After his stint at Corn Products Refining Co., he was appointed to a position at the University of Pittsburgh Dental School [116].

Cox’s experiments eventually gave the sugar industry much of what it wanted. Although he concluded sugar did accelerate caries in what he considered poorly developed teeth, he claimed that fluoride in the mother’s diet could produce offspring teeth that were resistant to decay, even when the offspring had a high-sugar diet [119]. Cox gave the sugar industry their “magic bullet” against tooth decay.

Decades later, the National Institutes of Health/National Institute of Dental Research (NIH/NIDR) sponsored the first rigorous human study to see whether prenatal fluoride supplementation of the pregnant mother could reduce caries in their offspring [120]. The study was a double-blind randomized controlled trial (RCT). It found no significant benefit. Recently, the US Centers for Disease Control Oral Health Division (CDC/OH), in court testimony, confirmed that they do not consider prenatal fluoride to significantly reduce caries in the offspring (see Additional File 2) [121]. Cox’s claimed results in rats exposed prenatally, which sparked the first call to add fluoride to drinking water, do not appear to have been borne out in careful human studies.

Cox’s claim that maternal fluoride exposure reduced caries in their children was accepted by many dentists, and was part of the basis for decades of the dominant position that fluoride acted to reduce decay by being incorporated into the developing tooth enamel while the teeth were still below the gums. While the belief was eventually replaced with the present understanding that fluoride reduces decay predominantly by direct contact with erupted tooth surfaces and provides little, if any, dental benefit from fetal and infant exposure before the teeth have erupted [121], the idea has re-emerged with defenders of water fluoridation. For example, in the same federal court case where the CDC admitted they were unaware of any evidence that prenatal fluoride reduced caries, a witness paid to testify in support of EPA’s position that fluoridation was safe and effective disagreed. Dr. Gary Slade, fluoridation advocate and dental researcher, said in his deposition that he believed “the pendulum” had swung back toward the concept of a pre-eruptive benefit mechanism but could not cite any studies of prenatal or early infancy periods to support his opinion [122]. Thus, distorted science that originated partly from the sugar industry converges with present-day dental interests wishing to defend water fluoridation. In contrast, experts in fluoride developmental neurotoxicity have increasingly issued recommendations that pregnant women avoid fluoride, citing the current mainstream consensus that there are minimal dental benefits to offset the neurotoxic risk [11, 13, 123–126].

#### Recently discovered evidence linking sugar industry to fluoridation promotion: Robert Hockett of the Sugar Research Foundation

Evidence of links between the sugar industry and fluoridation promotion were noted as early as 1957 by fluoridation-opposing physicians Exner and Waldbott, and discussed from a social science perspective by Brian Martin in 1991 [6, 127]. But only recently, through work by two ground-breaking dentists, are details of the secret history of how the sugar industry manipulated science to influence doctors, dentists, nutritionists, the public, and government policies becoming widely known from long-buried industry documents. The first dentist is Cristin Kearns, who found records dating back to the 1940s showing the sugar industry set up The Sugar Research Foundation (SRF) to influence science [1–3, 128, 129]. SRF was a successor to the Sugar Institute, Inc., which had contracted for Cox’s work at the Mellon Institute in the 1930s.

### Manipulating science; which came first, sugar or tobacco?

**Fig. 6:**
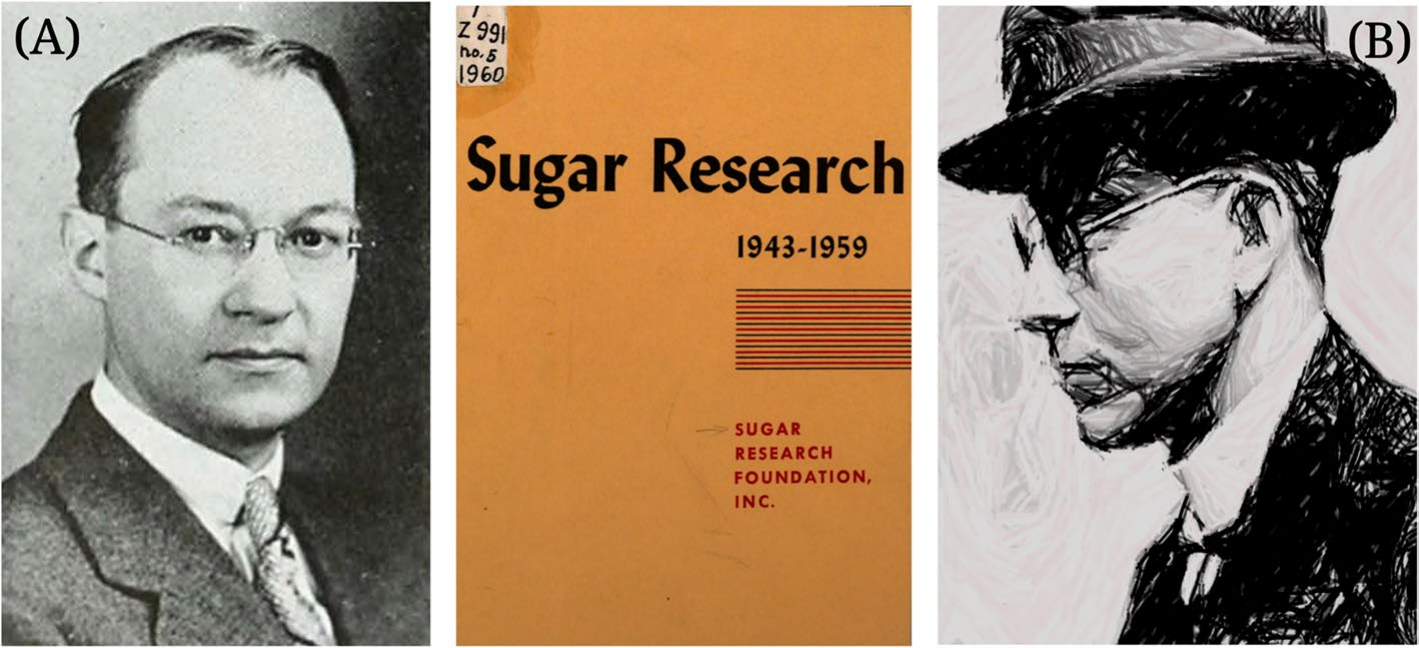
Key people connecting the sugar industry to the dental establishment and tobacco industry. **A** Robert Hockett, Scientific Director of the Sugar Research Foundation (SRF) from 1943 to 1953. In 1954 he switched to become Associate Scientific Director of the Tobacco Industry Research Committee when it first formed and continued there for the next three decades until 1987 [141]. Image is in the public domain. **B** Fice Mork was public relations counsel for ADA in the 1930s-1940s then switched to the SRF soon after it was established in 1944. This image was drawn by the author and is a composite sketch based on several photographs of Fice Mork from the 1930s

The science manipulation methods used by SRF have sometimes been described as originating with the tobacco industry, such as in David Michaels’ book *Doubt is Their Product* [27, 32]. In fact, the sugar industry invented many of the methods that were later transferred directly to the tobacco industry after early studies linking smoking to cancer started raising public concern about cigarettes in the 1950s [130].^7^ Kearns identified the specific person–Dr. Robert Hockett (Fig. 6A)–who led the sugar industry’s disinformation campaign in the 1940s and then offered his services to the tobacco industry when it started coming under attack for causing lung cancer. Hockett^8^ was the science director of the SRF from its formation in 1943 until 1953 and then moved to a similar position at the newly formed Tobacco Industry Research Committee (TIRC) in 1954 where he became a leading tobacco industry apologist for over 30 years. The same day Hockett learned of the new tobacco research organization being formed he applied for the job with a 16-page resume detailing his qualifications. His cover letter boasted [140]:

Dear Sirs:

I note this morning the announcement that an industry committee has been formed to investigate “all phases of tobacco use and health” especially the reputed relation between cigarette smoking and lung cancer. Ten years ago a very similar industry association, The Sugar Research Foundation, Inc., was formed to investigate charges that refined sugar is a primary cause of diabetes, tooth-decay, polio, B vitamin deficiencies, obesity, “mid-morning hypoglycemia” and many other conditions.

[As scientific leader of the Sugar Foundation]... During a period of nine years, I organised and directed research projects in medical schools, hospitals, universities and colleges which exonerated sugar of most of the charges that had been laid against it.... The program also required me, as Scientific Director, to lead and to assist in public relations activities such as symposia, radio programs, preparation of moving pictures, publication of pamphlets etc. I delivered scores of talks and addresses before popular, industry and scientific groups.

The challenge of the present situation to the cigarette industry is so similar to that which I helped the sugar industry to meet, that I am tempted now to suggest that my experience and background may be useful to the new Tobacco Industry Research Committee.

In a follow-up letter by Hockett to the Tobacco Industry Research Committee (TIRC) and the public relations firm Hill & Knowlton who were initially in charge of managing TIRC, he says that he had worked with one of the Hill & Knowlton agents 10 years previously “on the problems of the sugar industry” and the “many delicacies” of funding “academic research by industrial groups” [142]. This is further evidence that the sugar industry’s manipulation of science began well before the tobacco industry started its own self-described “doubt is our product” campaign [143].^9^ The Hill & Knowlton agent Hockett had worked with at SRF was Bert Goss, who became a lead manager of the tobacco industry’s public relations and science manipulation campaigns [145–149].^10^

When Hockett moved from sugar to tobacco he not only brought his own expertise at manipulating science, but he also brought two favored grantees of sugar industry funding with him: Ancel Keys and Fredrick Stare. Both started receiving TIRC grants for projects on tobacco and health [156]. Their sugar industry SRF grants continued as well, so they were simultaneously receiving money from both the sugar and tobacco industries.^11^

A few years later, Hockett arranged to have Stare pass tobacco industry money through the Harvard Nutrition Department to an anthropologist, Carl Seltzer, who was nominally employed by the department [161–164].^12^ Seltzer actually worked full-time promoting a tobacco industry message claiming smoking did not cause heart disease. He argued that there was just a statistical correlation because of a genetic predisposition for certain people to both smoke and develop heart disease.^13^ The tobacco industry and the sugar industry manipulations of science became intertwined, with Hockett, Stare, and Keys playing central roles in this cooperation.

### Sugar is essential ingredient in cigarettes making them more harmful and addictive

There was another link between the sugar and the tobacco industries, a link that played a key role in the rapid increase in cigarette sales starting early in the twentieth century. Sugar was found to reduce the alkalinity of tobacco smoke to make it mild enough to inhale into the lungs, something not typically done with the previous common methods of smoking tobacco in pipes and cigars. “Sugar and tobacco have a long and incestuous history”, says Robert Proctor in his landmark study on the tobacco industry [166] (pp. 30–35).

In one of Hockett’s SRF science bulletins from 1949 an article describes the crucial role of added sugar in making cigarette smoke less irritating [167]. It describes the chemistry of added sugars that produce a less alkaline smoke. Unmentioned is that smoke from cigarettes produces a faster and stronger nicotine response and may lead to greater addictiveness than cigar or pipe smoking [168, 169]. Inhaling the smoke of cigarettes also greatly increases their carcinogenicity [170]. Thus, sugar was an essential component in making cigarettes more addictive and more deadly. The sugar industry apparently knew this by 1949 and the tobacco industry presumably did too [166] (pp. 30–35).^14^ The SRF bulletin’s article on sugar and tobacco notes that “In 1948, 26,000,000 pounds of refined cane and beet sugars found their way into tobacco products.” [167].

In 1950, the SRF commissioned a report by a biochemist/statistician to estimate the market for sugar in the burgeoning cigarette industry. The report, titled “Tobacco and Sugar” confirmed for the sugar industry leaders what was a little-disclosed foundation of the American cigarette industry [171]^15^:

*“Were it not for sugar, the American blended cigarette and with it the tobacco industry of the United States would not have achieved such tremendous development as it did in the first half of this century.” … tobacco and sugar … mutually promote consumer acceptance and consumption.*

The sugar and tobacco industries were intertwined from the early days of commercial cigarettes, and through Hockett and Stare, seemed to directly help each other with funding of “bent science”.

### 1940s-1950s: The sugar industry manipulates American Dental Association positions on fluoride

Following Cristin Kearns’ pioneering investigations of sugar industry manipulation of science and dental policies, another dentist, Philippe Hujoel, extended Kearns’ research with additional historical documents showing the sugar industry influenced American Dental Association (ADA) policies on fluoride. Hujoel is a dental researcher who has long been concerned about mainstream dentistry’s relative disinterest in dietary sugar as an important factor in tooth decay, as well as dentistry’s dismissal of the role of Vitamin D in preventing tooth decay [4, 5, 175, 176]. Hujoel accessed internal ADA records dating back to the 1930s, which he combined with sugar industry documents uncovered by Kearns. He found that by the late-1940s Hockett and the SRF’s public relations consultant Fice Mork had already influenced the ADA into reversing its prior positions on dental caries. The ADA supplanted their previous emphasis on nutritional factors such as excessive sugar consumption and Vitamin D deficiency, with a promotion of fluoride [4, 5].^16^

Hockett and the SRF also seem to have helped sway the ADA to drop its previous concerns about adverse health effects of fluoride. Hockett and his public relations counsel Fice Mork (Fig. 6B), who had previously served as public relations counsel to the ADA, met with ADA executives in October 1944 and obtained agreement from ADA to “cooperate” with SRF [186]. A few months later they met with the incoming new editor of the *Journal of the American Dental Association (JADA)*, Harold Hillenbrand, and found him to be more amenable to the sugar industry’s viewpoint than the editor he replaced. Hillenbrand even offered to “unofficially” inform Hockett about “the standing of various individuals within the profession” suggesting that Hillenbrand was willing to act as an insider ally for SRF [187]. The next year Hillenbrand became executive director of the ADA, a leadership position he would hold from 1946 to 1970 [188]. The sugar industry had found a powerful ally at the center of the dental profession.

In 1942, a few years before the SRF started working with the ADA, an executive from the sugary cereal manufacturer Kellogg’s, named Emory Morris, was made chair of an ADA committee that set the association’s dental health policy [5].^17^ Over the next several years the ADA shifted its position toward a promotion of fluoride as the “magic bullet” that could prevent tooth decay. Simultaneously, ADA de-emphasized the importance of limiting sugar consumption [5]. Morris had been a dentist before becoming a long-time director of the Kellogg Company and President of the WK Kellogg Foundation, which owned a controlling share of Kellogg Company [189–191]. As early as 1942, Morris’s ADA committee had discussed the compulsory addition of fluoride to food as a solution to dental caries [5].

Another connection between Kellogg and the ADA arose in 1945 when the editor of the ADA’s scientific journal *JADA*, Harold Hillenbrand (who was being courted by Hockett and Mork from SRF around the same time, as mentioned above), joined the Kellogg Foundation on its dental advisory committee.^18^ Since Kellogg’s Emory Morris was chair of the ADA committee on dental health policy at the same time ADA’s Hillenbrand joined the Kellogg committee, this suggests a reciprocal arrangement between Kellogg’s and ADA [191]. Hillenbrand supported fluoridation and had just replaced the previous *JADA* editor who had not [5].^19^

According to an account by the dentist Philip Jay, who was dean of the University of Michigan Dental School and a primary advocate for the first human trial of fluoridation in Grand Rapids Michigan in 1945, the Kellogg Foundation gave at least the initial funding for that trial [195]. The Kellogg Foundation has made large financial donations to the ADA, including $250,000 in 1955 (worth $2.8 million today). Over the years the Kellogg Foundation has continued to promote fluoridation through at least 2005, such as with many grants to Latin American programs [196–199].

#### Sugar industry sponsors 1944 symposium promoting fluoridation to thousands of dentists and public health officials

There were other revolving doors between ADA and the SRF, including ADA’s long-time public relations counsel in the 1930s, Fice Mork (see Fig. 6B). Mork started working for SRF soon after it was established in 1943, and then used his dental connections to help push the sugar industry’s agenda, especially with fluoride. One of Fice Mork’s high-level dental connections was his father, dentist Waldo Mork, who was the President of the New York State Dental Society [200, 201].^20^

In a 1945 letter from Fice Mork that was distributed to SRF board members about the causes of tooth decay, he acknowledged sugar’s central role, but ended positively, saying:

The entire question of dental caries, from our point of view, is one that both Dr. Hockett and I have been studying very carefully. We have discussed this for hours at a time and we are developing one or two ideas … this whole story of tooth decay is one that we are now approaching from the right angle. *Fluorine is it*. (emphasis added) [205].

Mork played a critical behind-the-scenes role in getting a special 1944 symposium on fluoride and tooth decay held in New York City that was funded by the SRF [206]. Mork’s father was on the governing board of the New York Institute of Oral Pathology which served as the dental front-group sponsoring the symposium. The symposium was an opening salvo in a public campaign to promote fluoride and fluoridation as the solution to prevent tooth decay. All the founding fathers of fluoridation gave presentations, including dentists Trendley Dean, Fredrick McKay, Wallace Armstrong, and David Ast.

Furthermore, Mork and Hockett arranged for the SRF to pay not just for the symposium but also the substantial cost to print and mail over 100,000 free copies of the symposium proceedings to every dentist in the United States, along with many pediatricians, public health officials, and dental schools. Mork reported to his superiors at SRF how effective this was in boosting enthusiasm for water fluoridation, stating: “Following distribution of this book on fluorine, many local health departments have started agitating for a fluorine program of their own” [207]. The word “sugar” never appears in the 63 pages of the symposium proceedings nor is SRF funding mentioned [208].^21^

The presentation that most explicitly promoted water fluoridation was that by David Ast, Chief of the New York State Health Department Dental Bureau. He announced that a fluoridation trial in Newburgh NY with Kingston NY as the “control” had already started examining children in June 1944. He assured the audience that “Special attention will be given to the questions of … mental development and emotional stability” in children and “Adult examinations will determine the effects, if any, of fluorine in small concentrations on older age groups (past 50 years).” [208] (pp. 43–44). Despite these statements, no studies of child mental development, IQ, or behavior were ever done, nor were studies of the effects of fluoridation on older adults.

The planning committee for the Newburgh-Kingston trial held private meetings. Transcripts of the meetings were uncovered decades later and show they discussed concerns that fluoridation could harm the developing brains of children as well as harm older adults, but they quietly abandoned the planned studies (see Additional File 3). Fluoride neurotoxicity studies in children would not be publicly discussed in the United States until almost 60 years after fluoridation was begun and had been rolled out across 2/3rds of the US population [212]. There still have not been any published studies of fluoride and child IQ in the US, while over 150 developmental neurotoxicity studies have been conducted in other countries in the past 35 years, with the large majority finding harmful effects [18, 213].

In what appears to be a follow-up to the SRF-funded fluoride symposium, the records of the SRF from June 1945 include a press release written by Fice Mork promoting the first-ever human fluoridation and health experiment that was just getting underway in Newburgh NY [214]. The press release makes no mention of Mork’s connection to SRF but instead claims to come from the NY Institute of Oral Pathology, the same dental group that SRF had arranged to sponsor the fluoride symposium. It’s also the same dental group that Mork’s dentist father served on the board of directors. The press release announces a luncheon gathering of several of the fluoridation-promoting dentists who spoke at the symposium, along with a past president of the American Dental Association and numerous public health and military dental officers. The press release starts “NEW YORK- Mass prevention of man’s most common disease, tooth decay, merely by drinking fluorinated water was predicted today by dental leaders at a luncheon given by the New York Institute of Clinical Oral Pathology.” It quotes the dentist leading the Newburgh fluoridation trial, David Ast, as saying the experiment could usher in a dental “utopia”. Then, in a stunt that might have been dreamed up by Mork himself, the press release says the luncheon featured a toast using fluoridated water that had been “especially sent down for the occasion” from Newburgh.

Fice Mork, long-time public relations counsel for the ADA, and SRF’s first public relations counsel, appears to have been central to the sugar industry’s early promotion of water fluoridation. He started with influencing his many dental contacts; then expanded to all the dentists in the USA by mailing the symposium proceedings book to each of them as well as to many public health officials and pediatricians; and eventually reached the general public via news stories and by inciting local fluoridation promoters across the country, who, after reading the symposium proceedings, were “agitating for a fluorine program of their own” [207].

### 1970s: Sugar industry manipulates the NIH National Caries Program

**Fig. 7:**
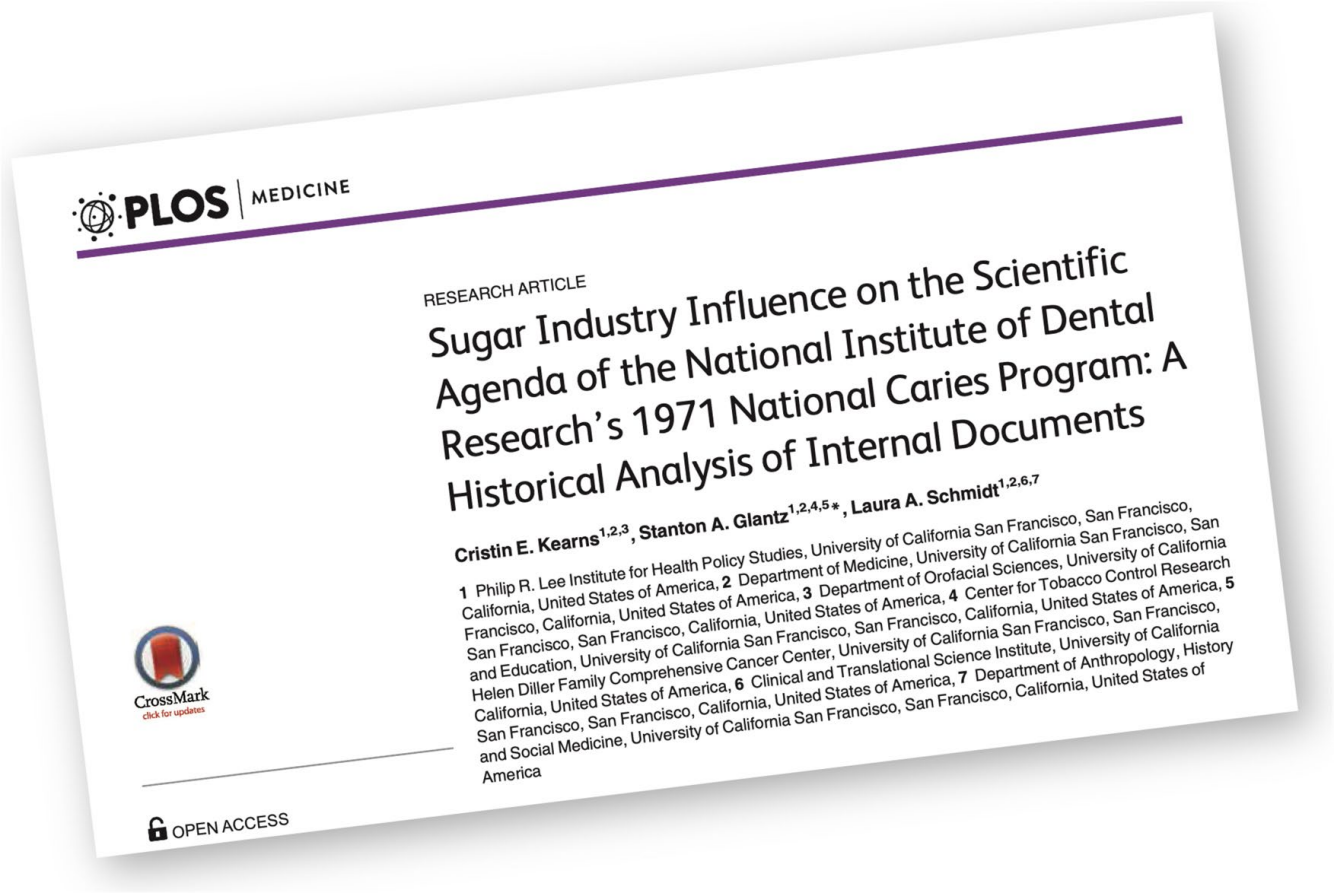
Kearns’ 2015 paper finding sugar industry ghost-writing of the NIH/NIDR National Caries Program. Image used and modified under CC BY license from Kearns et al. 2015 [2]

**Fig. 8:**
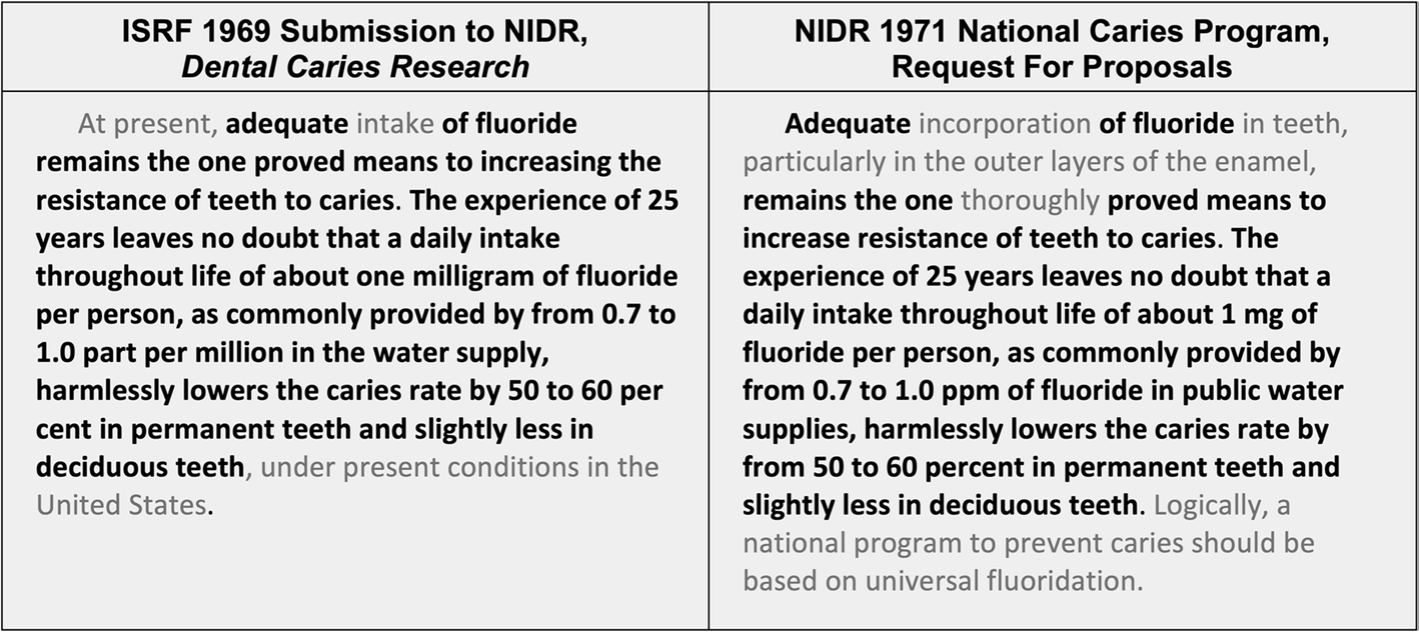
Comparison of text submitted by sugar industry to NIH, and NIH final text. Text in bold font is identical between the two documents. ISRF is the International Sugar Research Foundation, the successor to the SRF. Used and adapted under CC BY license from Kearns et al. 2015 [2]

Kearns’ first paper, published in 2015, showed how the sugar industry manipulated the National Institutes of Health (NIH) National Caries Program of the 1970s (Fig. 7) [2]. The sugar industry essentially ghost-wrote much of the policy agenda issued by the National Caries Program. The policy downplayed the role of sugar consumption and instead recommended a focus on non-dietary interventions, with water fluoridation promoted as the highest priority for addressing caries in America. Kearns puts the text of a submission to NIH by the successor to the SRF (ISRF) side-by-side with the final NIH policy document. The first paragraph of the NIH document, under the heading “Dental Caries”, extolls water fluoridation, copying almost verbatim what ISRF had written, as shown in Fig. 8.

A later example of the sugar industry encouraging the National Caries Program to promote fluoridation is found in the transcript of a 1975 conference in Washington DC sponsored by the ISRF and attended almost solely by sugar industry executives and a few NIH National Institute of Dental Research (NIH/NIDR) staff. The head of the National Caries Program, James Carlos DDS, in a Q&A session lamented the stalled progress in increasing fluoridation in the USA, saying “From now on the battle – and that is a fair term – is going to be extremely hard.” A sugar industry attendee responded “Is this not an area where the National Institutes of Health and the sugar industry might cooperate to promote water fluoridation to the various recalcitrant communities?” [215] (p. 82).

### 1950s-2020s: Corruption at National Academies of Science: reviews of fluoride science conflicted by sugar, food, tobacco, chemical, and pharmaceutical industries’ money

The Sunday, April 23, 2023 edition of the *New York Times (NYT)* ran a front-page exposé revealing the National Academies of Science Engineering & Medicine (NASEM) had accepted $19 million in donations from Sackler family members, the owners of opioid maker Purdue Pharma [216, 217]. Marketing practices of Purdue Pharma have been blamed for much of the opioid epidemic of recent years. During the time of the Sackler donations, NASEM issued an influential report that said patient pain was not being managed sufficiently and recommended wider use of pain medications. Purdue Pharma used this report to promote its opioid products. NASEM is a private organization that receives much of its funding by contracting with the federal government to write reports on scientific, medical, and technical topics. But as a private organization NASEM is exempt from Freedom of Information laws [218], and there is special concern about its lack of transparency in policing conflicts of interest [61]. The *NYT* reported that “Lisa Bero, chief scientist at the University of Colorado Center for Bioethics and Humanities, said [NASEM’s] longtime failure to disclose financial ties between committee members and industry placed the Academies in the ‘dark ages’ of research integrity.”^22^

Conflicts of Interest (COI) at the National Academies at all levels have been a concern for years [61]. NASEM, in addition to government support, receives a substantial amount of its funding from industries and organizations with vested interests. For example, from the sugar industry, the WK Kellogg Foundation is listed in the highest category of donors, giving more than $25 million. Other sugar industry and dental industry donors to NASEM include Coca-Cola, PepsiCo, Dr. Pepper Snapple, Mars International (candy), Tate & Lyle (sugar company), Hershey (candy), Proctor & Gamble (fluoride toothpaste), Colgate (fluoride toothpaste), and the California Dental Association [220].

With fluoride and fluoridation, the National Academies has a history of conflicts of interest dating back 70 years to its first reports on the subject in the early 1950s. Since then, at least ten reports discussing fluoride safety and effectiveness have been issued, the most recent in 2021 [14, 115, 212, 221–227]. Most have strongly endorsed the safety and/or effectiveness of fluoridation. Many have had committees heavily weighted with dentists and supporters of fluoridation. For example, the sugar-industry-funded Gerald Cox wrote a chapter supporting water fluoridation in the NRC 1952 report.

An NRC 1993 review of fluoride health effects followed the pattern of downplaying evidence of harm and had prominent fluoridation advocates on its panel [225]. But it also had a hidden and more egregious conflict of interest that only surfaced years later from lawsuits that supplied evidence for The Tobacco Industry Documents collection at UCSF. The chair of the NRC 1993 committee, Bernard Wagner MD, was secretly receiving a half million dollars a year from tobacco industry giant RJ Reynolds (RJR) [228, 229]. By the time of the NRC 1993 review, RJ Reynolds had merged with Nabisco, a processed foods conglomerate that included cookie, candy, and other sugary foods brands [230, 231]. Wagner’s activities included contacting medical journal editors to influence the acceptance or rejection of articles related to tobacco. Wagner also wrote and solicited editorials for a journal he edited, which promoted doubts about the evidence for cigarettes’ harmful health effects [232–234].^23^

It is unknown whether the National Academies was aware of Wagner’s substantial consulting contract with a tobacco and processed food company. No public acknowledgment of these conflicts was ever disclosed by the National Academies. In addition to substantial payments from RJR Nabisco, Wagner also had contacts with the processed food and chemical industry front-group ILSI, an organization that would play a continuing role in manipulating fluoride science as described below [238–241].

The NRC 1993 review committee that Wagner chaired was described by a committee member of a later NRC 2006 review as having “mostly researchers who were in support of fluoridation” [242]. The committee concluded the EPA’s recently raised regulatory standard of 4 mg/L fluoride in drinking water was sufficiently protective against any adverse health effects. Yet there was strong opposition to this less protective fluoride standard from the environmental group Natural Resources Defense Council (NRDC) and the union representing EPA’s own scientists who had reviewed the evidence on harm from fluoride [243–245].

In 2003 Wagner was initially appointed to the next NRC committee reviewing fluoride health effects, but soon afterwards he abruptly resigned, possibly as a result of complaints of conflicts of interest submitted to NRC for other committee members [212, 246, 247]. Wagner’s conflict of interest with the tobacco and processed food industry was not publicly revealed until years later, although it is possible the NRC was aware of it in 2003.

Despite Wagner’s removal from the NRC 2006 committee, the influence of the processed food and chemical industry continued. The chair of the NRC 2006 committee was Wagner’s colleague, John Doull, who also had numerous conflicts of interest with the processed food and chemical industries [248]. Doull and Wagner were both members of the industry-funded Flavor Extract Manufacturers Association (FEMA) committees that decide on GRAS (Generally Recognized As Safe) status of food additives, a system akin to the fox guarding the chicken house according to the US Government Accountability Office [249].^24^ Doull was also cited in the complaint letter to NRC as having a conflict because he was an advisory board member to Fred Stare’s ACSH, a group with a long record of promoting fluoridation [247]. Together with Doull, another NRC committee member, Charles Poole, was also cited as having a conflict because of membership on the ACSH advisory board [250]. Neither resigned from the NRC committee but within weeks both were no longer listed on the ACSH advisory board [251].

Conflicts of interest at NASEM concerning fluoride continue to the present day.^25^

### 2000s: Coca-Cola donates $1 million to pediatric dentistry association in 2003, gets policy changed from “[sugary drinks] a significant factor … for dental caries” to “not clear”

A more recent, and more blatant, example of the sugar industry continuing to influence dentistry occurred in 2003 when Coca-Cola donated $1 million to the small professional organization The American Academy of Pediatric Dentistry (AAPD) [256]. The public interest group Center for Science in the Public Interest (CSPI) described what happened [257]:

The academy became a laughing stock when the public (and its members) learned of the deal—imagine, an organization ostensibly concerned about children’s teeth taking money from arguably the world’s biggest producer of sugary foods. But the situation got worse when AAPD President David Curtis defended his group. He stated: “Scientific evidence is certainly not clear on the exact role that soft drinks play in terms of children's oral disease.” That was quite different from the group’s previous position: “frequent consumption of sugars in any beverage can be a significant factor in the child and adolescent diet that contributes to the initiation and progression of dental caries.”

What a difference a million dollars makes!

The AAPD, like most organizations of dentists, has a long-standing policy of promoting water fluoridation [258, 259]. Is that policy based on reliable scientific evidence, or might the hidden hand of the sugar industry be a factor?

#### The sugar industry wasn’t alone in using front groups in the early promotion of fluoridation

Although the newest uncovered evidence points squarely at the sugar industry as one of the first industries to manipulate science to promote fluoridation, other powerful industries and government interests were also playing important roles in influencing science, public opinion, and dentistry. The investigative journalist Chris Bryson spent ten years uncovering thousands of pages of documents revealing the role of fluoride-polluting industries like those that produce aluminum, steel, and chemicals [260]. The US military’s atomic weapons program collaborated too, because it relied heavily on the use of huge quantities of fluoride to enrich uranium to build atomic bombs. Bryson’s 2004 book *The Fluoride Deception* documents in detail how these industries played a major behind-the-scenes role in promoting fluoridation [46, 135].

The aluminum industry appears to have been the earliest to spin fluoride science, starting in 1931 almost immediately after fluoride was found to be the cause of dental mottling (now known as dental fluorosis). Alcoa’s internal investigations soon revealed dental fluorosis was appearing in children living near their polluting factories. But Bryson’s book was published before most of the sugar industry documents came to light. If they had been available, he might have included chapters on how the sugar and processed food industries were also manipulating the evidence to influence dentists, doctors, public health officials, and the public.

Taken together, the role of powerful commercial and government interests may answer the frequent question of newcomers to the issue of fluoridation: “If it is so bad, then why is it done?”.
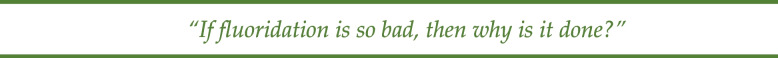


Many dentists and public health officials seem not to have been aware of the industry manipulations. From the earliest days, they hoped for a “magic bullet” against tooth decay, and many may have suspended their critical thinking. Fluoridation promoters continue to try to dismiss evidence of its risks and of its limited effectiveness, and vested interests continue to feed them biased science, as will be discussed next.

### 2020s: Manipulation of fluoride science continues to this day

The sugar industry along with the processed food, chemical, and pharmaceutical industries, has continued to try to manipulate the science to protect fluoridation from the rapidly emerging evidence demonstrating fluoride’s neurotoxicity. Numerous studies have now found reduced IQ in children exposed to levels of fluoride common, or only slightly higher, than in the US population [8–18]. In an apparent attempt to counteract this science, a group of 31 mostly German authors published a review of fluoride neurotoxicity in 2020 along with an update in 2021 and also a letter^26^ summarizing the review [20, 261, 262]. The authors all claim to be independent and declare they have no conflicts of interest. But a look into their history reveals that they are all members or closely associated with a German commission that reviews chemical and food risks but seems to have been co-opted by people connected to an industry front group, the International Life Sciences Institute (ILSI) [263]. ILSI was founded by a vice-president of Coca-Cola and has been funded by Coca-Cola along with a long list of major companies in the sugary foods, processed foods, infant formula, chemical, pesticide, oil, and pharmaceutical industries [264, 265]. Yudkin pointed out in 1972 that Coca-Cola was the largest user of sugar in the world [63] (p. 179).

In 2020, while a lawsuit filed by environmental groups against the US EPA's lax regulation of fluoridation was gaining traction, and as drafts of a National Toxicology Program systematic review on neurotoxicity were indicating that fluoride could lower children's IQ, this group of ostensibly independent German researchers published their own review [20, 266]. Its conclusion was the opposite of reviews by researchers with no industry ties and contrasted sharply with that of the US National Toxicology Program (NTP) [213, 267]. The Guth et al. review said the evidence was not sufficient to consider fluoride neurotoxic at common human exposure levels. A press release headline and plain-language summary accompanying the paper conclude in bold font “No cause for concern” [268, 269].

**Fig. 9:**
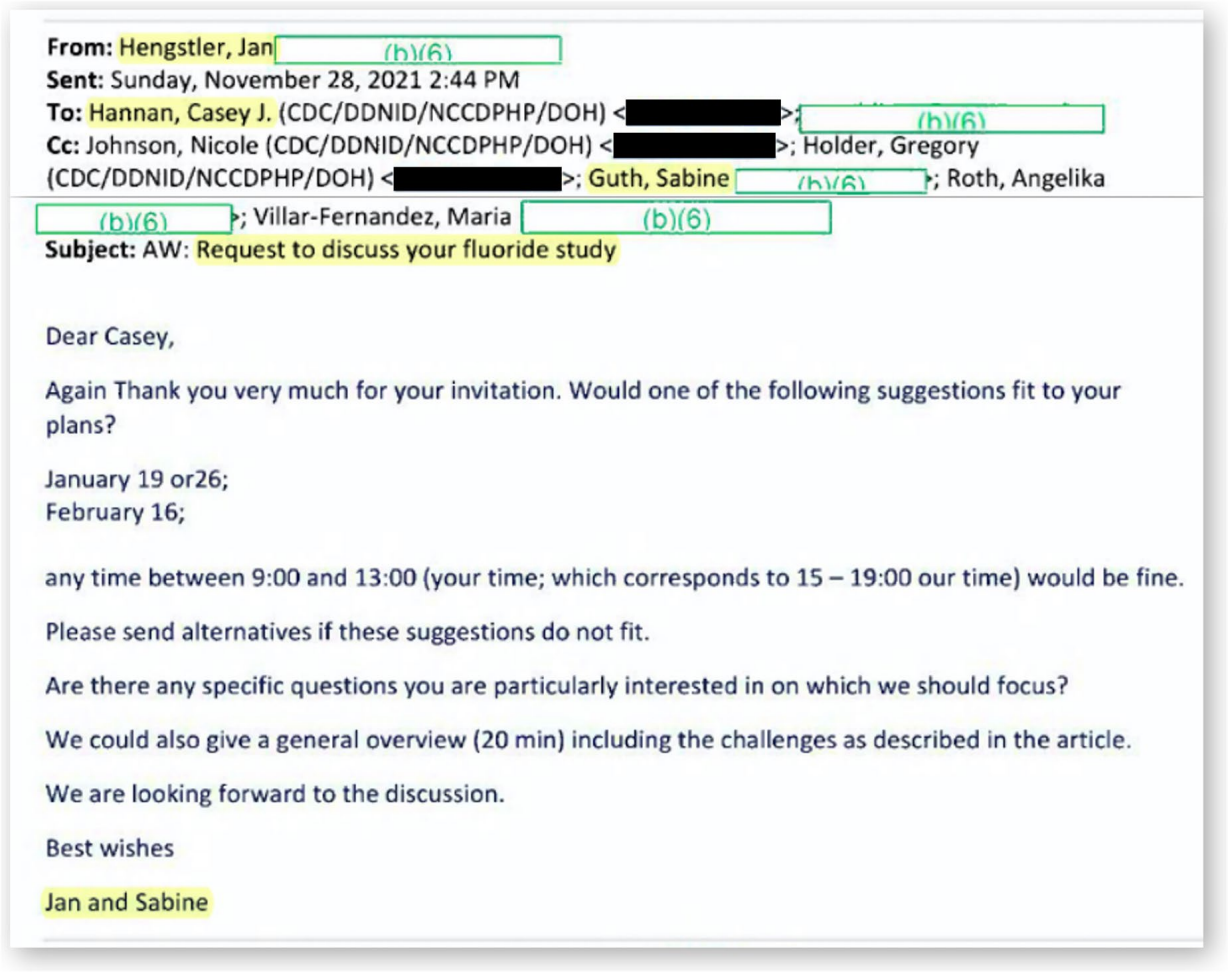
November 2021 email from Hengstler to CDC Oral Health Division Director Casey Hannan accepting an invitation for Zoom meeting to discuss the Guth et al. 2020 paper. Other emails show the meeting was delayed and actually took place in March 2022. Green redactions applied by CDC. Black redactions applied by author to email addresses. Yellow highlighting applied by author. Email obtained through Freedom of Information Act (FOIA) request to CDC and is in the public domain

**Fig. 10:**
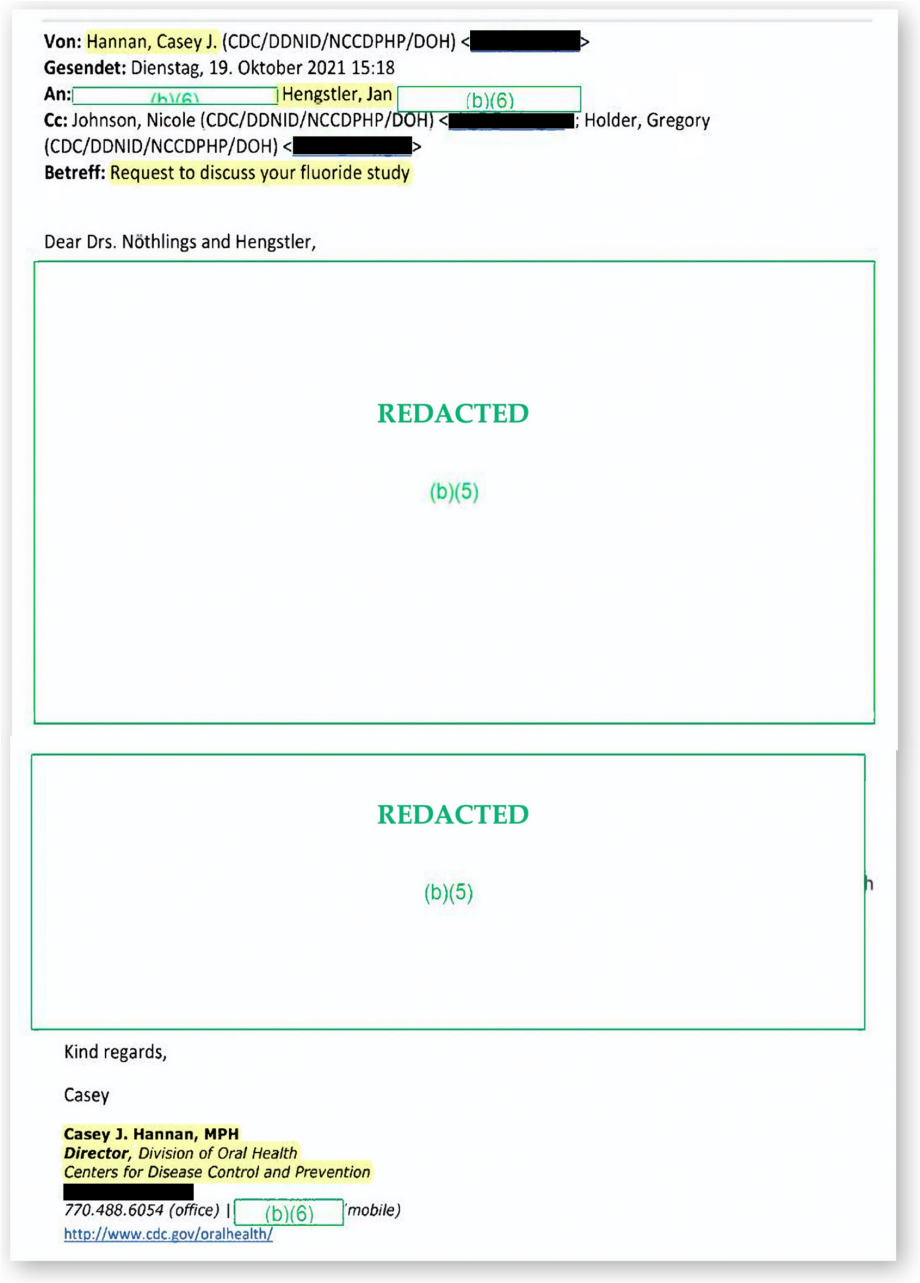
October 2021 email from CDC to Hengstler requesting meeting to discuss the Guth et al. 2020 paper. REDACTED by CDC in FOIA response. Green redactions applied by CDC. Black redactions applied by author to email addresses. Yellow highlighting applied by author. Email obtained through Freedom of Information Act (FOIA) request to CDC and is in the public domain

Fluoridation promoters have trumpeted the Guth et al. articles [270–272]. Freedom of Information Act (FOIA) requests also revealed that the Oral Health Division of the US CDC (Centers for Disease Control)–the federal agency most responsible for promoting fluoridation–arranged to privately meet with the German authors in an apparent effort to get assistance counteracting the NTP systematic review (Figs. 9 and 10). The documents released by the US CDC are heavily redacted, suggesting the CDC wished to hide the details of their communications with the authors of Guth et al.^27^

Not only were the authors of Guth et al. apparently asked to secretly help the CDC counteract NTP’s systematic review of fluoride neurotoxicity, they were also chosen to peer-review an update of a New Zealand government report on fluoridation that contains numerous errors and makes conclusions almost identical to those of Guth et al. [276, 277]. The New Zealand public health department is a strong proponent of fluoridation and has a history of issuing reports downplaying adverse health effects of fluoride [277–281].

#### Bias in the German SKLM commission review of fluoride neurotoxicity (Guth et al. 2020)

The Guth et al. articles are fundamentally flawed and biased. They repeat discredited claims put out by groups like the secretive Canadian Agency for Drugs and Technologies in Health (CADTH) report authors (who published anonymously and refused to release their names, and whose reports were subsequently withdrawn) and the industry-friendly Science Media Centre (SMC) of the United Kingdom [282–291]. For example, the Guth et al. articles claim prospective mother–offspring studies that found lowered IQ from fluoride–such as the NIH-funded cohort studies in Canada and Mexico [10, 11, 13]–did not adequately control for enough confounders. Yet they accounted for as many as 28 potential confounders, far more than the single “no effect” study Guth et al. used as a counterexample: the Broadbent et al. (2015) paper [292]. Furthermore, the NTP’s systematic review rated the NIH-funded cohort studies as high quality while the Broadbent 2015 study was rated low quality [293]. Tellingly, one reason for NTP’s low rating of the Broadbent 2015 study was its inadequate control of confounding [294]. Another reason Broadbent 2015 was given a low rating by NTP was that it was an ecological study, considered a weaker design, because it did not measure water fluoride exposure at the individual level.

The Guth et al. 2020 review identified 23 human epidemiological studies of the association between fluoride and developmental neurotoxicity and acknowledged that 21 of the 23 found higher fluoride exposures associated with lower intelligence. However, in their criticisms of the studies finding adverse associations, they make statements not based on, and contradictory to, what was reported in the original papers. Instead of citing the papers, they cite letters-to-the-editor by fluoridation defenders that criticized a 2012 and 2014 review of the older literature.^28^ The criticisms in those letters had been refuted by the reviews’ authors, and many were shown to be based on errors or misunderstandings [295]. For example, a letter authored by two dentists mistakenly claimed the difference in IQ between high and low fluoride groups in the review’s meta-analysis was only 0.4 IQ points, when it was actually 0.4 standard deviations of the IQ scale or 7 IQ points [296]. This same fundamental error that drastically understated the severity of the IQ loss was later repeated by other fluoridation defenders, even making it into a review of fluoridation safety sponsored by New Zealand’s Senior Science Advisor and the Royal Society of New Zealand [279, 297, 298]. Even more indicative of the biased nature of the Guth et al. review is how outdated it is. It omits the NTP (2019) review and the Grandjean (2019) updated review, updates that considered many additional high-quality studies published after 2012 [267, 299]. Guth et al. also use a straw-man tactic, focusing their attention on claimed weaknesses in some of the early studies but suggesting those weaknesses apply to the entire body of studies including higher-quality studies published after 2014.

Guth et al. employ recognized methods of “bending science” [31]. Many of their methods can be matched to the chapter titles in McGarity & Wagner’s book *Bending Science*:

“Chapter 3. shaping science, creating research to fit one’s needs.”

The Guth et al. review extols the Broadbent 2015 study while overlooking its serious weaknesses that can explain why it found “no effect” of fluoride on IQ. A major weakness was the lack of contrast in total fluoride exposure between the “fluoridated” and “non-fluoridated” subjects, thereby almost guaranteeing that little difference in IQ would be found between the groups [300]. Another limitation of Broadbent 2015 compared to the NIH-funded Bashash 2017 and Green 2019 studies was that it was ecological, without individual-level measures of fluoride exposure. The Broadbent 2015 study also lacked information on prenatal fluoride exposures, which other studies have found to be a critical exposure period [10]. The Broadbent 2015 paper itself was authored by dentists with a history of promoting fluoridation and reveals in its introduction an underlying political motivation to counter citizen efforts to stop fluoridation in New Zealand [292].

“Chapter 4. Hiding Science, Concealing Unwelcome Information.”

Guth et al. omitted mention of the Grandjean 2019 and NTP 2019 reviews, both of which concluded the scientific evidence was strong that fluoride was a developmental neurotoxin.

“Chapter 5. attacking science, turning reliable research into ‘junk’.”

For example, falsely stating the studies linking fluoride to lowered IQ “lack control of confounding factors such as … well-known neurotoxicants”, which ignores the fact that several of the higher quality studies accounted for lead, mercury, arsenic, manganese, and other neurotoxicants. Furthermore, reviews by Choi (2012), Grandjean (2019), and NTP (2020) concluded many of the studies that did not explicitly control for these neurotoxicants were unlikely to have suffered confounding because, in the specific settings where the studies took place, there was unlikely to be an association between the other neurotoxicants and fluoride [16, 213, 267]. Guth et al. revealed a double standard by falsely claiming the studies finding adverse effects on IQ did not adequately account for other neurotoxicants, while failing to mention that their favored Broadbent 2015 “no effect” study did not control for any neurotoxicants, despite a study location where lead and manganese exposures may have been higher in the non-fluoridated areas, potentially biasing the results away from a true adverse effect [300].

“Chapter 8. packaging science, assembling an expert group to advance a favored outcome.”

The German committee that wrote the Guth et al. review (SKLM commission) has been chaired for decades by the industry-friendly senior authors Hengstler and Eisenbrand as described below.

These examples from Guth et al. are tactics the sugar and allied interests have long used to manipulate the science. A recent analysis comparing five different industries, including the sugar industry, identified 28 specific science manipulation tactics [56]. The sugar industry engaged in most of them, and historical evidence suggests it may have pioneered many of them.

### Are the Guth paper authors truly independent?

Almost all the 31 co-authors of the Guth papers appear to be members of a single committee, the SKLM commission of the German Research Academy, which is charged with reviewing food safety [301]. A senior author, Jan Hengstler, is the chair of the commission. Another author, Gerhard Eisenbrand, is the most senior member and was the decades-long chair of SKLM before Hengstler took the reins [302]. The SKLM commission appears to have been a virtual fiefdom of Eisenbrand and has had unprecedented influence in evaluating and regulating chemicals found in foods, in Germany and the European Union (EU) [303, 304].

### Who are Eisenbrand, Hengstler, and their German SKLM commission that evaluates risks of chemicals in foods?

**Fig. 11:**
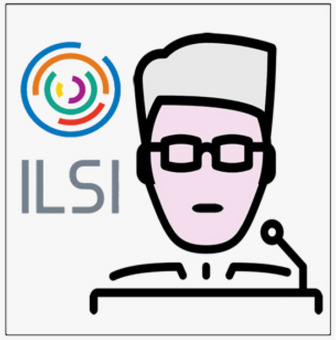
Graphic icon representing Gerhard Eisenbrand who is the science director of ILSI Europe, an industry front-group. He also has other industry conflicts of interest and was long-time chair of the German SKLM food safety committee, now chaired by Hengstler. (See photo of Eisenbrand presenting at an event for industry front-group ILSI [309]).


Eisenbrand (Fig. 11) has numerous conflicts of interest with industry, yet he often does not declare them. An exception was when he was required to do so as a member of a European Union committee in 2012. He revealed he has been a consultant or received funding from ten companies and industry associations, all food and pharma related. They include a European subsidiary of Colgate that makes fluoride toothpastes [305].^29^

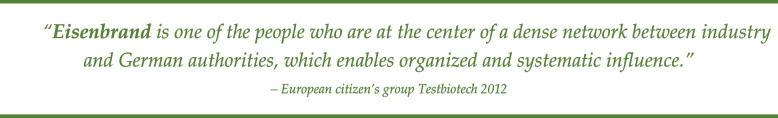


But Eisenbrand’s most egregious conflict comes from his long-time leadership of the European branch of the International Life Sciences Institute (ILSI), an industry front group. ILSI has been funded and controlled largely by the sugar, processed food, pharma, and chemical industries [264, 265]. Nowhere on ILSI websites or in their publicity are mentioned their deep connections to these companies which wish to hide their manipulation of science. A report by a European citizen’s watchdog group describes Eisenbrand’s central role in promoting industry interests on topics related to food and chemical safety [308] (Google translation of original German):

Overall, the picture emerges of an organized and at least partially covert influence exerted by industry in [Germany’s] central federal institutions that are concerned with risk assessment and research funding ….

› Gerhard Eisenbrand is the chair of a committee for the risk assessment of food products (SKLM) at the German Research Foundation (DFG), a member of the Committee for Genetically Modified Food and Feed at the German Federal Institute for Risk Assessment (BfR) and a member of the scientific board of scientific advisors at the BfR. At the same time, he is executive and scientific director of ILSI Europe.

Through his contacts with the DFG, BfR, BLL [“probably the most influential lobbying association for the food industry in Germany”] and ILSI, Eisenbrand is one of the people who are at the center of a dense network between industry and German authorities, which enables organized and systematic influence.

If you look at the BfR, DFG and EFSA [European Food Safety Authority] together, it is amazing how many people who are on the committees at the BfR, DFG or EFSA are in contact with ILSI at the same time

**Fig. 12:**
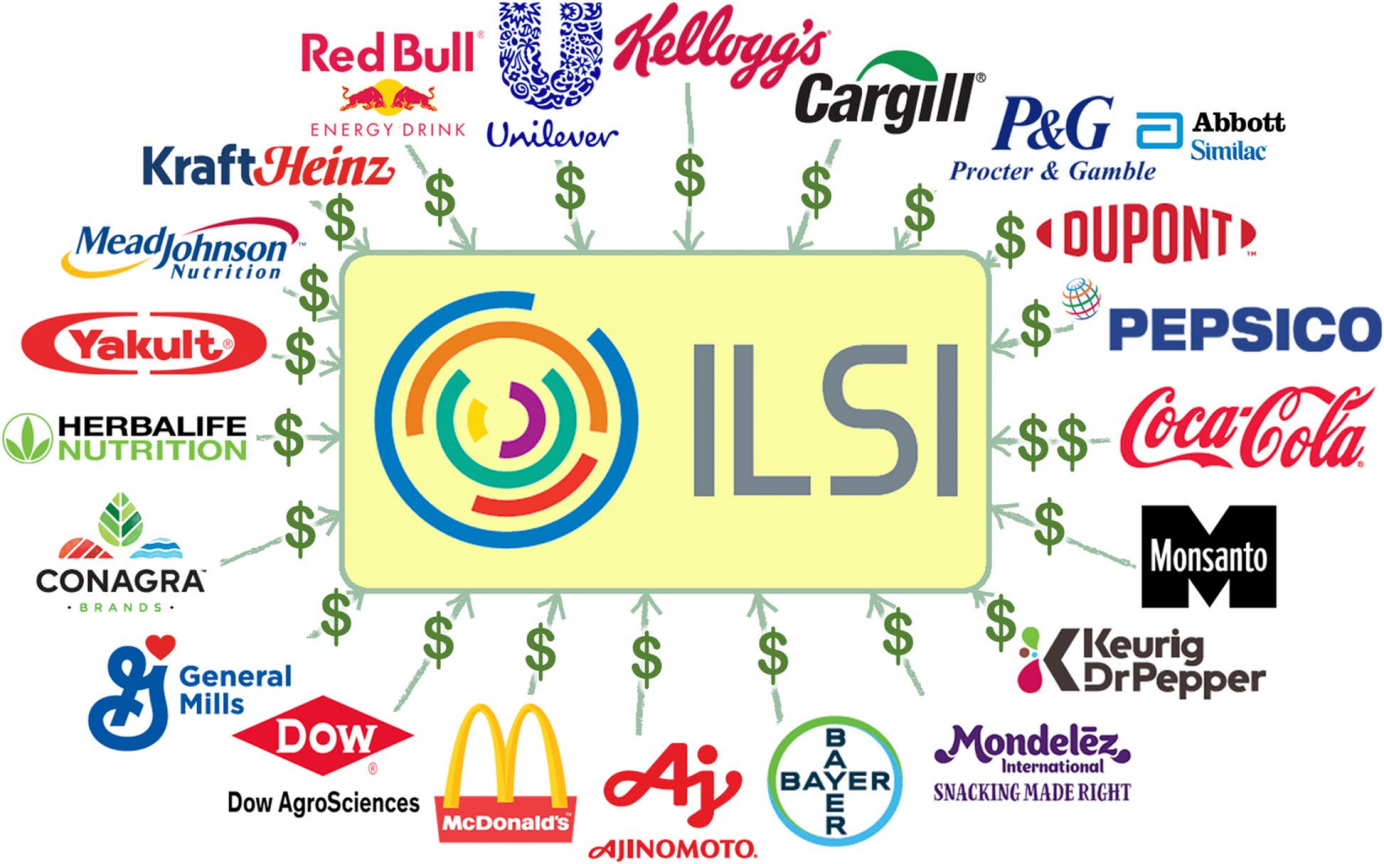
Some of the corporate funders of ILSI. Adapted and modified, with permission, from Corporate Accountability 2020 report “Partnership for an unhealthy planet” [311] (p. 5)

ILSI was founded in 1978 by Coca-Cola, Pepsi Cola, General Foods, Kraft (owned by the Philip Morris tobacco company), and Procter & Gamble. “ILSI grew very quickly into a powerful force, and began to also lobby for agriculture and genetic modification; pesticides and pharmaceuticals; confectionery; and eventually, even for such unhealthy consumable products such as cigarettes.” [264]. Major funders of ILSI also include Hershey, Mars, Kellogg’s, Monsanto, Dow, Syngenta, and many more (Fig. 12) [310].

ILSI has spawned numerous divisions including its Health & Environmental Science Institute (HESI), whose staff includes a former employee of Exponent, Inc., the same industry consultant company hired by EPA to supply expert witnesses to defend fluoridation in a recently concluded lawsuit brought by environmental groups [19, 266, 312–315].

At a congressional hearing in 2007, Dr. Jennifer Sass of the environmental group NRDC revealed troubling close connections between EPA and ILSI: “… a relationship that has demonstrably compromised the quality of EPA’s scientific inquiry is the Agency’s relationship with … ILSI.” EPA has even given ILSI millions of dollars in grants [316].

Consumer transparency advocacy group US Right to Know (USRTK) says plainly that ILSI “is a food industry lobby group” [265]. Researchers found “… a pattern of activity in which ILSI sought to exploit the credibility of scientists and academics to bolster industry positions and promote industry-devised content …” [58]. They also found “… ILSI promotes the interests of the food and agrichemical industries, including ILSI’s role in defending controversial food ingredients and suppressing views that are unfavorable to industry; … ILSI uses academics for their authority but allows industry hidden influence in their publications” [52].

A 2019 article in *The Guardian* newspaper highlighted the close connections between ILSI and sugary foods companies like Coca-Cola,^30^ as well as their connections to chemical companies like Monsanto, as revealed by internal emails [57, 320, 321]:

In a 2015 email copied to ILSI’s then director, Suzanne Harris, and executives from firms such as Coca-Cola and Monsanto, ILSI’s founder Alex Malaspina, a former Coca-Cola vice-president, complained bitterly about new US dietary guidelines for reducing sugar intake.

“These guidelines are a real disaster!” he wrote. “They could eventually affect us significantly in many ways; Soft drink taxations, modified school luncheon programs, a strong educational effort to educate children and adults to significanty [sic] limit their sugar intake,, [sic] curtail advertising of sugary foods and beverages and eventually a great pressure from CDC [the US Center for Disease Control and Prevention] and other agencies to force industry to start deducing [sic] drastically the sugar we add to processed foods and beverages.”

ILSI also has “cozy ties” with the CDC. Investigative reporter Carey Gillam wrote, “What is Going on at the CDC? Health Agency Ethics Need Scrutiny”. She reported “ILSI has a history of working to infiltrate public health organizations … with scientists, money and research to garner favor for industry products and strategies.” [322, 323].

An analysis of both public and internal industry documents by Mialon et al. (2021) found that ILSI has recently taken a step beyond manipulating nutrition science. ILSI is now trying to promote weak scientific integrity principles that ignore “the risks of accepting corporate funding”, resulting in standards that suit “industry’s interests rather than public health ones [55].”

Details of tactics used by ILSI to influence the science and public policy of obesity, a high-priority product defense issue for Coca-Cola, have been reported by Greenhalgh [324–327]. Her findings confirm descriptions by journalists and activist groups of an organization that promotes the interests of its corporate funders through ostensibly independent scientists. Some willingly collaborate because of shared viewpoints, while others are influenced by industry funding, which they do not always declare. Greenhalgh also describes ILSI’s co-optation of scientists and policymakers who may not fully appreciate that ILSI’s agenda is controlled by its industry funders [327].

In the Guth et al. reviews Eisenbrand declares no conflicts of interest. Also, no documentary evidence was found of Eisenbrand informing the other SKLM committee members and coauthors of the Guth et al. reviews of his potential conflicts of interest with ILSI and other corporate entities with financial interests related to fluoride. SKLM did not respond to requests for its meeting minutes. However, Eisenbrand has been a member and chair of similar German advisory committees on chemical risks that post their meeting minutes online (committees of the German Federal Institute for Risk Assesssment or BfR). BfR policy requires declarations and recording of potential conflicts of interest at the beginning of each meeting, but in no meetings did Eisenbrand declare his leadership role in ILSI as a potential conflict [328–331].

### Who is Hengstler, who has taken over as chair of the German SKLM commission from Eisenbrand?

**Fig. 13:**
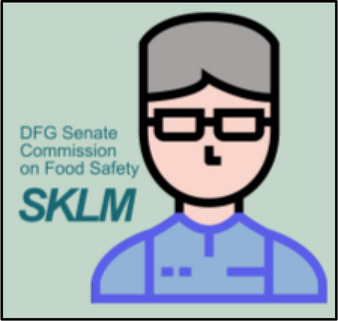
Graphic icon representing Jan Hengstler, current Chair of German SKLM food safety commission

Hengstler (Fig. 13) has a history of writing toxicological reviews of chemicals that reach conclusions favorable to food and chemical industries. He was one of eight industry-friendly toxicology journal editors who coordinated the simultaneous publication in 2020 of an editorial intended to influence European Union (EU) policy on the regulation of endocrine-disrupting chemicals (EDCs) [332].

In a counter-editorial a former Program Administrator of the NIEHS (National Institute of Environmental Health Sciences) component of NIH wrote with some sarcasm [333]:

The authors are … a group of [toxicologists] with no expertise in the endocrine-disrupting chemical scientific field, with undisclosed ties to the chemical industry, who have written an editorial so important it needed to be published in eight journals simultaneously.

But is the science accurate? Unfortunately, no.

French journalist Stéphane Horel found undeclared industry connections of many of the toxicologist editors [334]:

They call themselves"prominent"specialists; they are not.

They solemnly declare that they have no conflict of interest; however, half of them have collaborated with the chemical, pesticide, food or cosmetics industry over the last three years. Radically opposed to any regulation of endocrine--disrupting chemicals in Europe, 19 scientists have chosen to voice their opinions while an important decision-making process is underway in Brussels.

In 2013, Hengstler took part in a similar tactic of publishing identical editorials in multiple toxicology journals, again claiming EDCs were not a problem. That editorial was also timed to influence EU policy regulating EDCs. The outcry from the scientific community was equally great in 2013, with a counter-editorial issued by 48 editors of leading journals in the fields of endocrine and environmental health [335]. The counter-editorial says of the toxicologists’ editorial:

The editorial … capitalizes on uncertainty, as it seeks to foment doubt on the relevance of EDCs. Although the science behind EDC health effects is unequivocal, there continues to be unrelenting pressure from individuals and corporations with stakes in the status quo to keep doubt alive.

Phillipe Grandjean and David Ozonoff, co-editors of the journal *Environmental Health*, wrote their own counter-editorial, saying [54]:

The parallel editorials in these scientific journals are not about specific research findings, nor existing science-based public policy. Instead they are written with the sole purpose of influencing pending policy decisions of the European Commission.

Fluoride was identified by the US National Research Council in 2006 as an EDC because of its association with thyroid dysfunction which has been identified as a mechanism for fluoride’s adverse effects on the developing brain [17, 212].

In 2019, Hengstler may have invented a new tactic to influence science in what he called a “satirical” editorial that essentially ridicules research and researchers who report finding toxic effects of endocrine-disrupting chemicals at low doses [336, 337].

In 2013 Hengstler was on the European steering committee of a toxicology organization funded by chemical industries that organized a conference dominated by presenters from chemical and pharma companies including Dow, DuPont, Syngenta, ExxonMobil, Pfizer, and Unilever [338–340].

In 2011, Hengstler was the lead author of a report that exonerated BPA (bisphenol A) of harm. An investigative reporter uncovered several authors’ links to the BPA industry [341]:

Four authors of a new report concluding that bisphenol A is safe have ties to companies and groups that benefit from the controversial chemical.

The report was written by the Advisory Committee to the German Society for Toxicology ….

One of Hengstler’s first publications, from 1994, concluded that cigarette smoke may be protective against genetic damage, a finding that likely pleased the tobacco industry [342]. The tobacco industry seems to have financially rewarded Hengstler and his mentor Hermann Bolt with over $500,000 in research funding [343].

A German organization working on the issue of genetically modified organisms did a careful analysis of Hengstler’s industry ties and concluded [344]:

… analysis shows that the journal [*Archives of Toxicology*] has to be regarded as highly biased towards industry. The current main editors, Jan Hengstler and Hermann Bolt, … have current or past ties to industry. Hermann Bolt even conducted research financed by the tobacco industry, and the journal has a long history of involvement with the tobacco industry.

Bolt, co-editor with Hengstler of *Archives of Toxicology*, was the former director of the German occupational health institute IfADo. While receiving funding from the tobacco industry he helped suppress articles unfavorable to smoking and eased publication of favorable articles [344].

By 2004, Bolt and Hengstler had moved on from tobacco industry funding to chemical industry funding with a paper based on work paid for by the European Chemistry Industry Council (CEFIC). For years CEFIC has had the largest EU lobbying spending out of over 400 registered companies and organizations. CEFIC spent over $10 million in 2022 and had a staff of 89 lobbyists [345, 346]. The paper by Bolt, Hengstler, and two others, argued many carcinogens should be considered to have thresholds below which they are of no concern [347]. Finding “safe” threshold doses for toxic chemicals is a “holy grail” of industry [348].^31^

This brings us full circle back to the Guth 2020 and 2021 articles on fluoride neurotoxicity, which had Hengstler and Eisenbrand as senior authors and were published in Hengstler’s journal *Archives of Toxicology*. These articles are the latest in a long string of “bent science” on fluoride health effects, dating back to the 1930s with Gerald Cox of the industry-funded Mellon Institute.

## Discussion

The documentary record implicates the sugar industry in the manipulation of fluoride science. This manipulation exaggerated fluoride’s effectiveness against tooth decay, starting with Cox’s claim that prenatal fluoride was necessary, and downplayed the harmful side-effects of dental use of fluoride, including from water fluoridation. The sugar industry used the same science manipulation tactics as the tobacco industry later became known for, but did so at least a decade before the tobacco industry. Indeed, the tobacco industry seems to have adopted many of its tactics directly from the sugar industry via PR people and scientists who first worked for the sugar industry but later switched to the tobacco industry.

Another outstanding and possibly unique feature of the sugar industry’s campaign to promote and defend fluoride is that the sugar industry helped “enlist” dentists and their main trade organization, the American Dental Association, along with public health agencies, to be the public face of fluoride promotion. The documentary evidence suggests that without the sugar industry’s distortion of the scientific evidence, the dental profession might not have been as enthusiastic about the promotion of fluoride. Campaigns by other industries and companies on issues like smoking, asbestos, lead, dioxin, pesticides, other toxic chemicals, and climate change have not enjoyed the advantage of substantial support from healthcare providers, their professional organizations, and government public health officials.

This investigation benefitted greatly from access to a large body of primary documents from inside the sugar industry, many of which contain information the industry never intended to become public. Several of the archives have been digitized and are available online, some with excellent full-text search functions that greatly facilitated the investigation of specific research questions.

Although many sugar industry documents and records from people associated with the sugar industry were available, several of the archives only became public by chance so there may be additional documents that could potentially add more details or even alter the interpretation of events. However, sugar industry records from the 1930s to the 1960s seem relatively complete. They show a consistent strategy with fluoride whose interpretation seems unlikely to be significantly altered by any missing documents.

A more serious limitation arises with trying to obtain internal communications of the sugar industry and researchers who had conflicts of interest during more recent time periods. Some NGO groups have been able to obtain internal documents from the sugar and related industries using public records access laws like the Freedom of Information Act (FOIA), but the record is not nearly as complete as that available from earlier periods. For example, there are very few internal documents and emails available for the Guth et al. authors with hidden conflicts. Also, unlike the many lawsuits against tobacco and chemical companies, which have produced a rich source of internal industry documents, there have been few lawsuits against sugar companies or related industries. Internal records of Eisenbrand and other members of the German SKLM committee that might reveal links between ILSI or other corporate or institutional actors and the Guth et al. reviews of fluoride neurotoxicity are not available. Interviews with these people or others with direct knowledge, along with new public records requests, should be considered for future research.

Similarly, the role the National Academies may have played in allowing sugar industry conflicts of interest was difficult to assess because internal documents of the National Academies are not easily available. As a private organization, it is not subject to FOIA and it has a history of resistance to public disclosure of conflicts of interest [216, 219].

On balance, however, the limitations on the availability of relevant documents were not so great that they prevented uncovering evidence of a consistent sugar industry strategy to distort the science of fluoride’s effectiveness and safety.

## Conclusion

### Industry’s long history of manipulating science: from defending lead, to sugar, to cigarettes, to endocrine disrupting chemicals, to fluoridation

The Guth et al. articles claiming fluoride is not neurotoxic at levels commonly found in humans appear to be just the latest example in a long history of industry manipulation of fluoride science. Over the decades, the sugar and allied industries started by promoting fluoridation as the miracle solution to tooth decay, but are now shifting their efforts to try to defend it from the emerging science showing it may have been lowering children’s IQ all along, just like “low-level” childhood lead exposure. The lead industry used many of the same tactics to defend its product and to delay regulation [131, 353, 354]. The tobacco industry, contrary to popular belief, did not originate these types of tactics but got many of them from the sugar industry’s playbook by way of well-connected fluoridation promoters like nutritionist Fred Stare and chemist Robert Hocket.

When will the dentists and government public health officials who promote fluoridation realize they have been misled by powerful industries working behind the scenes from the very earliest days of fluoridation?

#### Dedication

This article is dedicated to the memory and good work of the late Dr. David Egilman, MD, a fearless fighter for scientific integrity against vested interests. He summarized what he believed was the root of the problem [355]:

While most of us have been taught to view scientific research as an unbiased source of knowledge that defies political or economic interests, in fact science plays a central role in corporate efforts to maximize profits.

Egilman also adapted a famous quote by Dr. Irving Selikoff, champion of workers exposed to asbestos, who stated, “statistics are people with the tears wiped away”. Egilman transformed this to: “corporations are human beings with empathy washed away”, focusing on what he saw as the root cause of the tears along with a side-swipe at the US Supreme Court’s complicity in granting corporations personhood.

## Supplementary Information

Supplementary Material 1.

Supplementary Material 2.

Supplementary Material 3.

## Data Availability

All documents used in this study are publicly available or included in this article. Upon reasonable request, the author will also share copies of all cited documents.

## Abbreviations

AAPD: American Academy of Pediatric Dentistry
ACSH: American Council on Science and Health
ADA: American Dental Association
BPA: Bisphenol A
Ca: Calcium
CADTH: Canadian Agency for Drugs and Technologies in Health
CDC: US Centers for Disease Control
CDC/OH: US Centers for Disease Control Oral Health Division
CEFIC: European Chemical Industry Council
COI: Conflict of interest
CSPI: Center for Science in the Public Interest
EDC: Endocrine disrupting chemical
EPA: US Environmental Protection Agency
FEMA: Flavor Extract Manufacturers Association
FOIA: Freedom of Information Act
GRAS: Generally Recognized As Safe
IfADo: German Institute of Occupational Health, Toxicology
ILSI: International Life Sciences Institute
IOM: Institute of Medicine
ISRF: International Sugar Research Foundation
JADA: Journal of the American Dental Association
MIT: Massachusetts Institute of Technology
NASEM: National Academies of Science, Engineering, and Medicine
NF: Nutrition Foundation
NGO: Non-governmental organization
NIDR: National Institute of Dental Research
NIEHS: US National Institue of Environmental Health Sciences
NIH: US National Institutes of Health
NRC: National Research Council
NRDC: Natural Resources Defense Council
NTP: US National Toxicology Program
NYT: New York Times
OSHA: Occupational Safety and Health Administration
P: Phosphorus
PHS: US Public Health Service
PR: Public relations
RCT: Randomized controlled trial
SKLM: German Committee on Food Chemical Safety
SRF: Sugar Research Foundation
TIRF: Tobacco Industry Research Foundation
TRF: Tobacco Research Foundation
UCSF: University of California at San Francisco
US: United States
USRTK: United States Right to Know

## Footnotes

1 Bernays later advised the New York City Public Health Commissioner Leona Baumgartner on how to overcome resistance to water fluoridation of New York City [30].

2 Based on an estimate of about $20,000 in lost lifetime earnings per -1 IQ point and a life expectancy of 70 years [35].

3 Keys was the first grant recipient of the Sugar Research Foundation (SRF), and was the keynote speaker at SRF’s inaugural celebration meeting in June 1944 [66].

4 An early promotion of fluoride and fluoridation by Stare was in a 1961 editorial published in the *Journal of the American Medical Association (JAMA)* on the occasion of Stare being awarded a nutrition prize by the Nutrition Foundation [77]. Unmentioned was that the Nutrition Foundation was funded almost exclusively by food industries, including sugar. Furthermore, Stare was the long-time editor-in-chief of the Nutrition Foundation’s sponsored journal *Nutrition Review*. Ironically, some of Stare’s earliest scientific work in the 1930s was on fluoride toxicity. He worked with a researcher studying the adverse effects of fluoride on thyroid function and metabolism and co-authored two papers on the topic in 1934 [78, 79].

5 The 1953 NRC report was co-authored by Harold Hodge who had secretly worked on fluoride toxicity for the Manhattan Project during WWII and had then gone on to be a leading scientist promoting fluoridation [46]. The title of the NRC report was “The Problem of Providing Optimum Fluoride Intake for Prevention of Dental Caries” and it not only advocated water fluoridation but also potentially adding fluoride to two dietary items: salt and sugar [81].

6 One example of the Nutrition Foundation directly involving itself in promoting fluoridation was its attempts to suppress the publication of a monograph that had identified serious problems in the original community fluoridation trials of the 1940s. Those trials have been cited up through the present to promote fluoridation. The monograph was by Australian dentist Philip Sutton and gave detailed critiques of the methods and conclusions of the seminal trials. Sutton obtained a copy of a letter to his US publisher from the Nutrition Foundation which tried to discourage publication, suggesting the publisher’s reputation would be tarnished if it published Sutton’s monograph [85]. The Nutrition Foundation’s close connections with Stare means it is difficult to know whether it was being used as a front-group for Stare’s activities or was independently working to defend fluoridation in its attempts to block the publication of Sutton’s monograph.

7 Arguably, the lead industry had already been using some of the methods of manipulating science starting in the 1920s to downplay the evidence that leaded gasoline and lead paint were neurotoxic risks to workers, the public, and children [131–134]. The Lead Industry Association (LIA) was formed in 1928 and would serve a similar product defense function as the SRF and tobacco industry research organizations. Already in the early 1920s the developers of leaded gasoline had used claims of scientific uncertainty, employed political pressure, and made outright falsehoods, to manipulate federal and state agencies to exonerate leaded gasoline of harmful effects on health. Some of the same people defending lead for industry would manipulate fluoride science too, with Robert Kehoe and Charles Kettering well-documented examples beginning in the 1930s as described by investigative journalist Chris Bryson [46, 135]. Bryson also found evidence that Kettering and the Alcoa aluminum company (which had worker and environmental liability from its high levels of fluoride emissions) met with both the American Dental Association (ADA) and the US Public Health Service (PHS) dental division in the early 1930s to encourage research and policies on fluoride that might help shift attention away from the growing evidence it was an occupational and pollution hazard and instead promote its dental benefits [46] (p. 40). In 1936 Charles Kettering became a member of an ADA committee on dental caries even though he had no background in dentistry. The committee published a report with citations to his industry fluoride research associates. Around the same time, there is evidence Kettering donated a substantial sum to the ADA [46] (p. 42–43). Kettering was also a member of an SRF-sponsored committee [136], forming one more link between the lead industry, the sugar industry, fluoride, and dentistry.

8 Earlier, from 1931–1935, Hockett had worked for the US Public Health Service (PHS) in the same office that was conducting an investigation of the relationship between fluoride, dental fluorosis, and caries [137]. Those studies were led by dentist Trendley Dean who would go on to be the most important person in the establishment and promotion of fluoridation in the US. Dean has been described as “the Father of Fluoridation” [138]. In a 1944 letter written by Hockett discussing ideas for controlling caries without limiting sugar consumption, he mentions his time at PHS: “When the Public Health Service first began its work on fluoride, I was a member of their staff in Washington so that I have watched the development of this subject with keen interest for a long time.” [139]. It is unknown whether Hockett’s early acquaintance with PHS fluoride research was a reason he was hired as SRF’s Scientific Director.

9 A succinct example of Hockett’s adherence to the tobacco industry’s strategy of casting doubt on the science is his response at a 1978 congressional hearing on smoking and health. Following an hour of evasive responses, Hockett finally answered a congressman’s questions about whether lung cancer was linked to smoking, and inserted the word “doubt” twice in the space of a single line [144] (p. 49):
*Congressman MAGUIRE. If people were to stop smoking, Doctor, do you think the rate of lung cancer would go down in this country, based on your best scientific estimates and your best tentative judgments?* *Dr. HOCKETT. I doubt it. I doubt it would go down very much.*

10 Goss was known as John Hill’s “right-hand man” and helped lead Hill & Knowlton’s public relations (PR) campaign for the tobacco industry. Goss eventually became president of Hill & Knowlton [150]. There are several letters from Goss to Hockett and other SRF officials discussing PR strategies and activities from 1943 and 1944 [146, 147, 151–154]. One example was a tactic to ghost-write a news release about collaborations between the SRF and the Massachusetts Institute of Technology (MIT) that went out under MIT’s name and letterhead. The release was mostly drafted by SRF and copies were reproduced in the New York office of Goss’s PR firm, then inserted into MIT letterhead envelopes that had been brought back by Goss from Boston. Finally, the filled and addressed envelopes were sent back to Boston to be mailed from there so that they would receive Boston postmarks to complete the charade that the press release had originated from MIT rather than SRF [145]. Unsurprisingly, the press release described SRF in glowing terms. It quotes, for example, the President of MIT, Arthur Compton, as saying: “The sugar industry is to be commended for its public service and vision for making possible this objective research. I feel sure it will be rewarded by results of great scientific value to the public.” [155].

11 TIRC documents reveal that Ancel Keys was being funded by the tobacco industry for work on smoking and heart disease [157]. A decade earlier Hockett was secretly funding Keys for work related to sugar and heart disease. The TIRC grant to Keys was to add smoking as a factor in his population studies of dietary fat and heart disease. But only in Japan, where Hockett noted “the Japanese have been very heavy cigarette smokers” yet “heart diseases are low”. Hockett made plain TIRC’s goal: “We hope this grant … might show that tobacco is not a likely complication in the development of heart disease.” [157]. Tobacco industry documents further reveal that Hockett, as associate science director of TIRC, also funded Stare to do tobacco research using methods Stare had developed in his nutrition research [157]. Stare’s grant was for injecting mice with an emulsion of tobacco tars to see if they developed cancer. None of them did, although many died soon after the injections before Stare found a way to use just an extract of tobacco tars rather than the full mix created by smoking [158–160].

12 According to The Center For Media and Democracy’s SourceWatch, Stare essentially laundered money for Seltzer from the tobacco industry: “Over a number of decades, more than $2 million in tobacco money passed through secret accounts and were channeled to Seltzer via Stare. The companies paid on a pro-rata basis according to their sales. The money then went into a secret ‘Special Account #4’ held by the Kansas City law firm Shook, Hardy & Bacon. Payments were then made as grants to the Department of Nutrition, where Stare took his cut, and passed the rest to Seltzer.” [162].

13 The arrangement in which Stare surreptitiously passed tobacco money to Seltzer (while taking a cut) lent the name “Harvard” as cover for Seltzer’s cigarette defense efforts. It lasted from 1963–1977, and netted Seltzer over $2 million. Archived financial statements of Stare’s Harvard Nutrition Department from its inception in 1942 until 1971 have recently been made available online in Harvard University archives [42]. The records for 1967, for example, show the Council for Tobacco Research “donating” $30,000 to the department, with $25,000 earmarked for Seltzer and $5,000 for the department [165] (p. 19). Stare used semantic tricks to deny he accepted any money from tobacco interests [162].

14 Proctor describes the link between sugar and tobacco: “This business of sugar in tobacco leaf is a fascinating one — and insufficiently appreciated outside the tobacco man’s labs. Sugar and tobacco have a long and incestuous history, and as one leading insider put it in the 1940s, ‘Were it not for sugar, the American blended cigarette and with it the tobacco industry of the United States would not have achieved such tremendous development as it did in the first half of this century.’ The American-blend cigarette … was in fact a candied-up contraption.” [166] (p. 33).

15 The consultant who wrote “Tobacco and Sugar” was Dr. Francis Weiss. SRF’s science director Hockett would again turn to Weiss in 1955 after Hockett had become associate science director of the Tobacco Industry Research Committee (TIRC), asking for assistance in counteracting recent studies linking smoking to lung cancer [172]. For an upcoming presentation to physicians, Hockett sought ways to cast doubt with examples demonstrating “correlation is not causation.” He described what has become a classic technique amongst sowers of scientific doubt: *In order to dramatize the dangers in drawing"cause and effect"conclusions from parallel trends in two developments, I would be glad to have some concrete examples in the form of charts. There must be many growth curves for environmental factors that would parallel roughly or closely the growth in incidence of lung cancer in the United States. I would like to find several that would appear to be at least as logically connected with the etiology of the disease as cigarette smoking, and also several which are almost certainly unrelated. Showing actual curves of these two kinds should be an effective way of emphasizing the need for caution in drawing conclusions as to what* *causes* *what*. *Some of the changes I can think of which have come about in the last thirty years are listed*. Examples from Hockett’s list reveal a fertile mind trying hard to counteract the already strong evidence that linked cigarettes to lung cancer:
*1. Use of diesel engines.* *3. Use of oleomargarine.* *5. Use of lead in gasoline.* *8. Hospitalization insurance.* *9. Use of electric razors.* *12. Routine chest X-rays.* *14. Income tax rates.* Hockett’s argument against the seminal Doll & Hill smoking and lung cancer studies ignores the fact that they were not time correlation studies but case–control studies [173]. Doll & Hill had already replied to previous attempts to dismiss their findings with the “correlation is not causation” argument [174]:
*It would seem, according to some of the critics of our conclusion that “tobacco-smoking is a factor in the production of carcinoma of the lung”, … that we ourselves based that conclusion in part–and even largely–upon the observation that over the last 50 years cigarette smoking and the recorded mortality from lung cancer have risen together. In fact our conclusion is based upon evidence that is entirely independent of that time correlation.* … *Evidence based on 1,465 lung cancer patients and 1,465 carefully matched controls does, we submit, carry some weight. In view, however, of the emphasis that has been placed, quite wrongly in our view, upon the correlation in time we might perhaps remind the reader briefly of the salient features of that survey.* … *To limit the possible variables, the design of our survey deliberately made them equal in sex, age, place of interview, and, largely, in hospital of admission. It will be seen that they have also revealed themselves to be remarkably alike in other characteristics – their social class and their occupations, their residence near a gas works, their heating in the home, their previous history of certain respiratory diseases. In relation to smoking they do not differ in their use of petrol lighters, of hand-rolled cigarettes, of different brands of cigarettes; they do not differ in the habit of inhaling. With these striking equalities–particularly in some aspects of smoking–it would seem to us to be unreasonable to argue that either our controls or our cases are biased, and therefore not comparable, or that the information given by the two groups is not equally reliable.* … *This evidence, we repeat, is quite independent of the time correlation.* Despite Doll & Hill’s clear rebuttal of the time correlation argument, Hockett is still hoping to use it in 1955, three years after it was debunked.

16 In recent years a campaign by the Swedish candy industry to influence dental science has been uncovered, which started in the late 1940s. The campaign was apparently independent of the US sugar industry’s campaign but had ethically troubling aspects that are reminiscent of some activities related to the promotion of fluoridation in the US [46]. For several years starting in 1948 an experiment funded largely by candy companies and carried out by government doctors and dentists was done on mental institution patients in the Vipeholm mental hospital in Sweden [177]. Sugar in various forms and amounts was given in the diets, and tooth decay was tracked. In some groups fluoride was also given to see if it counteracted the decay caused by the sugar. For unclear reasons, one group was given extremely high quantities of sugar in the form of specially formulated sticky toffees that were too large to swallow whole and so would guarantee long contact of the sticky sugar to the tooth and maximize cariogenicity. Unsurprisingly, many of the patients in this group showed rapid increases in tooth decay. But instead of stopping the experiment, it was continued until many had all their teeth decayed and most never received any treatment. This study is frequently cited in the dental literature as the best evidence that sugar causes tooth decay, a finding that was apparently in doubt by some dentists when the study was started and continues to be disputed by the sugar industry even today. But the Vipeholm study’s findings held a silver lining for the sugar industry. Another conclusion was that the frequency of sugar consumption was more important than the total amount as a risk factor for caries. To this day in Sweden, there is a policy encouraging children to have all the candy they want but reserved for only one day a week on Saturday [178, 179]. While this may have reduced caries, it did not reduce total sugar consumption. The sugar industry “had its cake and ate it too”. The leader of the dental experiment at Vipeholm, Dr. Bengt Gustafsson, was involved in another ethically troubling experiment in Sweden, more directly involving fluoridation. Gustafsson was an advisor and behind-the-scenes promoter of a secret trial of adding fluoride to the drinking water of half the city of Norrköping, starting in 1951, without telling the public or even the local dentists [180]. When people learned they were human guinea pigs, several years later, a local group started fighting to stop it. The opposition to fluoridation eventually led to a nationwide ban in Sweden and a rebuke from the Swedish Supreme Court for the secret experiment in Norrköping. The sugar industry was not directly involved in this unethical experiment, but through Gustafsson whose Vipeholm mental institution experiment was largely funded by the sugar industry, there was assistance and encouragement of the Norrköping experiment. Some of the very first fluoridation trials in the US were also done on mental institution patients, without their knowledge, but those experiments are rarely mentioned when fluoridation is promoted [181, 182] (US Congress 1954, p. 46–49). Another early fluoridation trial in the US was in the majority-Black city of Marshall, Texas [183]. That was also apparently done secretly without informing the population [184]. It never received any rebuke or backlash the way the Swedish study did and was used to promote fluoridation, although it was never mentioned that the fluoridated city had the largest proportion of Blacks of any city in Texas while the non-fluoridated comparison city had a much smaller proportion of Blacks. It was only revealed after the experiment that the residents were not informed their water was being fluoridated. At the time, some surveys had found that Blacks had less decay than Whites. Whether this played any role in choosing the mostly Black community to be the fluoridated one is unknown. In 1970 the sugar industry’s ISRF Scientific Advisory Board (which included Fred Stare) discussed their desire to replicate the Vipeholm study, with orphans given sticky candy, but in a fluoridated area. Presumably, the sugar industry hoped to demonstrate that with fluoridation even sticky candy would not increase the risk of caries. The same meeting also endorsed a suggestion by Fred Stare for a survey of sugar consumption trends in fluoridated US cities. The purpose of such a study was stated explicitly “If sugar consumption has stayed the same during this time, while dental caries has been reduced by 60%, this information can be useful in improving the image of sugar” [185].

17 The committee was the Council on Dental Health.

18 The ADA’s Hillenbrand being named to a committee of the Kellogg Foundation was just one of many “interlocking directorates” between the dentist groups, sugary foods manufacturers, The Nutrition Foundation, and even chemical company executives concerned about liability from their fluoride pollution. Charles Kettering, who had helped develop the fluorochlorocarbon Freon for DuPont and then the lead additive tetra-ethyl lead used in leaded gasoline (he became president of the Ethyl Company that marketed the lead additive), joined the ADA’s board of trustees in 1936. It is unclear why he had an interest in dentistry, but he had a clear interest in defending fluoride-polluting industries [46] (pp. 40–44). Kettering first met with representatives of the ADA and the US Public Health Service (PHS) when he was vice president of research at General Motors in April 1936. Another surprising member of a board of directors was the President of MIT, Karl Compton, who was named the chair of the trustees of the Nutrition Foundation when it was founded in 1941. The Nutrition Foundation was a food industry front-group and it eventually merged with ILSI, a current-day industry front-group. Compton was a physicist, not a nutritionist or health professional. Soon after joining the Nutrition Foundation board of trustees he was to play a key role helping manage the Manhattan Project. Compton also was apparently close to the SRF’s Hockett, because he was named by Hockett as one of his top three references when Hockett applied for the position of science director of the tobacco industry’s research foundation in 1954. The Nutrition Foundation was closely linked to the Harvard Nutrition Department through Fredrick Stare. Stare was the editor of the Nutrition Foundation’s journal and his own Harvard Nutrition Department was largely funded by the food industry, especially sugary and processed foods [192].

19 The “fluoride-skeptic” *JADA* editor that Hillenbrand replaced in 1944 was Pierce Anthony who had written editorials questioning the safety of fluoridation, including one that concluded: “Because of our anxiety to find some therapeutic procedure that will promote mass prevention of caries, the seeming potentialities of fluorine appear speculatively attractive, but, in the light of our present knowledge or lack of knowledge of the chemistry of the subject, the potentialities for harm far outweigh those for good.” [193]. Pierce Anthony, a dental authority skeptical of fluoridation, was outmaneuvered by fluoridation-promoting dentists cooperating with sugar interests. This was responsible, in part, for the American Dental Association (ADA) and the United States PHS becoming the primary proponents of fluoridation around the world, and encouraging other countries to embrace fluoridation. But interestingly, a small-scale counter-example was playing out on the other side of the earth, in Queensland, Australia, where fluoridation-skeptical dentists declined overtures by local sugar industry actors to cooperate in promoting fluoridation. This was the sole Australian territory to resist fluoridation for many decades after it had been adopted in the rest of Australia. An account of the history of fluoridation in Queensland says in the early 1950s “The sugar industry presented a potentially powerful liaison between government and industry on the issue of water fluoridation” [194]. A politician connected to the local sugar interests was “open to the concept of water fluoridation as a means of removing the pressure from the sugar industry in relation to dental caries”. But when local dental officials publicly expressed reservations about fluoridation, this upset the sugar interests [194]: *The Colonial Sugar Refining Company countered these opinions by citing unnamed dental experts in New South Wales [a different territory] who favoured fluoridation as an alternative to the elimination of dietary carbohydrates. Three days later, Mr R Muir (General Secretary of The Sugar Growers Council) launched an acrimonious public attack on the dental profession. Muir defended the nutritional value of sugar, questioned its relevance to caries and accused the dental profession of ‘singular failure to improve the dental health of Australians’. Former animosities between the sugar industry and the dental profession were ignited and Queensland’s fluoridation cause lost a potential ally.* The Queensland experience, counterpoised against what happened in the US, suggests the importance of sugar industry cooperation with dental interests to spread fluoridation.

20 Ironically, Fice Mork’s paternal grandfather, with whom he shared his given name, owned a cigar store and then a cigar manufacturing company [202–204]. Direct connections between the sugar and tobacco industries are discussed elsewhere in this article.

21 The SRF-funded symposium on fluoride and tooth decay held in October 1944 was widely publicized. In contrast, another symposium, on fluoride’s adverse health effects on workers, had been held in secret several months earlier in January 1944, also in New York City. The symposium on adverse health effects was by invitation only, mostly to medical directors of industries with fluoride liability risks from worker exposures and community air pollution. The funder of the symposium on adverse health effects was kept secret, similar to the symposium on fluoride dental benefits. The funder of the adverse health effects symposium was the Manhattan Project, the top-secret WWII era program to develop an atom bomb. The Manhattan Project even got the US Public Health Service to be the ostensible organizer of the symposium. The Manhattan Project harnessed massive resources from many industries and required large quantities of fluoride. The government and its corporate partners had serious concerns about fluoride’s adverse effects on the workers, and potential lawsuits from damage to farms and people living near factories emitting fluorides. Only one person presented at both symposiums, and that was dentist David Ast, who was promoting what he hoped would be the first-ever human trial of intentionally fluoridating drinking water [46] (p 81). To the dentists who attended the SRF’s open symposium Ast extolled the large dental benefits he expected from fluoridation. To the industry doctors and managers attending the Manhattan Project’s secret symposium, Ast suggested his human fluoridation trial could supply evidence that fluoride-polluting industries could use to counteract legal damage claims from workers or people living near the factories. The coincidence of the two symposiums, around the same time, one of which promoted fluoride and the other defended fluoride raises questions on whether there were any connections between the people behind the two symposiums. I could not find documents connecting the sugar industry campaign directly to that of the Manhattan Project or the fluoride-polluting industries. However, intriguingly, the SRF’s Robert Hockett had connections to three people who were at the highest levels of the Manhattan Project. They were Karl Compton, James Conant, and Vannevar Bush who were the presidents, respectively, of MIT, Harvard, and the Carnegie Institution and all held leadership positions as WWII presidential science advisors overseeing the Manhattan Project [209–211]. These three men were the first three personal references Hockett gave in the resume he sent when applying for the job at the tobacco research institute [140]. In addition, it was Conant who asked the US Public Health Service to be the front-group to sponsor the symposium that was secretly being organized and funded by the Manhattan Project [46] (p 80). Could Hockett have gotten the idea of secretly funding a fluoride symposium from his associate Conant? Even if there were no connections between the sugar industry and the Manhattan Project and the industries helping to build the atomic bomb, the two parallel campaigns to promote fluoridation may have had some common contacts via dentists and public health officials. Both the sugar industry and the polluting industries/Manhattan Project intentionally used dentists and public health officials as front people to mask the true motives each had for promoting fluoridation [4, 5, 46].

22 A week after the initial exposé the *NYT* had a follow-up story “National Academies Members Demand Answers About Sacklers’ Donations”. One of the seventy-five concerned National Academy members was quoted: “The academy was looking like it had been morally asleep for the last 30 years” [219].

23 In a letter Wagner sent to his contact, the vice president of research at RJ Reynolds (RJR), he boasted of being named to the National Academies permanent Committee on Toxicology and the chairmanship of its fluoride health effects committee [233]. Wagner told another RJR scientist that he would try to get him appointed to a National Academies panel on carcinogenesis [235]. Wagner also arranged for several scientific conferences that would allow tobacco industry employees to present with the explicit arrangement that RJR would, in return, “contribute” generous funding for each conference, such as “a minimum contribution of $35,000” [236]. Wagner led an attack on the most junior (and only female) member of a research team that had published a paper in the *Journal of the American Medical Association* finding that 90% of 6-year-olds recognized the Joe Camel logo as representing cigarettes, the same rate they recognized the Mickey Mouse ears logo as representing the Disney Channel. Showing just how far Wagner was willing to go to defend tobacco interests, he sent a letter to the young research assistant essentially threatening her with accusations of scientific misconduct and demanding that she contact him and answer criticisms about the study’s methods, as well as provide all the study’s data [237].

24 For more on other conflicts of interest involving FEMA and current sugar-industry-connected individuals, see footnote 29.

25 NASEM was contracted to peer-review the National Toxicology Program’s (NTP) systematic review of fluoride neurotoxicity in 2020 and 2021 [14, 227]. One of the committee members had publicly attacked the first English-language article finding evidence that fluoride was neurotoxic [252, 253]. The criticism of the study along with personal aspersions cast against the study’s lead author, Phyllis Mullenix, were made in a newspaper article about a debate on fluoridation in Las Vegas, Nevada. The Mullenix study was seen as a major threat to fluoridation policy and its publication led to Mullenix being fired from her job as head of the toxicology division at the Harvard-affiliated Forsyth Dental Institute [254]. No errors were ever found in the Mullenix study and Mullenix won a lawsuit against Harvard over her wrongful dismissal. Although NASEM was notified of the apparent conflict of interest of the committee member who had attacked Mullenix and her work [255], the committee member was not removed and did not withdraw from the NASEM committee, unlike conflicted Bernard Wagner from an earlier National Academies committee on fluoride toxicity.

26 The letter-to-the-editor, by Seddek & Ghallab, briefly summarizes the Guth 2020 article but does not add any commentary [261]. Ghallab does not disclose that he is employed in the institute that is directed by Jan Hengstler, the senior corresponding author of the Guth articles and the editor of the journal that published the Guth articles and the letter. Neither Seddek nor Ghallab had ever previously published on fluoride or neurotoxicity. The letter appears to be a tactic to increase the scientific “footprint” of the Guth paper using a form of redundant publication and self-citation. This is reminiscent of the overt duplicate publication Hengstler has engaged in twice with identical editorials defending food chemicals published simultaneously in multiple toxicology journals. Most of the co-author editors of those journals had undisclosed links to food, pharma, and chemical industries. Hengstler’s history of duplicate publication and undisclosed conflicts of interest is described in more detail later in this article.

27 Among thousands of pages of documents obtained through FOIA, no records were found of the CDC Oral Health Division reaching out to authors of any of several published reviews that concluded there was an association between fluoride and reduced IQ in children, nor to any authors of the dozens of primary studies finding fluoride neurotoxicity. The FOIA documents instead reveal sustained efforts by CDC to alter and even block the NTP report from publication [273–275].

28 Guth et al. repeatedly cite letters-to-the-editor by Feldman 2014; Gelinas and Allukian 2014; Sabour and Ghorbani 2013; and Sutton et al. 2015 [20].

29 A more recent conflict of interest that was not acknowledged in Eisenbrand’s declaration for the EU was his membership in the Flavor and Extract Manufacturers Association (FEMA) committee on food additives Generally Regarded as Safe (GRAS) [306]. A study by Neltner et al. (2013) found widespread conflicts of interest in GRAS determinations [60]: “Between 1997 and 2012, financial conflicts of interest were ubiquitous in determinations that an additive to food was GRAS. The lack of independent review in GRAS determinations raises concerns about the integrity of the process and whether it ensures the safety of the food supply”. Commentary by Marion Nestle on the Netler study was less restrained: “An astonishing 100% of the members of 290 expert panels included in their review worked directly or indirectly for the companies that manufactured the additive in question. Even more alarming, the experts on these panels form a tight professional cadre. … The scientific substantiation used by manufacturers to support GRAS status is highly conflicted.” [59]. A US Government Accountability Office report concluded there is little government oversight of GRAS determinations which corporations are allowed to make entirely on their own [249]. The GRAS system is an egregious case of “the fox guarding the chicken coop”. FEMA’s GRAS determinations have also been described as a “black box” [307].

30 ILSI also has close connections with the successor organization of the SRF, currently called The Sugar Association, Inc. The president of the Sugar Association, Courtney Gaine, worked for ILSI before moving to The Sugar Association [317], and before that worked at a PR firm specializing in advising the food industry [318]. All of the academic papers she published on nutrition topics up until she started work with the Sugar Association were funded by food industries, including the National Cattleman’s Beef Association, The Egg Board, and the Gatorade company. It may not be surprising that virtually all of these scientific papers reached conclusions favorable to the industry funders [319].

31 Bolt and Hengstler have another apparent conflict of interest as the leaders in the toxicology section of the German Leibniz Research Centre for Working Environment and Human Factors (IfADo) [301, 349, 350]. According to a presentation given by Bolt about occupational diseases from toxic chemical exposures, in Germany, “Only those diseases which have been officially recognized by the Federal Government can be compensated”. “The Federal Government is free to exclude … or to place specific restrictions … to certain occupations, limit dose for cumulative exposure etc. The risk factor must be certain. A statistical association between an occupation and a disease is not sufficient. … The eligible group must be exposed to the risk factor to a higher degree than the rest of the population. [and] this higher exposure should lead to a higher risk of illness, in general doubling the relative risk or odds ratio!” [351, 352]. The list of officially recognized occupational diseases and the doses at which they are compensated is determined by scientific advice from groups such as the IfADo that has been led by Bolt and Hengstler. Thus, when Bolt and Hengstler publish reviews concluding there is uncertainty about whether a chemical causes a disease, or that a higher dose is required to establish compensation, then the German government and chemical industry will save money because they will not have to compensate as many workers. Slides number 30 through 33 in Bolt’s presentation give information on the number of individual worker compensation cases reviewed by IfADo in recent years, including by Bolt personally, and the number that were judged compensable by IfADo. This suggests IfADo not only has input into the federal occupational disease list and minimum exposures considered compensable but can also rule on specific cases. Thus, Bolt and Hengstler appear to have additional conflicts of interest when they publish papers or editorials that conclude there is uncertainty about a hazard, or that there is a higher dose threshold than what independent researchers may have concluded. Neither Bolt nor Hengstler have ever acknowledged these conflicts of interest with German worker compensation laws. Their situation is akin to that of a company doctor with the authority to determine whether an employee has been harmed by toxic chemicals in the workplace.
