# Supplementary material for: The sugar industry’s efforts to manipulate research on fluoride effectiveness and toxicity: a ninety-year history
Source: Environ Health. 2025 Sep 29;24:62. doi: 10.1186/s12940-025-01154-x (PMC12477810; doi:10.1186/s12940-025-01154-x)
Supplement: Supplementary file 1 — Supplementary Material 1. [file 12940_2025_1154_MOESM1_ESM.pdf]

Additional File 1

For:
The sugar industry's efforts to manipulate research on fluoride effectiveness and toxicity: A ninety-year history
Christopher Neurath

Figure S1 Timeline of sugar industry manipulation of fluoride science

Figure S2 Brief summaries of mentioned people and organizations

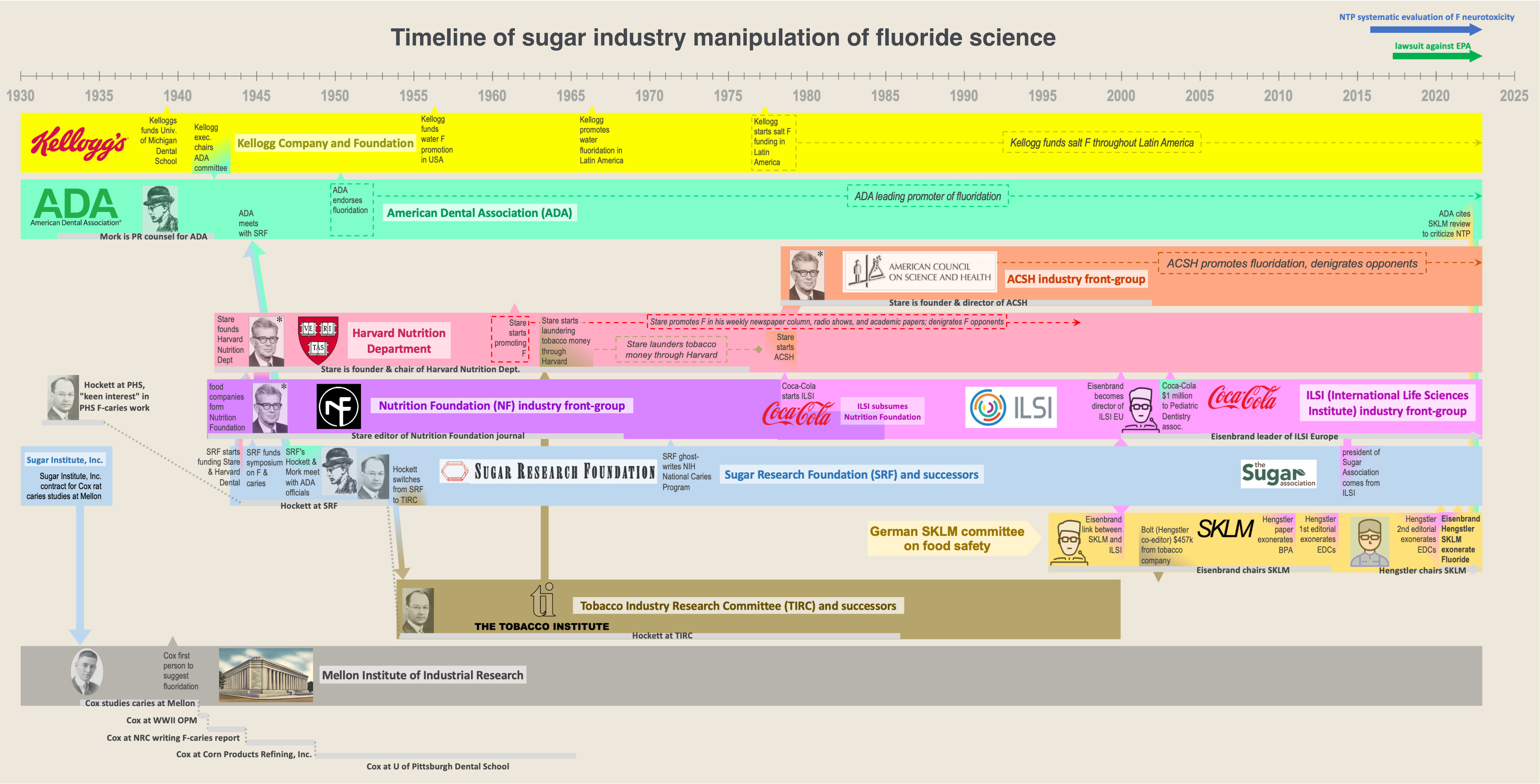

Fig. S1 Timeline of organizations, people, and events involved in manipulating fluoride science. Links between organizations indicated by arrows and blended colors.

* Photo of Frederick Stare from Hegsted DM. 2004. Fredrick John Stare (1910-2002). *Journal of Nutrition*. 134(5):1007-1009. https://doi.org/10.1093/jn/134.5.1007. Used with permission of Elsevier, https://www.sciencedirect.com/journal/the-journal-of-nutrition. The rights to this image are excluded from the Creative Commons CC-BY license.

Fig. S2 Brief summaries of mentioned people and organizations.

Major Actors

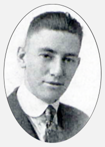

Gerald J Cox. From early 1930s, chemist at industrial research contractor The Mellon Institute who studied tooth decay in rats for the sugar industry, and concluded fluoride was the “magic bullet” against tooth decay.

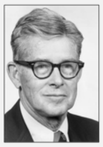

Photo credit *

Fredrick (Fred) Stare. From early 1940s, founder and long-time leader of Harvard Nutrition Department, funded largely by sugar companies and food industry. Early public promoter of fluoridation and defender of junk food, pesticides, and chemical additives in foods. Founded ACSH, an industry front-group that even more vociferously defended junk food, pesticides, and chemical additives in foods. Secretly laundered tobacco industry funding for a promoter of tobacco industry defenses who was staff at Harvard Nutrition Department. Image source: Hegsted (2004)*.

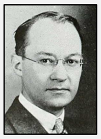

Robert Hockett. From 1943, science director of Sugar Research Foundation (SRF), and then switched to Tobacco Industry Research Committee in 1954 and continued there while maintaining cooperation with sugar industry defenders, including Fred Stare.

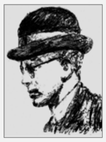

Fice Mork. Public Relations counsel for American Dental Association (ADA) in 1930s and 1940s and then SRF in 1940s. He advised sugar industry that fluoride was the solution to the problem of tooth decay caused by sugar and led some of the earliest campaigns to promote fluoridation.

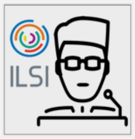

Gerhard Eisenbrand. From 1970s, German toxicologist and long-time chair of German SKLM committee which advises on food chemical safety while he was simultaneously the European head of the food and chemical industry front-group ILSI (see below). Consulted for food and pharma industry but usually failed to declare these conflicts of interest. Senior author of recent review of fluoride neurotoxicity that dismissed evidence of hazard.

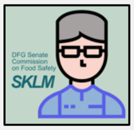

Jan Hengstler. From 1980s, German toxicologist and successor of Eisenbrand and current chair of SKLM committee on food chemical safety. Editor of industry-friendly toxicology journal and author of papers and editorials dismissing evidence of harm from food chemicals such as Bisphenol A (BPA) and EDCs. Senior corresponding author of recent review of fluoride neurotoxicity that dismissed evidence of hazard.

Other Actors

Emory Morris, dentist and long-time Kellogg’s Company executive; worked with American Dental Association.

David Ast, dentist, leader of one of first fluoridation trials.

Harold Hodge, toxicologist of Manhattan Project.

Arthur Compton, President of MIT and science director of Manhattan Project. Founding president of Nutrition Foundation and associate of Hockett. Helped arrange a secret 1944 symposium on occupational health effects of fluoride for the Manhattan Project.

James Conant, chemist, President of Harvard and oversaw Manhattan Project.

* Photo of Frederick Stare from Hegsted DM. 2004. Fredrick John Stare (1910-2002). *Journal of Nutrition*. 134(5):1007-1009. https://doi.org/10.1093/jn/134.5.1007. Used with permission of Elsevier, https://www.sciencedirect.com/journal/the-journal-of-nutrition. The rights to this image are excluded from the Creative Commons CC-BY license.

Chris Bryson, investigative journalist and author of *The Fluoride Deception*.

John Yudkin, nutritionist who investigated sugar's adverse health effects.

Ancel Keys, nutritionist funded by sugar industry. Criticized evidence that sugar played a role in cardiovascular disease and was main promoter of alternative theory that dietary fat was the cause of cardiovascular disease.

David Michaels, former director of the Occupational Safety and Health Administration (OSHA) and author of *Doubt is Their Product: How Industry's Assault on Science Threatens Your Health*.

Cristin Kearns, dentist and researcher; uncovered evidence of sugar industry manipulation of science.

Philippe Hujoel, dentist and researcher; uncovered evidence of sugar industry manipulation of American Dental Association.

Bernard Wagner MD, highly paid undisclosed consultant to RJR Nabisco Company and chair of National Research Council (NRC) 1993 committee on fluoride toxicity.

Major Organizations

Sugar Research Foundation (SRF), successor to Sugar Institute Inc, and succeeded by International Sugar Research Foundation (ISRF) and Sugar Association. From the 1930s sugar industry science and PR organizations set up to promote sugar use and defend sugar from threats of harming health.

Hill & Knowlton, Public Relations (PR) firm. PR advisors to SRF and later the tobacco industry's equivalent TIRC. Helped create methods of manipulating science to defend harmful products.

Nutrition Foundation and its journal *Nutrition Reviews;* merged with ILSI around 1980. Founded in 1941 and funded by food industry. Fred Stare was long-time editor of *Nutrition Reviews*.

American Dental Association (ADA). Since the 1950s the leading private organization promoting fluoridation in the United States. Professional and lobbying organization for dentists.

US Public Health Service (PHS). Since the 1940s the world's leading promoter of water fluoridation, including through its divisions CDC Oral Health, National Institute of Dental and Craniofacial Research (NIDCR), and the Surgeon General's office.

Harvard Nutrition Department. Founded and led by Fred Stare from 1942-1970s. Funded largely by sugar, processed food, chemical and agribusiness industries. Provided academic cover for industry-friendly research related to food and diet. Fred Stare used Harvard name to lend legitimacy in his promotion of fluoridation.

American Council on Science and Health (ACSH). Founded and led by Fred Stare. An industry front-group that vociferously defends junk food, pesticides, and chemical additives in foods. Long history of defending and promoting fluoridation.

Kellogg's Company and WK Kellogg Foundation. Makers of sugary cereals and promoters of fluoridation since 1940s. Close ties with ADA and major funder of dental schools in the US and fluoridation projects throughout Latin America.

Tobacco Industry Research Committee (TIRC) and successor Tobacco Research Foundation (TRF). Founded in 1953 to defend industry from threats of health harm. Ties to sugar industry through Hockett who had been science director of the similar SRF for the sugar industry and brought his skills at defending harmful products to the tobacco industry when he joined TIRC.

Coca-Cola Company (joined by other major sugary foods, processed foods, chemical, and pharma companies). Founded and major funder of ILSI. Donations to dental and nutrition groups to influence their policies on sugary beverages.

	**International Life Science Institute (ILSI).** Industry front-group founded and funded by Coca-Cola Company and other sugary foods, processed foods, pharma, and chemical companies. Merged with Nutrition Foundation around 1980.
	**SKLM German committee on food safety**. Chaired for many years by Eisenbrand and then by Hengstler, both industry-friendly toxicologists.
	**IfADo German institute of occupational health, toxicology**. Currently located at Leibniz University. Led by Jan Hengstler and formerly led by Hengstler's mentor Herman Bolt. IfADo plays major role in determining German worker compensation rules for toxic exposures. Hengstler and Bolt are co-editors of industry-friendly toxicology journal *Archives of Toxicology* and previously received major funding from tobacco industry. They have also received funding from chemical industries. Similar to Fred Stare and the Harvard Nutrition Department, IfADo provides academic cover for Hengstler. Hengstler is senior corresponding author of recent review that exonerates fluoride of neurotoxicity.
	**National Academies of Science, Engineering, and Medicine (NASEM); divisions include National Research Council (NRC) and Institute of Medicine (IOM)**. Weak conflict of interest policies and lack of transparency allowed committee appointees with substantial financial ties to tobacco, processed foods, and chemical industries to write reports or chair committees on fluoride. Also allowed committees on fluoride to be dominated by dentists with record of strongly promoting fluoridation.
	**Other Organizations**
	**Vipeholm Mental Hospital,** Sweden. Site of unethical experiments on patients with sugar-industry-funded studies on sugar, fluoride, and tooth decay.
	**Alcoa aluminum company**. Major fluoride polluter that was involved early on in behind-the-scenes promotion of fluoridation in an effort to distract people from the harm its own fluoride pollution was causing to workers and nearby residents of its factories.
	**European Chemistry Industry Council (CEFIC).** Largest lobbying organization in the European Union. Funded Hengstler and Bolt review that promoted chemical industry positions on carcinogens.
	**Exponent, Inc**. Industry consultants who specialize in product defense, often for chemical industry. US EPA (Environmental Protection Agency) hired them to help it defend EPA's lack of regulation of fluoridation in a lawsuit by environmental groups. Lawsuit is specifically to require EPA to regulate fluoridation to prevent risk of neurotoxic harm. EPA lost lawsuit in September 2024.
