# Supplementary material for: The sugar industry’s efforts to manipulate research on fluoride effectiveness and toxicity: a ninety-year history
Source: Environ Health. 2025 Sep 29;24:62. doi: 10.1186/s12940-025-01154-x (PMC12477810; doi:10.1186/s12940-025-01154-x)
Supplement: Supplementary file 2 — Supplementary Material 2. [file 12940_2025_1154_MOESM2_ESM.pdf]

# Additional File 2

For:
**The sugar industry's efforts to manipulate research on fluoride effectiveness and toxicity: A ninety-year history**
Christopher Neurath

Extract from transcript of deposition of Casey Hannan, Director of CDC Oral Health Division, in the legal case Food & Water Watch et al vs Environmental Protection Agency; Civil Action 17-cv-2162-EMC; in the United States District Court for the Northern District of California at San Francisco; pages 1 and 210-217. November 6, 2018.

IN THE UNITED STATES DISTRICT COURT
FOR THE NORTHERN DISTRICT OF CALIFORNIA
AT SAN FRANCISCO

| | |
|---|---|
| FOOD & WATER WATCH, et al., | |
| Plaintiffs, | CIVIL ACTION |
| vs. | FILE NO. |
| U.S. ENVIRONMENTAL PROTECTION AGENCY, et al., | 17-cv-2162-EMC |
| Defendants. | ORIGINAL |

VIDEOTAPE RULE 30(b)(6)DEPOSITION OF
CENTERS FOR DISEASE CONTROL & PREVENTION
THROUGH
CASEY HANNAN

Tuesday, November 6, 2018

10:15 a.m.

1600 Clifton Road
Building 21, Suite 10000
Atlanta, Georgia

Linda C. Ruggeri, CCR-A-261

**language of what it says here, right?**

MR. DO: Don't answer that question.

**Q. (By Mr. Connett) Based on the statement that CDC has provided, the systemic benefit is limited to the period when teeth are developing, right?**

A. That appears what CDC is saying here.

**Q. Okay. So what studies does CDC base the statement that fluoride also has a systemic effect?**

A. At this point I am not able to point to the exact studies that forms the basis of that position.

**Q. Okay. Can you provide me any explanation as to the basis for CDC's statement on this?**

A. Any explanation?

**Q. Yes. Any reference to any information, anything that I could go to to assess the foundation for CDC's position on this?**

A. At this point I wouldn't be able to provide that to you.

**Q. Okay. Now I'm going to turn and discuss Topic 12. Topic 12 pertains to CDC's position, if any, on whether prenatal exposure to fluoridated water provides a benefit to teeth and the basis for said position.**

A. We're on Topic 12, is that what you said?

**Q. Yes.**

A. CDC's position, if any -- okay.

**Q. And you're prepared today to discuss this topic, correct?**

A. Yes.

**Q. Okay. So earlier today you mentioned that one of the documents you reviewed was a 2001 report by CDC, correct?**

A. Uh-huh; correct.

**Q. I will go ahead and mark this, what I believe the document is that you were referring to, as Exhibit 53.**

(Exhibit 53 was marked for identification.)

**Q. (By Mr. Connett) And this document here is titled "Recommendations for Using Fluoride to Prevent and Control Dental Caries in the United States." It's published in MMWR, and the date is August 17th, 2001. Is this the document that you were referring to earlier?**

A. Yes.

**Q. So you reviewed this in preparation for today's deposition?**

A. I did.

**Q. Okay. So if you can turn to Page 16, if**

**you look at the third paragraph under "Dietary Fluoride Supplements," it reads: "The evidence for using fluoride supplements to mitigate dental caries is mixed. Use of fluoride supplements by pregnant women does not benefit their offspring." Did I read that correctly?**

A. Yes.

**Q. So in this review, 2001, CDC determined that use of fluoride supplements by pregnant women does not benefit their offspring, correct?**

A. They're citing a study which makes that finding, yes.

**Q. Okay. And if you turn to Page 21 --**

A. Okay.

**Q. -- you see again there's a section titled "Dietary Fluoride Supplements"?**

A. Uh-huh.

**Q. And it reads: "The only randomized controlled trial to assess fluoride supplements taken by pregnant women provides Grade I evidence of no benefit for their children." Did I read that correctly?**

A. Yes.

**Q. And Grade I evidence is the best quality evidence that CDC has in its characterization of the**

**literature, correct?**

A. I have to say that I am unfamiliar and not remembering the grade levels of what the evidence was.

**Q. Okay. Well, why don't we turn to Page 19. That might refresh your recollection.**

A. Okay.

**Q. Do you see the box at the bottom titled "Grading system used for determining the quality of evidence for a fluoride modality"?**

A. Gotcha. Yes.

**Q. And Grade I -- and take your time to review it. But after you've reviewed it, would you agree with me that Grade I is the highest quality of evidence in this grading system?**

A. Okay. I've reviewed it; and yes, I'll agree --

**Q. Okay.**

A. -- to your statement.

**Q. So the CDC's conclusion in 2001 is that they had good evidence, high-quality evidence, to show that fluoride supplements do not provide a benefit for children when given during pregnancy, correct?**

A. Yeah, when given to pregnant women.

**Q. Okay. And is CDC aware of any information published subsequent to 2001 that would in any way**

**change that conclusion?**

A. I can answer in my personal capacity. I am not aware of any additional studies.

**Q. Okay. And you are the director of the CDC's Oral Health Division, correct?**

A. True.

**Q. Okay. And the Topic 9, as we talked about, Topic 12 does ask for CDC's position on the --**

A. Right.

**Q. -- benefit or lack thereof of prenatal fluoride exposure, correct?**

A. Provides a benefit to teeth, right.

**Q. Right. So I'm entitled to CDC's knowledge on this question.**

A. Uh-huh.

**Q. And is CDC aware of any study published subsequent to 2001 that in any way changes this conclusion that CDC reached at that time?**

MR. DO: Objection to the extent it mischaracterizes CDC's position as stated in Exhibit 53.

THE WITNESS: Apologies. I'm having a hard time following the logic of your question.

**Q. (By Mr. Connett) Well, we're looking at a 2001 document written by the CDC, right?**

A. Published in a CDC publication.

**Q. Right.**

A. If you look, this was written by people of part of a work group.

**Q. Okay.**

A. And so it had a work group providing it had had outside reviewers and it had CDC authors. So it was a collaborative work like the 2015 PHS guidelines.

**Q. Okay. Are you saying that this document does not reflect the conclusions and assessment of the CDC?**

A. In 2001 it does.

**Q. Okay. So my question now is: Is there any information that has been that CDC has obtained subsequent to this 2001 report that in any way changes CDC's assessment on the lack of benefits from prenatal fluoride exposure?**

MR. DO: Objection to the extent you're misstating CDC's position.

THE WITNESS: Yeah. I am not aware of any additional information/evidence outside this that informs our position that we submitted in response to this topic.

**Q. (By Mr. Connett) Okay. So would it be**

**fair to say this -- is there any other document that I can look to that the CDC has published that comments on the benefits or lack thereof of prenatal fluoride exposure?**

A. I'm not aware of any.

**Q. Okay. And so the CDC is not aware of any research to -- I'm sorry. Strike that.**

**The CDC is not aware of any research demonstrating a benefit from exposure to fluoride, fluoridated water, during pregnancy, correct?**

A. That's a slightly different version, exposure to fluoride versus fluoridated water. And I would say answers previously and similar, I'm not aware of the impact or anything that changes the fact that we don't have good evidence that suggests there's benefit.

**Q. And in Topic 12, the notice says "CDC's position, if any, on whether prenatal exposure to fluoridated water provides a benefit to teeth and the basis for said position." So am I correct in understanding that CDC makes no claims and does not contend that fluoridated water during pregnancy provides a benefit to the teeth of the offspring?**

MR. DO: Objection with regard to form.

THE WITNESS: As we stated back in the

document, we don't have a position on this matter.

**Q. (By Mr. Connett) Okay. So if a pregnant mother wrote an e-mail to CDC asking if I drink fluoridated water during my pregnancy, will that provide a benefit to the teeth of my baby, CDC would not answer yes to that question, correct?**

MR. DO: I'm not sure how to object to that question to be honest. Objection, form.

MR. CONNETT: Just say form.

MR. DO: Form. Form, foundation, calls for speculation.

THE WITNESS: If we were to get an e-mail as such, we would summarize our understanding of the evidence in saying we have not found evidence that supports -- that shows benefit to the child if ingested -- if community water fluoridation or some other form of fluoride is ingested by the mother.

**Q. (By Mr. Connett) Okay.**

A. And we would also instruct them to consult with their attending physician to make those choices.

**Q. Okay. So I'd like to now move on to Topic 13 which has set forth in our notice as "CDC's position, if any, on whether exposure to fluoridated**
