# Supplementary material for: The sugar industry’s efforts to manipulate research on fluoride effectiveness and toxicity: a ninety-year history
Source: Environ Health. 2025 Sep 29;24:62. doi: 10.1186/s12940-025-01154-x (PMC12477810; doi:10.1186/s12940-025-01154-x)
Supplement: Supplementary file 3 — Supplementary Material 3. [file 12940_2025_1154_MOESM3_ESM.pdf]

Additional File 3

For:
The sugar industry's efforts to manipulate research on fluoride effectiveness and toxicity: A ninety-year history
Christopher Neurath

Transcript of meeting of the Newburg-Kingston Fluoridation Trial advisory committee.

Proceedings: Meeting of the Technical Advisory Committee on the Fluorination of Water Supplies; February 28, 1946; New York City.

Yellow highlighting of portions discussing neurological effects of fluoride, endocrine effects, thyroid effects, and plans for studying adults.

P R O C E E D I N G S

\- - -

MEETING OF THE

TECHNICAL ADVISORY COMMITTEE ON THE FLUORINA-

TION OF WATER SUPPLIES

\- - - - - -

February 28, 1946
New York City.

C O N T E N T S

	Page
Review of the Newburgh-Kingston Study to date	2
Review of the composition and objectives of the over-all medical survey	8
Discussion of the possible relationship of fluorine to the endocrine glands	41
Discussion by Dr. John A. Caffey on x-ray findings in Newburgh examinations	49
Discussion by Dr. Isaac Schour of findings in survey made in Italy in areas of endemic fluorosis	54
General Discussion	57
Vote of appreciation	70
Adjournment	71

The meeting of the Technical Advisory Committee on the Fluorination of Water Supplies, held at the offices of the New York State Department of Health at 15 Maiden Lane, New York City, on February 28, 1946, convened at 2:10 P.M., Dr. Harold Hodge, Chairman, presiding.

Present:

Dr. Harold Hodge
Dr. John A. Caffey
Dr. David B. Ast
Dr. Charles R. L. Cox
Dr. B. F. Mattison
Dr. Isaac Schour
Dr. H. I. Ingraham
Dr. F. B. Amos
Dr. E. S. Rogers
Dr. A. Schleisinger
Dr. William J. Gies
Dr. D. E. Overton
Dr. F. W. Gilcreas
Dr. S. Z. Levine
Dr. K. Baine
Dr. George J. James
Dr. H. L. Chant
Dr. S. B. Finn
Dr. M. Pomeranz
Mrs. Polansky

CHAIRMAN HODGE: I would like to call the meeting to order.

This is the third meeting of the Technical Advisory Committee on the Fluorination of Water Supplies. I believe I see reflected in all our faces the same interest that we had on our first two meetings.

DR. GIES: More.

CHAIRMAN HODGE: Thank you, Dr. Gies!

Since there are a few here who have not attended previous meetings, I will ask Dr. Ast to introduce the newcomers.

DR. AST: Sitting next to Dr. Gies, on my left, is Dr. Schour, who is Professor of Histology at the University of

Illinois. Dr. James is seated next to Dr. Schour. Dr. James is Assistant District Health Officer in the Middletown area, and responsible for the Newburgh part of our study. Dr. Ingraham, next to Dr. James, is the Director of the Division of Communicable Diseases.

As we go around, our next new man is Dr. Overton sitting next to Mr. Grimm here. Dr. Overton, will you just put your hand up so they know you are here? Dr. Overton is the Clinic Physician, and is directly responsible now for all pediatric examinations in the study in both Kingston and Newburgh. Mrs. Polansky, sitting at my right, is responsible for the statistical part of the study.

CHAIRMAN HODGE: I imagine all of you received a reprint summary. Dr. Ast submitted it to the New York Journal of Dentistry, and it was published in the January 1946 issue as a progress report on fluoride investigations. Personally I found this to be interesting reading and a good idea, and I would like to commend Dr. Ast, and suggest that this become an annual custom for a while.

That leads us to a very natural place to call on Dr. Ast for a review of the study to date.

Dr. Ast!

DR. AST: The first meeting of this Advisory Committee, together with the Departmental Working Committee, took place on April 24, 1944. This was following the assurances of cooperation

in the proposed Newburgh-Kingston Study by the city authorities in both Newburgh and Kingston. At that time, the dental caries fluorine hypothesis was presented as was a detailed plan for the Newburgh-Kingston Study.

The mechanics of the dental phase of the study presented no serious problems, but the question of possible side effects of fluorine required much discussion. At that time Dr. Dean, of the Public Health Service, presented some data which led him to believe that we were not justified in subjecting the people of a community to such a study, until more conclusive evidence of the safety of fluorine therapy could be demonstrated. He presented data on small population groups which indicated a possibly higher prevalence of cataract, changes in the nails and bone changes at 7-8 ppm F. After careful consideration of this testimony, the Advisory Committee approved the initiation of the study, but suggested that a very comprehensive pediatric as well as geriatric examination should be included to pick up, at the earliest possible moment, any signs of cumulative effects of the 1 ppm F which Newburgh's water was to contain.

While the original plan called for a pediatric examination, Dean's testimony suggested far more detailed examinations which might necessitate bone marrow smears, Addis counts, slit lamp examinations, in addition to the routine blood and urine analyses, x-rays of the hands, forearms and tibias for the children, and the skull, pelvis and tibia for the adults. This

necessitated further planning and delayed the initiation of the basic examinations, although the dental examinations were started in June 1944.

In August 1944 we met again, and themechanics of the medical plan as well as the dental plan were approved. We were also encouraged, at this time, by learning that data coming from the Public Health Service studies seemed to indicate that up to about 5 ppm F in drinking water, there was no storage of fluorine in high school boys and young adults. We were also advised that Dean had changed his mind concerning the justification for such a study as proposed, and had initiated a similar caries fluorine study in Michigan, without benefit of medical examinations.

At this meeting we also discussed the advisability of seeking financial support from some private foundation, but that was subsequently obviated by getting the State Budget Director's approval, which assured us of a budget of approximately $25,000 a year.

To date, all of the basic dental, pediatric physical examinations, x-rays and some laboratory examinations have been completed in both Newburgh and Kingston.

In Newburgh, 3,589 children,ages 1-14, had dental examinations, and 235 school children had their salivas examined for L. acidophilus counts.

In Kingston, 3,104 children, ages 1-14, have had dental examinations. L. acidophilus counts are now being made.

All of the Newburgh dental records have been tabulated and analyzed, but Kingston's have not yet been completed. An interesting observation was made in comparing the DMF rate of the 12-14 year old children in Newburgh with the same age group in studies reported by Dean in fluorine free areas, and a similar age group in Brantford, Ontario, which is also engaged in a caries fluorine study. In each instance the DMF rate was 7.4 per child.

Physical examinations, as well as x-rays were made in Newburgh for 536 children under 1-8 years of age. Pooled specimens of urine were examined for fluorine. In Kingston, 507 children under 1-8 years of age had physical examinations, x-rays, blood and urine analyses.

I shall not attempt to present any of the details of the medical examinations, but shall leave that to Dr. Overton, who has recently completed the examinations in ~~Newburgh~~ Kingston, and to Dr. Caffey, who will discuss the x-ray findings.

Fluorine was added to Newburgh's water supply on May 2, 1945. During the winter months we have noted a little difficulty in maintaining a uniform concentration of 1 ppm F. However, this difficulty has apparently been overcome by changing the point of application of sodium fluoride, so that a better mix is obtained. I have just come from the Newburgh filtration plant, where I saw the data for the past week, and the concentration is gratifyingly uniform now. No mechanical difficulties in

feeding the fluoride have been encountered. A full year's supply is on hand at the Newburgh plant, so that we need not be concerned with running short of the salt.

While the field staff is not yet complete, it now consists of an associate research dentist, a clinic physician, a public health nurse and a stenographer. To this permanent staff we have been able to add a supplemental nurse, a temporary clerk and the part time services of a laboratory and x-ray technician. In addition, a dental hygienist and consultant public health nurse and statistician from the central office have participated, as well as volunteers from both cities.

The physical plant in each city is quite adequate to our needs, and in Newburgh, a well equipped laboratory has been set up.

We are now at the point where we are ready to begin re-examinations, both dental and medical. While the plan calls for medical re-examinations every three months for the under two year olds, and semi-annually for the over two year olds, this was not possible up to now, but with our medical personnel on a full time basis, it should be possible to meet this schedule in the future.

CHAIRMAN HODGE: Thank you, Dr. Ast!

I think we will have opportunity for discussion and questions after each of the presentations on the agenda. I would like to start the ball rolling by asking Dr. Ast about

one item in the report he has just made. As I remember it, Dr. Dean cautioned us when he told us about the incidence of cataract, nail and bone changes on a high intake of fluorine in the drinking water, that the information was in a sense classified and not his. He asked us I believe not to pass that word along.

If this review is to be circulated at all, has that been cleared with Dr. Dean?

DR. AST: This is not to be circulated except within our own group. These are minutes of our meeting and our minutes are part of the documentary file in the central office. The information is not circulated.

CHAIRMAN HODGE: In that case I would like to ask the cooperation of each of you because the data on which Dr. Dean made his report comes from unpublished material in the Public Health Service files and was not Dr. Dean's to divulge for public consumption, and consequently it is not ours. It is interesting and important and we considered it seriously and still do.

Are there other questions or discussions?

Just for the sake of my information, how many of you have been down to Newburgh and Kingston?

... About six so indicated ...

CHAIRMAN HODGE: I took the opportunity a couple of months ago to visit and certainly had a grand experience, not the least of it was to see Dr. Overton in his clinic. We got there just at the last of a busy day and I guess the last

squirmer was just having his or her snow suit put on as we got there. Dr. Overton told us we had missed the excitement. Everything was quiet and peaceful. I don't know whether he can get that kind of atmosphere into his description.

He is going to review the composition and objectives of the overall medical survey. Are you going to separate that from your relation of fluorine to the endocrine glands?

DR. OVERTON: One thing will more or less merge into the other, unless you want to stop for a break. I would be glad to have anyone stop me at any point in that because it will bring up questions I would like answered right in the middle of this rather than wait till we get to the end.

CHAIRMAN HODGE: Fine!

DR. OVERTON: Recorded knowledge of medical evidence of the action of fluorine on organs of the body other than the teeth is very limited and this virgin field of research should be a challange to pediatric workers. Fluorine enters the body via food,in vegetables which grew in certain mineralized soils; 2, water, drinking water in certain areas of Texas; 3, dust, as in cryolite mines; 4, accidental poisoning, commercial roach exterminator; and 5, overdoses of commercial mouth washes, lozenges or dentifrices containing fluorine if and when they are on the market.

Fluorine has a physiological action on calcium metabolism bordering on the pathological. Naturally the effect is

noted on the most calcified organs, the bones, in which hyper-calcification occurs, producing osteosclerosis of degree varying with the length and intensity of exposure to fluorine. The possible effects of fluorine on the thyroid and parathyroid glands have been mentioned in medical literature.

The skin and other ectodermal tissues are repeatedly reported in medical literature to be affected by fluorine, but there has been no definite proof that the effects mentioned are actually evidence of fluorosis.

I. Infants and Children

These examinations will be performed to determine the effect, if any, of fluorine on growth and development, and to determine conclusively the safety of ingesting 1 ppm fluorine in water daily.

In Newburgh, the study area, the initial examinations will be made on a group of 500 children approximately equally distributed as to sex.

The age distribution of the initial group of children examined will be as follows:

Under 1 year of age	100
One year of age	100
Two through four years	200
Five through eight years	100

Those are the same figures which have been presented to you before, the age distribution.

CHAIRMAN HODGE: That was the schedule which you hoped to hit?

DR. OVERTON: Yes.

CHAIRMAN HODGE: And the examinations have now been performed?

DR. OVERTON: In both cities.

CHAIRMAN HODGE: How close did you come to your schedules?

DR. OVERTON: Very close.

DR. AST: If you want the figures we will be glad to furnish them.

DR. OVERTON: I would rather have specific questions.

A representative sampling as to race and economic status of the child population will be obtained. Children are enrolled from families which intend to reside within the city limits for the duration of the study. Each year an additional group of 100 infants under the age of one year will be enrolled in the study. Each child will be re-examined periodically until he reaches his fourteenth birthday. Children under two years of age will be examined every three months; thereafter, examinations will be performed semi-annually unless there are special indications for more frequent examinations.

In Kingston, the control area, comparable groups of children will be examined once a year.

A complete medical history is taken at the time of

each child's initial visit. An interval history is taken on subsequent visits. This history is recorded by a specially instructed public health nurse under the supervision of the examining pediatrician.

The effect of fluorine on the people of Newburgh will be studied in detail in search of minute detectable deviations from normal, whether toxic or beneficial, which may be attributed to the fluorine which has been introduced into the drinking water. The deviations from normal are expected to be very slight, if any appear at all, particularly during the first two years of the study. It is difficult to find in medical literature records of infants and children which can be used as normals for this minute comparison to the growth and development of children of Newburgh in the future. Available growth and development records do not indicate if the children received fluorine in their food and water. All such tables become antequated as soon as they are gathered and do not indicate what will be normal in 1947 or 1950 or 1955 when the height of children may be taller and other effects may be noticed under the influence of better prenatal care, greater attention to calcium foods and vitamins and diminution in the ravages of pneumonia and streptococcal disease.

Physical examinations -- the physical examinations will be performed by a certified pediatrician assisted by a registered nurse. Extraordinary emphasis will be placed upon the

examination of specified tissues and organs which are suspected of being affected by fluorine. Specific points to be observed include the following:

A. Epidermis:

1. Rash or discoloration.

2. Change in sebaceous glands.

3. Change in sweat glands.

4. Change in elasticity of connective tissue.

B. Nails:

1. Grooves, irregularities of shape.

2. Irregularities of color; mottling; white marks.

3. Irregularities of texture; brittleness.

4. Irregularities of thickness; cornification.

C. Teeth: (See dental examinations)

D. Eyes:

1. Discoloration or inflammation of conjunctivae and sclerae.

2. Gross change in visual acuity.

3. Nystagmus or strabismus.

CHAIRMAN HODGE: Dr. Overton, in connection with the eyes, are you attempting to measure the extent of the visual field?

DR. OVERTON: We have no arrangement for that.

Ears:

1. Change in auditory acuity via air conduction.

2. Change in auditory acuity via bone conduction.

F. Brain:

1. Evidence of increased intracranial pressure.

A form for recording the results of the physical examination is attached. As indicated, various physical measurements (standing and sitting height, circumference of head, circumference of chest) are made as part of the physical examination. I have the form and anybody who wants to can examine it. There is nothing unusual about it.

Laboratory examinations:

The following are performed at each visit on the blood:

a. Hemoglobin (recorded in grams) Sahli

b. Total erythrocyte count.

c. Total leukocyte count.

We would like to have a photo-electric hemoglobinometer. On the erythrocyte count, we take a total erythrocyte count in all cases. At least we did in Kingston. That is a little more extensive than the plan called for.

Urine: here we make a determination of color, reaction to litmus, specific gravity, presence of sugar and albumin, and an examination of the centrifuged sediment.

DR. MATTISON: For the sake of completeness, was not something done about making a smear on each of those cases, doing a differential?

DR. OVERTON: It comes in the plan later. I will read that.

DR. MATTISON: I see.

DR. OVERTON: Bones:

a. Antero-posterior x-ray films of right hand and forearm.

b. Postero-anterior x-ray films of both tibiae.

Examinations performed on specific indications:

1. Blood:

a. Differential leucocyte count if total leucocyte count is less than 5,000 or more than 11,000.

At this point I would like to make just a comment about the preliminary survey of our records of the white blood count, and the make-up of those differentials.

We encountered a surprising wave, every few weeks, of children with leucocytosis who came in apparently well. The younger the baby the more likely it was to have a leucocytosis. The babies under one year of age seemed to run very definite leucocytosis in waves. It seemed to be seasonal by the week. They occasionally gave a history of recovering from upper respiratory infection but not always.

Scattered through the whole three months' period we encountered a high percentage of eosinophilia, which I think would bear further study when we have our records analyzed.

I considered some of those children may have in their histories something with reference to an allergy. Some of them were just getting over virus infections I think. The others had probably a parasite of some kind. We found evidence of pin

worms in a couple of them.

I am not prepared to talk about the figures now. It is a preliminary thing. It will bear investigation.

DR. CAFFEY: Whose standards are you using for normal white counts? What standards are you using for those?

DR. OVERTON: We consider a count that is outside of the range 5,000 to 11,000 total is abnormal and make a note of it and write a letter to the doctor.

DR. CAFFEY: I think you should use a wider range for infants. I think Dr. Levine ought to comment on that.

DR. OVERTON: The doctors of Kingston are more or less used to that range. It is easy enough to write a letter about that. We write the letter but don't tell them what to do about it.

DR. CAFFEY: I don't think we should call them abnormal unless over 15,000.

DR. OVERTON: The only thing we do about it -- we don't necessarily call them abnormal -- is we count the differential smear and I save all the differential smears if you ever want to study them in the future.

DR. CAFFEY: There is a new book which has the Washman's figures on the normal. I think Dr. Levine should talk about this rather than myself. I would not consider 15,000 counts as abnormal according to Washman's figures. That is not an easy subject. The range of normal variation in those

counts is considerably higher in children.

DR. LEVINE: I think for the purposes of the study it makes very little difference what limits you are taking and it would merely increase the data available for analysis later on if you take a range between 5 and 11 instead of 5 and 15.

I assume Dr. Overton at present is not differentiating between normal and abnormal counts, but merely taking figures, and he will have differential counts on all babies with 11,000 white cells and over. They may show nothing at all. I think Dr. Overton is interested in getting a comprehensive study. If he has those additional data they may well make the study more complete. Isn't that your point?

DR. OVERTON: Exactly! May I add that we went better than the criteria called for. We looked at the smear and if we saw some eosinophils we counted the differential count anyway. If we saw anything that looked queer about the smear we counted it.

DR. LEVINE: I think the body of data which Dr. Overton is collecting, irrespective of its direct application to this study, is going to be tremendously interesting because if you get 500 counts and 500 urines, and 500 periodic examinations of growth and development, and follow some of them fourteen years it will prove a very worth while endeavor.

DR. CAFFEY: The waves of fluctuation are those to be correlated with the different technicians or is your technique

identical?

DR. OVERTON: There is one for the whole series.

DR. CAFFEY: That would enhance the value.

DR. POMERANZ: May I ask are these tests done at the same time of the day, with the same relation to food, etc.?

DR. OVERTON: They were taken at mixed times, from eight in the morning to four in the afternoon. I can find out what time of the day they were taken if we save certain records. I know what time of the day we did them. We made no effort to save the records but they have not been thrown away.

DR. POMERANZ: I wonder if there is some significance to that.

DR. OVERTON: We made no effort to save the appointment sheets but they indicate what time the appointment was.

DR. LEVINE: I think it would be worth while to keep that in the permanent data.

CHAIRMAN HODGE: One other question, have you run across any serious blood dyscrasias?

DR. OVERTON: No, it is an unusual thing. In 500 counts like that there has been very little anemia, no severe anemia. We have not found a severe white blood cell dyscrasia. The eosionophil counts are the only thing that is of note.

The City of Kingston I think is very pediatrically minded. I have never been in a city where children of all types seem to have a pediatrician as they do in Kingston. I think Dr.

Mattison can bear me out on that.

DR. MATTISON: I think that is true. As you pointed out when you first came there, the pediatrician patients are not just of one economic group. I cannot explain it but it is so.

DR. OVERTON: That is the only way these two cities are different as far as I can see. Newburgh has a definite class of people who go to the pediatrician. Kingston on the other hand, its pediatricians have a cross-section of the city in their practices.

DR. ROGERS: It should be just pointed out when the records are complete there should be a thousand children.

DR. BAINE: Five-hundred in each.

DR. ROGERS: There will be a loss. We are working out a system of replenishing the losses to make up for them.

DR. LEVINE: It is my understanding you are examining a hundred new infants each year.

DR. OVERTON: I will continue:

b. Wasserman test on suspicion of syphilis.

2. Bone Marrow:

a. Smear if anemia is present.

3. Urine:

a. Addis count of urinary sediment if albumin or casts are present on routine examination of urine.

I have not ordered bone marrow or Addis count in

Kingston.

In addition to the above, quantitative determinations of fluorine in pooled specimens of urine will be performed at intervals as facilities permit.

c. Special examinations:

1. Test of visual acuity by Snellen charts of children 3-14 years of age every six months.

2. Slit lamp examination of children 3-14 years of ? age every six months.

3. Audiometric tests of children 5-14 years of age every six months.

4. Psychometric tests of children -14 years of age every three years.

I have not set the lower figure there.

II Adults

These examinations will be performed to determine the effect, if any, of fluorine upon the mature organism and particularly to discover whether there is any cumulative action of fluorine in this age group in the dosage employed.

A total of 150 adults more than fifty years of age, equally distributed as to sex, will be enrolled from the study area. As many as possible will be residents of an institution for the aged located in the study area. The originally enrolled adults will be followed for the ten year study period unless they leave the institution or study area before the end of the period.

Additional adults meeting the requirements as to age and residence will be enrolled to replace those lost from the study.

CHAIRMAN HODGE: Have you an audiometer at the schools or clinic?

DR. OVERTON: That has not been contemplated as we are probably going to be able to use the audiometric records of the Kingston schools because they start with the third grade children. Children of the third grade are eight years of age. You cannot take audiometric records of children under the third grade in large groups because they watch each other and cheat.

To go on with the adult examinations, these will be performed annually unless findings indicate more frequent checks. In the medical history, the emphasis will be upon more recent events. In the physical examinations, the same parts of the body will be emphasized as in the examinations of infants and children. The same routine blood and urine examinations as well as the examinations performed on specific indications will also be done. In the adult group, the following x-ray examinations will be made: antero-posterior views of skull and pelvis, lateral view of right or left tibia (same at each examination). Tests for visual acuity, slit lamp and audiometric examinations will be done at intervals of six months.

That completes the overall picture of the composition and objectives of the medical survey. This has been in the future tense because it is written as a plan. Examinations, as

you pointed out, were made in Newburgh a year ago and were made in Kingston in November, December, January and February.

... The examination referred to is as follows:

Medical Examinations - Babies and Children

Type of Exam.	Age Group	Indications	Frequency	By Whom	Remarks
History - complete	all	routine	1st visit	RN and/or M.D.	
interval	"	"	each revisit	"	
Measurements	birth -14 yrs.	"	" visit	RN or M.D.	crown to heel<br>circumference-skull<br>" chest<br>sitting height
Physical Exam.	birth-2 yrs.	"	ev. 3 mos.	M.D.	staff and private on fee basis
(see Med.record form)	2-14 years	"	ev. 6 mos.	M.D.	" "
Laboratory<br>Blood-hemoglobin)	all	"	every visit	Staff Technician	
W.B.C.)	all	"	" "	"	
Rbc.)	all		" "	"	
Differential	---------->	if W.B.C.is less than 5000 or more than 11,000	" "	"	
Bone Marrow	---------->	if evidence of anemia by above tests	" "	taken by M.D.	
Wassermann Test	---------->	history or clinical or X-ray evidence	" "	" "	To be done at State Lab.

Medical Examinations - Babies and Children

Type of Exam.	Age Group	Indications	Frequency	By Whom	Remarks
Urine<br>Urine reaction<br>color<br>Sp. gr.<br>sugar<br>albumen<br>cent. sediment	all	routine	every visit	technician	
Addis Count	-------->	if albumen and/or casts found in routine exam.	as indicated	"	
Quantitative fluorine	all (pooled specimens)	routine	"	"	To be done at State Lab.
X-ray<br>A.P. hands<br>forearms<br>tibias	(birth- 2 yrs.<br>(<br>(2 - 14 yrs.	routine<br><br>"	ev. 3 mos.<br><br>ev. 6 mos.	"<br><br>"	Interpretation by roentgenologist on fee basis
P.A. tibia					
Visual acuity	3 yrs.-14 yrs.	"	ev. 6 mos.	------->	Prevention of Blindness Bureau
Slit lamp	3 - 14 yrs.	"(?)	ev. 6 mos.	------->	Prevention of Blindness Bureau
Audiometer	5- 14 yrs.	"	ev. 6 mos.	?	Assistance from Dept.of Education
Psychometric	? - 14 yrs.	"	ev. 3 yrs.	?	Assistance from Dept. of Mental Hygiene (?)

DR. CAFFEY: I think you ought to be congratulated on your start. This is a question of how much you can do. It is important.

Somebody asked the question before of overweighting this with technical procedures, it might interfere with the experiments. I think this is really a splendid start to have gotten as far as we have, considering all the difficulties, as Dr. Levine said, the finding of normal children. It may be a means for establishing the norms in this part of the world. It is not very well established.

DR. ROGERS: I would like to mention in connection with this medical examination, as probably most of the committee know, the Children's Bureau is helping by underwriting part of the expense of the allocation to the State Department of Health for these studies, and it was customary to have them inspect the plans they support and make suggestions, valuable suggestions with regard to them. The suggestion was made when we took this up with them about, I guess it was about a year ago this spring, wasn't it,Dr. Baine, that there was a special committee or subcommittee of the National Research Council, of which I believe Dr. Baine is a member. Anyway there is such a committee that has addressed considerable attention to long range observation studies of growth and development, and other long range projects. It was believed that the experience of the group which had had that sort of experience would be valuable to us and they

made that suggestion, which we readily accepted. We have not as yet submitted our plan to their review and for suggestions to them. I wanted the committee to know about it because they might hear indirectly that we were submitting the plan to other groups and might wonder just where that leaves the committee.

It leaves it simply this way that we would take for granted your approval for our attempting to get advice of that kind, but that the National Research Council Committee in this instance is purely giving us the benefit of their opinions and judgment and is in no way acting as an advisory body superseding the functions of this body at all. We hope to have the plan drawn up, that Dr. Overton has sketched for you, in more detail, and to send it to that subcommittee. Possibly Dr. Overton and others of the staff may appear there and discuss it with them, and then will be prepared, I hope, at our next meeting with you to have any suggestions they may have to make ready for your consideration.

I did want you to know about it. I think we should have done it before now.

As you probably all know, there has been a great deal of work getting this study started during the war time. I feel since I am thoroughly in the background of this thing, Dr. Ast in particular is to be congratulated to have kept the thing going with the uphill strunggle most of the way. He has had marvelous cooperation from the localities. I think our district officers

are to be complimented on the work they have done in keeping that local interest going. It is hard to keep local interest going in these long pauses which have occurred from time to time. It has been done.

We feel we are rolling fast enough with the combined dental-medical examination so that the maintenance of interest level is assured, but it is interesting that the public is really intensely interested in this particular project, and it is important to keep them so, to make the study much more valuable, that their co-operation be assured.

Dr. Baine, I don't know whether you care to comment any further, on that National Research Council.

DR. BAINE: Not particularly except our feeling was that the National Research Council has access to a great many people who have worked on many studies over many years, and it might be desirable for them to get a group to simply review the plans, make a review of the plans and see if they have any suggestions to offer.

It seemed to us that a study as fundamental as this one needed all the advice they could get from any place, and so our suggestion was that they might get additional help from that group. I don't know that we have gotten anywhere.

DR. ROGERS: We have cleared with them. They expressed their willingness to do it. They seem quite pleased to have the opportunity. I should have brought the letter with me. I

have not reviewed it recently. I cannot remember the correct title of that committee.

DR. BAINE: I think they expected us to write the National Research Council, and I would take it to the Committee on Child Health.

DR. ROGERS: Is that the name?

DR. BAINE: The Committee on Child Health.

DR. SCHOUR: Mr. Chairman, in the effort to make this medical examination complete, as complete as possible, especially in relation to growth and development, has the problem been considered in terms of taking photographs of each of the children with the graph background, so that as you go over a ten year period of examination you have a visual record of the child?

It may not involve much in the way of expense or time but it might be a definite advantage for permanent records.

DR. OVERTON: We have not included that in the plan. It sounds like an interesting thing to do.

CHAIRMAN HODGE: That is a good idea.

DR. BAINE: I think one of the reasons perhaps it was not considered was because you did not expect to get any sort of a gross bodily change. All you expected to get, if you got anything, would be very minute changes which would not appear in gross growth and development.

DR. SCHOUR: I think we all have a feeling we are going to get some worth while information, even with the hope

that we will find nothing wrong but you will have a normal picture all the way through. Then it would be something to be proud of.

DR. OVERTON: That is one very definite way to measure growth and development with photographs against a background that indicates size.

DR. SCHOUR: In addition to your weight and height measurements.

DR. AST: You recall that in this progress report there was a report by McClure on height and weight experiences for selectees who came from areas of endemic fluorosis and from non-fluorine areas, and while those were of course for adults, they showed no significant differences at all.

I don't know whether your suggestion relative to infant and child development may be more significant. I don't know.

DR. SCHOUR: I think it would help to prove that there are no significant changes, if that is the case. Objective records are going to help in this and you can never describe as well as a photograph will do.

DR. POMERANZ: Have you ever thought of using the Wetzel chart as a method for screening? Is that worth considering?

DR. LEVINE: I think that would be done towards the end of the study. You would take individual children and use

serial measurements to see whether they remained in their norm so-called or whether they deviate on one side or the other.

DR. POMERANZ: You would not apply it until we accumulated a period of years of observations?

DR. LEVINE: You cannot use it.

DR. POMERANZ: You cannot use it except in single patients.

DR. LEVINE: That or the Sontag, or some type of grid would be very valuable in summarizing the development of the two groups.

DR. SCHLEISINGER: That is something we could consider in detail later on as to which method would be the one of choice before we do it.

DR. SCHOUR: The National Research Council Committee could advise you on that.

CHAIRMAN HODGE: Has the examination of the older group been made?

DR. OVERTON: We are not prepared to start that yet.

CHAIRMAN HODGE: Just a point of information, at the present rate of conducting examinations of the children, how many months is it going to take per year to keep abreast of the current load in the two cities?

DR. OVERTON: I frankly doubt if I can keep up the schedule. It may be possible to work a little faster returning to Newburgh because there may not be as high a rate of cancella-

tion of the appointments. In Kingston they canceled one out of four appointments without warning.

DR. BAINE: Was that because of the time of the year? You did those in November and December.

DR. OVERTON: We had to compete with Santa Claus, Christmas and the bad weather and influenza in epidemic form. One afternoon we could not find out why nobody showed up until we discovered that every school in the city had an entertainment that afternoon, and the mothers told us they were all sick and at home and we found everyone went to school to the plays.

DR. AST: To get back to Dr. Hodge's question, we thus far estimated about three months to do the pediatric examinations. That is for the whole group. If we have to break that group up into pre-schoolers, that is the under two years of age and over two years of age group, it will probably take about ten months out of the year to get all those examinations in if we follow the schedule.

CHAIRMAN HODGE: I would like to bring up perhaps the schedule some place later if you will tell me to later. The question about the expert examination of the data as already on hand, in that regard I can easily conceive that just the collection of these notes and records on so many children over a long period is going to provide a gold mine for somebody, but I see no particular reason why the fellow who does all the digging should not get some of the gold. I wonder whether some arrange-

ment should not be made so that Dr. Overton has time to collect and digest and correlate this information. After all, he has got the most interest in it. If we call upon him for practically continuous service in the clinic, isn't that a mistake as far as being in the most favorable position to profit year by year from the examinations?

DR. MATTISON: I would like to add a point there. I think that I have the most difficult problem in one way in keeping up interest in the city that is not getting anything out of this, and I think if we could have something like that with even a few small points coming out each year that it would make a lot of difference in keeping the medical profession and the dental profession interested.

CHAIRMAN HODGE: Perhaps an annual report by Dr. Overton to the Medical Society and an annual report by Dr. Ast and Dr. Finn to the Dental Society?

DR. MATTISON: That would be splendid.

CHAIRMAN HODGE: I have some experience with cold data. In general I have the feeling that it takes a couple times as much time to work it up if it is just two or three notebooks back than if it is just recorded. At the rate information is piling up here it is going to be a serious bulk to handle.

I certainly don't have any suggestions as to how to get around this difficulty but I would like to raise it.

DR. LEVINE: There is one possibility. When we origi-

nally devised the program for medical examinations we knew very little about the potential hazards in the infantile age group particularly. We were led to believe from information that we received that there were potential hazards. Now we feel much safer about the procedures and it seems to me that the data that we are accumulating on the original program is not so much to protect these infants and children anymore than to derive normal data.

Why is it necessary to stick rigidly to our original routine of routine examinations at intervals of six months over two year intervals, of three months under two years? Could not Dr. Overton be relieved of some of that by spreading the interval and only examining those infants and children more frequently who show defects and deviations but for the normal procedures possibly to spread the interval? I don't know whether that is feasible but at least that might relieve Dr. Overton of some routine work.

DR. ROGERS: I wish the committee would consider that a little more. I am very glad you brought up the point you did. I might add the only way I was able with Dr. Ast's help to get a man of Dr. Overton's caliber to do that -- after all he is a Board qualified pediatrician with a lot of experience in the clinical department -- was to promise him that there were such opportunities. Otherwise we would not have gotten him at all.

However, we must not lose sight of the fact that the

study is primarily a dental study. We don't want to forget in working this out. I will tell you what our administrative conclusion is that it is primarily a dental study and that is the main line of our approach, so that if we can cut down the amount of work and still protect the fundamental purpose of that dental study and by cutting it down increase the opportunities for collateral studies, that is grand, because while I say it is primarily a dental study, I am perfectly willing to say that secondarily and not very much different from primary is the fact that there are these opportunities we hope for a lot of collateral studies which will be entirely medical in their field of interest. So that you advice on the extent to which we could reschedule these examinations along the lines of your suggestion would be very helpful to us.

DR. CAFFEY: I would like to suggest it was brought up at one of the last meetings that the studies of the children in Kingston,who are by definition normal children, the medical studies could be reduced to a minimum but the dental control is there. I think this was discussed before the advisability of taking x-rays for Kingston. I personally would be satisfied with films of the children getting fluorine water. There does not seem to me much point in taking films of the children in Kingston who are not getting fluorine.

What we are looking for are signs of fluorosis in the bones. We are not looking for normal ones in Kingston, so that

will reduce Dr. Overton's duties considerably if that were done.

DR. AST: If we are looking for a picture of growth development, if we are expecting to pick up any changes in growth and development, won't it be necessary to have the data from Newburgh for comparison with the Kingston data to determine such changes?

DR. CAFFEY: You can make a choice of using Kingston as a medical control or using the controls we have for normal children from many standpoints. I think the blood studies by Washman have been done more carefully probably than we could do them. These were done in Denver.

I mention the overweighting of this thing and making it topheavy, of gathering data from the normal group in Kingston which to me seems is not necessary. If you want to follow a normal group in Kingston and have facilities, fine, but it does not appeal to me as being essential for this study.

DR. BAINE: I should hate to see you lose some of the things that you want to get on your Kingston group for control. I think it might be possible, however, to cut down the amount that you do because, after all, you are trying to get from them certain norms for growth and development to compare these others against, whereas with the Newburgh ones we are trying to get the things, trying to pick up early signs of toxicity plus finding out if there was any deviation in growth and development, so that maybe they have to be done more frequently than your Kingston.

DR. CAFFEY: That was my suggestion. Cut down! I

would rather see the cut made in Kingston than in Newburgh.

DR. ROGERS: I would still like to keep the thousand or five hundred for any purpose.

DR. CAFFEY: If you have the facilities that is fine. If you are limited in facilities, I think the cut should be made on the Kingston side.

DR. OVERTON: Could I give you a few figures of how long it takes to examine the children, of Kingston for instance? We examined 507 children in Kingston in two and one-half months because we took out two weeks at the Christmas holidays when it was not practical to conduct the clinic.

We could return to Kingston once a year, if we allowed two and one-half to three months to do it. I think we might get through very readily in less than three months the second time because the people would be familiar with it. I think we can get re-examinations easier than the first examination.

No matter how the child rebelled, I can say that universally people expressed their appreciation for the clinic as they left when they apologized for the battle the children put up in some instances, and were ashamed of the children and wanted to come back. I had nobody show any indication that they intended to skip the clinic next year.

There are not enough months in the year for me to examine 500 children of Newburgh over again because the whole year has gone by. They are all due for a re-examination, plus

100 babies, and I am still to see the babies under two years of age every three months and the older children every six months, and get up to Kingston. There are just not enough months to get that accomplished unless I am surrounded by a larger staff of technicians.

I have been doing the x-rays in Kingston, and I took the blood. I capped the pipets with rubber bands and sent them over to the laboratory, so you see I added extra work to the time. Therefore, we never were able to do more than approximately 15 cases a day. If I wished to do 20 cases a day I did not have an arrangement with the laboratory to take 20 blood counts. I had to stop at approximately 15. I could not work any faster although I tried to myself.

I am glad you brought that problem up because of two things. I don't see how we can physically meet the schedule. I really do feel quarantined in that clinic and have to forego a lot of things that I would like to go to which would assist me in the clinic work because I have children scheduled three weeks in advance and we don't dare cancel appointments because they are very difficult to fit in again.

I wish that the program could be scheduled along a practical line that we could adhere to rather than have this fine program and only get back there once a year.

DR. ROGERS: How much time do you think you will save if we get the technician who is supposed to be there with x-ray

work and taking the bloods? How much will you cut off your time?

DR. OVERTON: If I am going to make physical examinations of these children, I can do 20 a day.

DR. ROGERS: As opposed to what, 15 did you say you were doing?

DR. OVERTON: We scheduled, after we got used to it, about 22 and actually accomplished about 15 a day. We had to schedule 22 to get 15, and we were always afraid that they would all come and we would not be able to do the laboratory work. It never happened.

DR. ROGERS: I just want to make it clear, it is not our intention for Dr. Overton to keep on doing that. It just happens, as we know, it is easier to get a janitor than school teacher but the reverse is true of doctors and technicians. We hope to get a technician before very long but who will of course have to have some training in the work under Dr. Overton's supervision and assistance.

DR. POMERANZ: In connection with the statement, if it is the object of the statistical community to have two communities close together to see the skeletal development, that would be one objective, in one with regard to the possible effect of fluorosis and the other as normals. ?

If you are taking the Kingston group to compare them as to the possible effects of fluorine in the Newburgh group, then I would say, as Dr. Caffey, that that study is unnecessary, that

is, the Kingston, because in the radiological and radiographic literature there are innumerable reliable tables giving the normal epiphysial development. Since you are not specifically interested in the epiphysial development per se, you are interested in retardation presumably as the result of possible toxic agent in the water, then I say you could eliminate completely the Kingston group because we know the normals, and then continue the study with the Newburgh group. That will save money, time, plates, and everything else.

CHAIRMAN HODGE: At least you certainly agree that there would be the point of maximum reduction.

DR. ROGERS: As far as x-rays are concerned.

DR. LEVINE: The same applies to other laboratory procedures.

DR. CAFFEY: All of them I would say.

DR. LEVINE: If you get a normal blood count on a baby, in Kingston, certainly any abnormality coming thereafter will be the result of some spontaneous change and would have no bearing on being used as a control for the Newburgh study.

DR. CAFFEY: No relation to fluorosis there, the fluorination of water in Newburgh.

DR. LEVINE: Even if you should see ten babies with leukemia on the fifth examination it would not mean anything.

DR. SCHOUR: The follow-up is not significant if you want to have a baseline. That saves a lot of time.

DR. ROGERS: There is a point which Dr. Ast made which I would like to raise for your consideration. In connection with that I am not in a position to question at all the statement that the base lines are well understood in the norms. I don't doubt it at all. I wonder whether or not those base lines which are going to be used are constantly reflecting the possible changes which Dr. Ast brought out, the changes in habits of living, in diet, which is the type of thing occurring constantly.

If we measure our own growth by the difference between World War One and World War Two in selectees in physical stature, if we measured growth today on the basis of the chart which has been established, no matter how thoroughly back in the time of the First World War, we would conclude that it was very abnormal growth and therefore, is there some advantage in some of these examinations, at least in carrying along current base lines?

DR. LEVINE: Yes, I would completely agree with that, but it would seem to me an x-ray for that is not necessary. It would seem that other physical examinations might be indicated at intervals, possibly of a year, two years or three years rather than short intervals. I think that is indicated.

DR. CAFFEY: The ranges of normal variation which occur during this study in growth and development will be within the normal range, that as an individual you could not detect it. The spread is not going to be very much different, the things you use for differentiating normal and abnormal.

DR. LEVINE: For the purpose of publication we need a current base line.

DR. ROGERS: It could go back longer. If we could keep enough ~~of~~ going to keep the Kingston group ~~alive~~ together we would not have to do all examinations. If on indication you want to ~~any~~ have another series of x-rays complete in Kingston five years from now, we still would have those children coming in. We could get that revised base line for you at any time. Perhaps we are safe on that.

DR. CAFFEY: If you have facilities I would be in favor of all of these. The question of limited facilities has come up here. If you are going to use the facilities to the best advantage they should be used on the Newburgh side of it. I am for all of it if you have facilities. If you have not facilities, I point out what I think could be reduced without hurting the detection of toxicosis in children in Newburgh and that is making your reduction in Kingston except the teeth.

DR. INGRAHAM: Why not lengthen the examinations to six months in Newburgh for those under two?

DR. ROGERS: I don't know.

CHAIRMAN HODGE: I would like to resolve this discussion if I can in this way: Dr. Overton, Dr. Ast, Dr. Finn, and perhaps Dr. Rogers, maybe one or two others would be good enough to get together to go over the schedule as it stands to suggest a new twelve months' schedule about what can be done in twelve months beginning the first of June or July, whenever

is a convenient starting point, and include a suggestion or any suggestions about needs for technical assistance, or where they would be strategically useful and send that around to us for our comments.

If there is considerable disagreement on the part of us individually as we answer the schedule sent to us, perhaps we could get together again and devote a meeting to this because this really is a very fundamental point, and the choice or the lack of selection of a control group is right at the center of the interpretation ultimately of the data I am sure.

Would that suggestion -- it is rather a complicated one -- be all right?

DR. SCHOUR: I would like to add one other suggestion that when this committee budgets the time involved, it see whether it would be possible to arrange a schedule whereby Dr. Overton would have let us say every sixth day free to study and take inventory of the findings as they come along. This may be an ideal setup but I think it would be a very efficient one.

CHAIRMAN HODGE: I believe, Dr. Overton, you were going to say something about the endocrine glands.

DR. OVERTON: The thyroid and parathyroid glands are concerned with the calcium metabolism. Fluorine exerts influence upon the process of calcification of organs and tissues of the body. Fluorine poisoning disturbs organs which are biologically of ectodermal origin and regulated by the parathyroid glands, that

is to say, the teeth, skin, nails and hair. A search of medical literature reveals very little information about the relationship of fluorine and the thyroid and parathyroid glands which will bear critical analysis. This virgin field is a challenge to the pediatric worker.

Here are a few random references from the medical literature which are virtually all of the information which we have been able to unearth. It bears out my contention that it is not supported by critical analytical proof. Dr. Philips of the University of Wisconsin calls attention to the fact that he is finding greatly increased fluorine content in diseased thyroid glands in humans. Dagmar C. Wilson studied dental fluorosis in the Punjab Plains of India and obtained a history of the existence of endemic goiter associated with cretinism in areas where fluorine was recognized geologically. She stated that in England the distribution of Fluorine in the soil corresponds closely with the distribution of endemic goiter, and where dental fluorosis was shown by mottled enamel, it was ascertained from the inhabitants that goiter still occurs. She states her suspicion but does not prove that fluorine in drinking water is one factor in the causation of endemic goiter.

Leo Spira, in London, points out that chronic fluorine poisoning causes disturbances of organs biologically originating from the ectoderm and regulated by the parathyroid glands, that is to say, the teeth, the skin, nails and hair. He makes this

not too clear statement: Signs and symptoms characteristic of fluorosis may be produced by interfering with the normal function of the parathyroid glands.

I am going to read that statement again:

> "Signs and symptoms characteristic of fluorosis may be produced by interfering with the normal function of the parathyroid glands".

DR. SCHOUR: Who says that?

DR. OVERTON: Spira.

DR. SCHOUR: Without any data on it? He has no data on it? Are those just theoretical considerations? Has Spira got any facts?

DR. OVERTON: As far as I know these are random statements. I cannot find any critical analytical material to back them up.

Further investigation will be needed before deciding whether the disturbance in the concentration of calcium in blood and tissues is brought about by fluorine in a direct manner, or secondarily through an alteration in the parathyroid glands.

Pavlovic and Tihomirov found the parathyroid glands in the experimental fluorosis to be pale. Hanch, et al, working along the same investigation, noted the abnormality but concluded that it was not great enough to suggest interference with functional activity of the glands as a whole. They made the observation that the parathyroid glands in rats do not undergo any significant change, either grossly or microscopically, with the

administration of sodium fluoride. Sutro expressed the belief that the changes in the teeth and bones of rats to which sodium fluoride was administered are due principally to a chemical disturbance which is unrelated to the parathyroid glands since subcutaneous injections of a parathyroid extract given up to a total of 700 units over a period of two to three months neither retarded nor prevented the appearance of mottled teeth.

There is very little medical literature available on the relationship between fluorine and the thyroid and parathyroid glands which will bear critical analysis and it is hoped that this Technical Advisory Committee will have some definite suggestions how to pursue the subject further.

In going through the physical examination, I pointed out that I have stressed the examination of the organs that are ectodermal in origin and the organs which are under the control of the parathyroid and thyroid glands. I welcome suggestions from the committee whether to pursue this further and if so, how?

DR. POMERANZ: If you pursue that further you will need greatly augmented tests. You will have to do calcium and phosphorus in the blood and in the urine and feces.

DR. CAFFEY: There are some measurements which can be made perhaps on the skeleton. Perhaps the single important finding in hypothyroidism is delayed maturation. I think that will become evident in serial films. That is what you are doing and that perhaps is one of the most important single findings.

DR. LEVINE: A psychometric examination?

DR. CAFFEY: Yes.

CHAIRMAN HODGE: Delayed eruption time of the teeth.

DR. POMERANZ: As far as the texture of the bone is concerned, what we do know of parathyroid and thyroid and fluorosis are completely opposed and don't bear out these articles. They don't substantiate what these men say.

DR. SCHOUR: In reference to the thyroids, I am going to perhaps refer to that later in connection with my report, but in two communities in which we did examinations in Italy the thyroid enlargement was very prominent and extensive. Perhaps 90 per cent of the population showed it, so certainly it would be very much worth while to look as hard as one can for any evidence of thyroid involvement and not be satisfied that you may not see any obvious enlargement.

As far as the obvious considerations are concerned, they are very complex and confusing, and I think if you want to get some information on that we would really have to work to an experimental analysis rather than hope to get the answer through a clinical study.

That brings up the point which Dr. Pomeranz mentioned if we are going into that it means we are expanding and it involves additional technical assistance and time.

DR. ROGERS: I think your consideration of this particular point at this stage ought not to be bound by those fac-

tors. If it is really important in your judgment then I think it is up to us to try to consider the ways in which it might be done. If, on the other hand, it is not of great importance, that is important for us to know too.

I know in Boston -- I hoped Roy Johnson might be here -- some of the men working in his laboratory expressed considerable concern over the possible effect of fluorine in relation to the endocrine system. Their feeling was that it might interfere with the ~~functions~~ metabolism of iodine. I don't know whether it has greater affinity or displaces the metabolism of iodine but it might. They felt that something should be done about it. We thought one way of doing it would be to perhaps take a very small sample and call upon ~~your~~ the good graces of some of your men in the city -- I won't look directly to Dr. Levine -- but somebody in that locality who could help us on a small sample of an intensive study of this type of thing.

DR. SCHLEISINGER: If you are talking about experimental work, it is possible to obtain radioactive fluorine and see --

DR. ROGERS: I would like to introduce Dr. Schlesinger. He came in late, so he was not introduced. He is now on our staff from assignment. He is also qualified as a pediatrician, acting as an assistant director of our M.C.H.Division.

CHAIRMAN HODGE: That is a good suggestion. It appeals to me very much. We played with radioactive fluorine a little

bit. Certainly for short time studies it is entirely feasible. By short time I mean six or eight hours.

The methods of separation of thyroxin, for instance, are well established and perfectly known. There is a good little piece of work for somebody there.

We never have any trouble thinking up things to do. This is the greatest group for thinking up things to do that I ever saw.

DR. POMERANZ: May I interrupt for a second, not that I want to delay things? As long as you are thinking of new things, you are impaled upon the horns of a dilemma, because if you do calcium and phosphorus, then why not phosphatase? I am not so sure that I would accept many readings on phosphatase level. There are, curiously enough, a certain number of people who will do a phosphatase which will be accepted by many other people.

DR. LEVINE: Is it necessary actually to develop a program in this direction at the present time? We have these children. If clinically they show no gross impairment, if their epiphyseal development showed no delay on x-ray, if their psychometric examinations are within the normal range, is that necessary? If any defects develop as time goes on to justify any extensive study, then I think that would be the time to consider it rather than to undertake a program now which on the basis of the evidence available will probably yield negative results.

DR. CAFFEY: And will have to be tested on these

children anyway no matter what you found.

DR. LEVINE: It seemed to me that procrastination is in order at this point.

CHAIRMAN HODGE: Did you have something to add to this, Dr. Overton?

DR. OVERTON: No, I have nothing to add.

DR. ROGERS: I wonder if we may assume that it is the consensus of the Committee, Dr. Hodge, and I think this point is very important to us because it may come up on the part of the critics of our work, which we will undoubtedly have and desire to have. We desire to know that.

DR. LEVINE: If you want to do a few simple things on the basis of iodine particularly, the fluorine replacing the iodine, do a cholesterol in the blood. That would be one additional feature. That would protect you. That is the simplest. Then again you may do a creatine urine because with hypothyroidism there is an excess output of creatine in the urine. You have the urines. That would give you an additional bit of information without doing anything further.

CHAIRMAN HODGE: Did I understand you to suggest some blood iodines?

DR. LEVINE: That is a difficult procedure.

CHAIRMAN HODGE: Very few people in the country can do it. If you could enlist some help on that that would be well.

DR. LEVINE: In Chicago, Curtiss does it and in Texas

Fushina does it. There are a few but the creatine in the urine, and the cholesterol in the blood I think would definitely prove or disprove the existence, with x-ray, of significant hypothyroidism.

CHAIRMAN HODGE: So, perhaps, in the schedule we were speaking of a few moments ago it might be a small special group of .020, really a small group could be picked out with indications for a few blood or urine examinations such as Dr. Levine suggested.

Suppose we leave that in the hands of Dr. Overton and Dr. Ast and Dr. Rogers for the moment.

I think we are ready, Dr. Caffey, to hear your discussion on the x-rays.

DR. CAFFEY: Dr. Ast mentioned this to me recently and I have not a detailed report. I don't think it is necessary. The films that have come to me have come all from the Newburgh group before fluorine was put into the water, so that you would expect there are no signs of fluorine intoxication in the bones. The only findings have been a number of normal variances in bones, found in any group of normal children, but they are important, particularly such things as transverse lines. That would confuse the interpretation if they developed, if you found them in this group and did not have a base line to start from.

The films have been excellent from the x-rays which Dr. Finn took in Newburgh, and the records of the films are all

marked, and the technical part of the work has been done very well under great difficulties from the technical standpoint and the x-ray facilities. Dr. Finn did these all himself.

We did find some children that had poliomyelitis, had atrophic bones and muscles, and some children had synostosed joints, and I suppose those reports go back to the pediatrician. I don't know what the clinical diagnosis was.

All the children in this group are not normal from every standpoint;I am sure there are a couple of small legs from probably polio atrophy but the preliminary findings of the bones are very essential if you are going to detect early sclerotic changes.

Again it would not make very much difference to me how many children in Kingston had transverse lines because the longitudinal study of these children who were getting fluorine water is the thing I am interested in rather than in seeing how many transverse lines there are in normal children in Kingston.

Dr. Overton's films from Kingston I have seen a small sample of, and they are as good as are done in most hospitals. As a matter of fact, most of them are excellent. A few of them are over-exposed. The children moved but I did not see any films that were not satisfactory for interpretation. That is about all the data we have. We found a wide spread of normal variation which you would expect in such a group as we have done in Newburgh.

I think the record is the important thing if they are going to be used. Every patient has a number and an envelope which is clearly labeled and the interpretation is on a chart. It has all worked out very well.

DR. AST: There is a list of the findings.

DR. CAFFEY: Dr. Ast has made a satisfactory start on it from the record standpoint.

CHAIRMAN HODGE: I was impressed in the few films which I saw by these normal variations. Not being a radiologist accustomed to seeing normal variations, I was really surprised. I had no idea that bones in a normal child could look so peculiar.

DR. CAFFEY: That is one of the most characteristic things of children, to vary in color, size, bones and everything else but it is very important to have this base line if we are going to make any interpretation.

DR. AST: If any of you are interested, I brought down about 20 of those films with the x-ray interpretation if you would like to see them later. We don't have a viewing box but if we get through before sun down you can use daylight. You might be interested in seeing them.

DR. POMERANZ: I have not seen any of the films. I would like to ask Dr. Caffey if he has seen any effects of excess vitamin therapy, which might have some effect upon subsequent events.

DR. CAFFEY: I think not. The metaphyseal changes we have seen in children who do not get an excess of vitamins.

DR. OVERTON: When you get the films from Kingston, which will be very soon, there will be three in particular I want you to look at because we had three children show carotenemia Their skins were peculiarly yellow, yet you could not say it was an icteric color because the sclera were clear. Little by little it dawned on me it was the result of eating excessive pureed canned vegetables. All three had a lot of squash in their diet and plenty of carrots and beets and they had a weird yellow color. Dr. Mattison saw the worst one of the three. Two of them had stopped eating pureed foods for two months and were still yellow. I would like to see if their bones show any changes. That would be an indication of plenty of vitamins. Presumably there were plenty of vitamins in the diet. They were very healthy children.

DR. CAFFEY: I would be very interested in seeing those. I would be very much surprised if excess carotene would be indicated on the skeleton.

DR. OVERTON: They will be indicated.

DR. SCHLEISINGER: Would you be more interested in the complete history, to show the abnormalities such as transverse lines?

DR. CAFFEY: Many of these are patients of private doctors and I think the thing we are going to do is send a report to them that these have been found and let them use them the way

they want them. I am not able to do much on the clinical side really, but the people who see them clinically I think should know about this.

DR. ROGERS: I cannot let this moment go by without expressing our thanks to Dr. Caffey for the tremendous amount of work this involved. His willingness to do this with his busy schedule certainly has been very helpful.

DR. CAFFEY: That may be a factor on my views on Kingston.

CHAIRMAN HODGES: I saw the drawers of x-ray films and then the sheets with the name and the record which Dr. Caffey examines and the little comments written over on the side, the signature and date down at the bottom. I don't know how he did it. Do you do anything else?

DR. CAFFEY: It is interesting to see normal children because very few normal children are examined roentgenographically. It is an expensive examination. Everyone we have in the hospital has something wrong with them, so the films are not taken otherwise. I am interested in this but I would like to keep it within a reasonable volume.

CHAIRMAN HODGE: We are not charging Dr. Caffey anything for his education.

DR. CAFFEY: I hope the records keep up as well as they are. What is more, they should be accessible so that people can use them. I think this is really a good start, your records,

not mine particularly.

CHAIRMAN HODGE: I think that is a very important point. The record system up there seems to be enterable from several different ways so that it looks like a first class job.

Are there any other comments or questions about the x-ray? I would certainly urge you to take advantage of Dr. Ast's offer. I have not seen the pictures. I have no idea what he brought down but I suspect he has brought down some of the interesting samples of the normal examinations.

DR. AST: The ones Dr. Caffey made notations on.

CHAIRMAN HODGE: Those are very worthy of being seen. I looked at some, where he has made notations on them.

If there is no further comment, Dr. Schour, recently returned from Italy. I always like to hear Dr. Schour talk, and this time he is talking on survey areas of endemic fluorosis in Italy. We are especially interested in that.

DR. SCHOUR: The background of this is the fact that Dr. ~~Mattison~~ Massler and I were dental members of a nutrition mission that went to Italy to study the effects of malnutrition there, and soon after I arrived I found that there was a small community about 35 miles north of Rome that had endemic fluorosis for apparently generations, and we started to make an examination and found a significant reduction in caries, which was of no surprise to us because I don't think there is any question any longer about that, but we did find a high incidence of peridontal disease.

On the basis of clinical examination and on the basis of x-ray examination, the intra-oral x-rays showed changes in the bones and in addition you could examine a full mouth x-ray and on the basis of previous experience you would say, well, that is an individual who should be about thirty-five years of age and you looked up the record and it came from a patient who was only twenty years of age. The x-ray age was older than the chronological age.

DR. CAFFEY: This is the mandible you are talking of?

DR. SCHOUR: Yes. As I mentioned before, there was a very high incidence of thyroid enlargement, not of the goiter type, but a sort of fibrotic type. There were no other indications of any other symptoms like cretinism.

After making this study, the question naturally came up: is this thyroid involvement and are these bone changes related to fluorine or do they happen to be just coincidentally associated with some other factor?

Then we were successful in finding another community not far from Naples which was somewhat different inasmuch as the population showed a great deal of malnutrition in comparison to the group in Compagnano di Roma which had a better nutritional status, and there the changes were very much the same.

We also had an experience similar to the one that Dr. Mattison referred to. After we had a good start in Compagnano di Roma some people protested that they were not getting anything

out of it. We had a chance to solve it in a much simpler way. We decided to give a bar of soap to every patient who would come and we had a lot of customers.

On the basis of this we had no evidence to say definitely that these changes in the people, the peridontal changes, were more prominent than the peridontal changes which we found in other areas in Italy. In general there is less decay and more peridontal disease in Italy than in the United States, but this group stood out because of a much higher incidence of peridontal disease.

The gingival cones and bone changes may be related to fluorine or not, but I think there is enough indication there to justify our being on the lookout for it.

The brings me to the specific problem in relation to dental examinations. I think that the emphasis has been made about this being primarily a dental study. I would like to perhaps disagree with it. First of all, I think we should agree that this should not be a dental study but an oral study, and that means we should go beyond the enamel and dentine. We should go to the gingiva, have a record of it and have a record of the x-rays.

Personally I am pleasantly surprised that you are going ahead with a program, a very thorough program of taking x-rays of the long bones, but I am disappointed that in an x-ray program of this sort the obvious and simple procedure of taking

an x-ray of the alveolar bone has been omitted up to this point.

DR. AST: May I ask you to be a little more explicit in what you mean by peridontal disease? Just what changes did you notice in the gingiva and what changes were noted in the alveolar bone?

DR. SCHOUR: In the gingiva you have a higher incidence of inflammatory changes which may be acute or chronic and which may be of local or systemic conditions. We don't know, but the fact that there is such a high incidence in this community, higher than elsewhere, would make you think that there is some systemic factor to this. It is easily evident by just a direct examination. They are acute and chronic inflammatory changes which are not difficult to recognize.

In the x-rays there were indications of osteoporotic changes which were quite distinct.

DR. CAFFEY: At what ages?

DR. SCHOUR: They ranged, we had intra-oral x-rays beginning with ten and fifteen year olds up to adult. In the adult you have an accumulation but I think it was more significant to find some of these changes in the teen age group, in the adolescent group.

DR. CAFFEY: It is not possible in younger children.

DR. SCHOUR: Yes, we have taken x-rays, intra-oral x-rays at four years quite definitely.

DR. CAFFEY: I was going to ask what age.

DR. SCHOUR: You can do four year olds and five year olds. We have taken a number of x-rays. This is material we have not had a chance to work up yet, but it seems to me an intra-oral x-ray of your 500 children group and a kodachrome picture of the gingiva would be a very worth while addition to your regular routine. You cannot go wrong and you are safe.

DR. POMERANZ: Did you find those osteoporotic changes were more likely to occur with those patients who had large thyroids?

DR. SCHOUR: We did not have a chance to correlate it, but the thyroid enlargement was present throughout the population beginning with the relatively young children through the adult period.

DR. ROGERS: How much fluorine?

DR. SCHOUR: I don't know. We were not satisfied with everything. The water has been sampled in Italy and the samples have been sent to the Public Health Service in Bethesda.

But on the basis of the mottled picture I would judge the amount of fluorine would run between 3 and 4 ppm.

DR. AST: I would like to make this point: I am wholly in agreement with Dr. Schour, with what he just said that we ought to get as much information as we can from our oral examination, but going through the literature, in reading about osteosclerosis in areas of cryolite mining, where individuals are getting as much as 3 per cent of sodium-aluminum fluorides, in no

instance has any bone destruction been evident. Of course there were no examinations recorded by those who were studying the problem, no examinations made of the mandible. All of the examinations were made of the long bones and of the vertebral column, but in every instance what they have found was a thickening of the periosteum and the endosteum, a narrowing of the marrow canal, osteophytic outgrowths, ankylosis of the vertebral column, but bone destruction was absent from the syndrome in all reports, so it makes me wonder a little why we should be getting any bone destruction in the mandible as the result of fluorine when it has not been evident in any other bone.

DR. SCHOUR: Of course, there is a difference between the alveolar bone and all the other bones in the body, that is, that the alveolar bone grows much more rapidly than others and grows continually throughout life as long as you have got teeth to support it. In that sense we have an indicator there that may show the changes more readily than any other part of the body.

You recall the work on hypothyroidism by Jaffe, he pointed out that the bone which grows most rapidly is the one which is most sensitive. The site of most rapid growth is the site of more sensitive reaction, and I think in the alveolar bone we have got it made to order, but we certainly at least should look at it.

DR. CAFFEY: I think, Dr. Ast, your evidence is that the mandible is not examined in cases where sclerosis is found in

other bones. It is probably a very important consideration.

DR. SCHOUR: There is another point. I think it would be very valuable under any circumstances to make a correlation, whatever it may be, of the changes between the long bones and in the mandible in the same patient. We cannot go wrong on getting information.

DR. AST: Just as a point of information, it presents no serious problem to us at all. In fact, we already have portable x-ray equipment which could readily be used for this purpose, and I am certain that we can get a substantial number of these x-rays so that probably by our next meeting we will be able to report that something constructive has been done in that respect.

DR. SCHOUR: I would like to add one other point to emphasizing that, we should attempt an oral examination rather than just a dental examination.

Dr. Overton indicated that would be worth while, that is, examine the skin. If you want to examine the skin you should also want to examine the oral mucosa and the tongue is very handy to look at. If that could be incorporated just as a matter of being extra careful, I think that it would be worth while.

DR. OVERTON: It is part of my physical examination to examine the tongue. Would you tell me what to look for in particular?

DR. SCHOUR: The first thing I would do is to set up a kodachrome apparatus, which is very simple. I would take koda-

chromes of the tongue just as I take kodachromes of the gingiva. Technically it takes 1/50 of a second to take the picture. Actually it takes three seconds perhaps and you have a permanent record, and the ten cents which it will cost to pay for a kodachrome is a good investment.

DR. OVERTON: Will you tell me what to look for and record in the physical examination if I don't get the kodachrome?

DR. SCHOUR: I would look for a change in the papilla, a differential examination of the filiform and fungiform papilla and the color of the tongue. I am going at this on a theoretical rather than on the basis that there is going to be a change. I personally have a hunch there won't be, but we have got to look for it. We have got to be ten times safe.

DR. AST: As another point of information for you, I have been pursuing this problem on the side by trying to get information from other individuals, and I have just had a letter from Dr. Fred McKay in Colorado Springs, who has perhaps done more of the pioneering work in endemic fluorosis than anybody else, and who also happens to be well qualified as a periodontist, in fact, that is his specialty. While he has no records on which to base his findings his letter indicated that he has seen no greater prevalence of peridontal disease among the natives of Colorado Springs, where they have 2.6 ppm F in their potable water than among others who have not been exposed to fluorine. That is the result of just his clinical experience.

DR. GIES: There appears to be a number of observations in this field to lead some of us I think to agree with Dr. Schour that we ought to be alert, not necessarily expecting but to be alert.

For instance, two weeks ago, Dr. Phelps Murphey of Dallas, Texas, addressed the New York Academy of Dentistry and told quite casually about the Army records indicating certain sections where the fluorine quantities are minute in the drinking water but enough apparently to have a profound effect in the prevention of dental decay, but instituting uncommon amounts of periodontal difficulty. He was very vague in just what was meant by the latter. There was some allusion to cementum disintegration and things of that kind but it left open of course the possibility that whatever was noted might have been due to something else. It does seem with this general mixup of records and intimations and suggestions that we ought to be alert to that possibility.

Perhaps the study of the adults will be especially significant in that relation if that could be increased in intensity. Let us be alert but not necessarily expecting anything.

CHAIRMAN HODGE: A few years ago, I don't remember whether it was in connection with one of the early meetings of this committee or not, I got to wondering about this peridontal problem and wrote Trembley Dean a letter if he ever looked at the gums of the people he looked at in all these areas. He said that he had not paid any particular attention to them, but that he had

a kind of a hunch that there was more peridontal difficulty in children where the drinking water contained very little or no fluorine. This may be adding straw to a straw man but I agree this is a good place for somebody to do some investigating.

DR. SCHOUR: Would it not be important at the end of this study to make a definite statement, if possible, that there is no peridontal difficulty?

DR. GIES: Alert to the possibilities is what you mean.

CHAIRMAN HODGE: On a point like that there is an absolute necessity for having a good big control group. That would be necessary in peridontal disease. How many dentists specializing in children's dentistry could give you offhand the incidence of peridontal disease?

DR. CAFFEY: The examination of the control group is continuing. This is part of the dental program.

DR. AST: There are some three thousand children in each city.

DR. CAFFEY: That should be kept up in Kingston as part of the dental work.

DR. AST: I don't know whether we can x-ray 6,000 children in addition to the dental examination, but I think we can get a significant sample for that purpose.

DR. SCHOUR: We have two difficulties in getting a thorough checkup on the peridontal conditions. First of all, as

Dr. Hodge pointed out, there has not been enough work done in the field. We don't have the periodontal index, some quantitative discipline, where we can get the DMF's comparatively simply.

Another point must be kept in mind, especially when you study the adult population, if you are dealing with people who have less caries you have people who have teeth for a longer period of time with gingiva which were exposed to longer periods of time of disturbances, so you have to correct, or let us say, in the adult group, if you have people who are new to caries, there is going to be a larger part of your population that reaches old age with teeth and they will have an increased condition in the way of peridontal disease.

On the other hand, the people who have lost teeth are not going to have peridontal disease because there is no gingiva to work with.

DR. AST: In other words, we have a geriatric dental problem.

DR. SCHOUR: One of the promising indications of a good piece of research is that it brings up a lot of additional problems.

CHAIRMAN HODGE: I might comment on this osteoporotic, osteosclerotic point. Fluorine does odd things. I think perhaps I have mentioned before to this group that in rats fed on a rachitogenic diet the formation or the development of typical rickets is retarded by including fluorine in the diet.

DR. SCHOUR: How much fluorine, a lot of it? If there are excessive doses you have retardation of growth; a slow growth will automatically cut down the possibilities of rickets.

CHAIRMAN HODGE: I think there is not sufficient data to get that point. On the other hand, if you take, and we took rachitic rats and gave them a good diet, vitamin D and fluorine, the fluorine has the effect of preventing normal healing.

DR. AST: Say that again?

CHAIRMAN HODGE: Fluorine in the diet of a rat which is tending to develop rickets will prevent the development of rickets. Fluorine in the diet of a rat given a healing diet, this is a rachitic rat, prevents normal healing. There is an odd kind of calcification which occurs.

DR. OVERTON: May I quote a little item I ran across, a simple statement of Dr. Lemon, a pediatrician, in Amarillo, Texas? He called attention to the fact that it is harder to prevent bowing of the legs in rickets in children who have lived in endemic fluorosis areas although they received adequate amounts of vitamins in the diets. That is another one of those items without anything to bear it out.

CHAIRMAN HODGE: That checks the osteoporotic. It sounds as if it interfered with the normal laying down of bone. The reason I quoted this rickets thing was that Dr. Schour indicated that people raised on the high fluorine diet had perhaps insufficient calcification whereas these other individuals pre-

sumably on normal bone development having superimposed a poisonous amount of fluorine developed a hypercalcification of the skeleton. Those things may not be contradictions at all. They may be two aspects of the toxic effects of fluorine.

DR. OVERTON: Dr. Levine, if a child is a potential case of rickets and remains one size and does not grow for a little while, is it true you don't see the evidence of rickets until it starts to grow, and the fluorine intensifying the speed of calcification would bring out the evidence of rickets, make it externally visible?

DR. LEVINE: Rickets is always evidenced most markedly at the sites of most rapidly growing bones.

DR. CAFFEY: It is in proportion to the velocity of growth. Of course the mandible has no end on end growing, no epiphyses and metaphyses. There is a different type of growth than in the mandible. That is why it is so important to take films of the mandible, because it is a different problem.

DR. LEVINE: Am I to understand, Dr. Hodge, in the dental examinations the gums are not examined?

CHAIRMAN HODGE: Dr. Schour.

DR. SCHOUR: Not with the same attention, the same attention as the caries.

DR. LEVINE: That is surprising to me.

DR. SCHOUR: Of course the pediatricians I am sure do both.

DR. LEVINE: They do both superficially I am sure.

CHAIRMAN HODGE: Dr. Finn, would you care to tell us what you do when you give a child an oral examination?

DR. FINN: We spend about three to four minutes on every child, covering with explorer each surface of the tooth, which does not leave much time to inspect the gingiva, but I think it should be done. It is worth taking time out for a dental examination, to make a special effort to examine the gingiva. That should be done.

DR. OVERTON: Dr. Ast, our examinations in Kingston included the examination by the dental hygienist after I got through with examining the child. Have you examined her records? Do you think she makes a thorough examination of the gum?

DR. AST: She does the same thing that Dr. Finn just indicated. It is a dental examination, pure and simple, with a mouth mirror and sharp explorer to detect any defects in the enamel of the tooth, to note fillings, to note missing teeth.

Here again we were started in perhaps a very narrow line of thought, and how to prevent dental decay was our prime objective, and we did not let ourselves get involved in any collateral studies. But again I repeat, that this is and should be in line with our thinking and that we ought to make every effort to get that information.

DR. BAINE: May I ask if you are running into the problem of the ~~amplification~~ application of fluorine by the dentists in the

community at all and the thing is going to be ruined?

DR. FINN: I don't think many of them are using topical applications. I think the dental profession around Kingston and Newburgh are a little skeptical.

DR. BAINE: It might happen when they know this other community is getting fluorine that there might be some pressure from the patients to do something for their teeth.

DR. FINN: I think the dental profession is very cooperative in the smaller community.

DR. ROGERS: It would be a matter of record any way, won't it? You have records that would relate the two.

DR. FINN: There are not any of them using it on children. They may be using it on the adults but we are not examining the adults.

DR. ROGERS: I think it is a point to investigate however.

DR. AST: We asked the local dentists if they used topical applications to please let us know on which children they were used and when, so we could add that information to our records and the dentists in both cities agreed to do that.

We have not investigated recently but that is another thing that we ought to get at right away and contact the local dentists and find out what they have been doing about it.

I would like to make another point. You probably have been reading in the lay journals recently about the incorporation

of fluorides in mouth washes and in dentifrices, but in the current issue of "Dental Research" which just came in, Dr. Bibby of Tufts College Dental School reports on a two year study in which they studied children and young adults who were using a fluoride containing dentifrice with no apparent protection, which is contrary to our thinking in relation to topical application.

The topical application of a fluoride solution is giving very promising results.

Knudson reports on a two year study in which he is getting as much as 40 per cent reduction in the teeth with a two per cent treatment of sodium fluoride. Incidentally this application was made only once two years ago and was not followed up with subsequent applications and still at the end of two years he had a 40 per cent protection, whereas in this two year study reported by Bibby, and he used from a 0.1 to 0.01 per cent sodium fluoride incorporated in the dentrifice. No protection was afforded to children and dental students who were included in the study.

CHAIRMAN HODGE: There are several present who have not taken part in the discussion. I hope you have not been bashful and thought we did not want to hear your comments. Do any of you have something you would like to add?

DR. FINN: I might talk about the lactobacillus studies. The L. acidophilus in the oral cavity is supposed to be

an indicator of the amount of dental decay. It is even more than an indicator. It is a prognosticator of things to come, the amount of caries to come within say the next six months, and we have analyzed the first 100 salivas in Kingston and we have gotten negative results which would indicate that there would not be any cavities or a low number. We got a percent of 16.3.

In analyzing the salivas in areas which were fluorine free there are found anywhere from 14.9 to 19.2 per cent, so our percentage fits right in the middle. In counts of 30,000 or over, which indicate a high degree of caries, their figures are 48.8 to 54.6. Ours was 52. We are right in the middle there.

DR. ROGERS: You expect a reduction due to the fluorine?

DR. INGRAHAM: There is no variation in the two cities.

DR. FINN: I have not analyzed the figures in Newburgh. I think they may be a trifle higher. We do have a little more decayed teeth in Newburgh. I am not sure. We have not analyzed them.

CHAIRMAN HODGE: If there are no further comments, I will bring the meeting to a close.

DR. BAINE: I would like to say one thing, if I may speak for myself, I think the staff should be congratulated in the way the study has gone along.

CHAIRMAN HODGE: I think that is important enough to ask that it be put in the form of a motion.

DR. BAINE: I will make the motion.

DR. LEVINE: I will second that.

CHAIRMAN HODGE: All those in favor please say "aye"; contrary "no". The "ayes" have it and the motion is carried.

I think that gives the unanimous pat on the back or orchid which you desire.

I should also say, I am sure I speak for the entire membership, this seems to get more interesting as we get along, and I am looking forward eagerly to the results as they come in. I trust that we can meet again in not so quite a long interval. We will await your call of course, but when some of these statistics are available I think we would like to get together and chew them over.

I certainly would like to express my appreciation to each person who has taken part in the meeting this afternoon.

We will stand adjourned.

... Whereupon at 5:10 P.M. the meeting was adjourned ...
